# Post-transplant alloimmune liver disease in bile salt export pump deficiency: A multicenter study

**DOI:** 10.1016/j.jhepr.2026.101969

**Published:** 2026-07-25

**Authors:** Antoine Gardin, Chiara Rubino, Alice Michaut, Géraldine Résin, Marion Almes, Teresa Antonini, Patrick Borentain, Dominique Debray, Eleonora De Martin, Bogdan Hermeziu, Bertrand Roquelaure, Mathias Ruiz, Xavier Stéphenne, Martine Lapalus, Giuseppe Maggiore, Thomas Falguières, Charlotte Mussini, Emmanuel Gonzales, Emmanuel Jacquemin

**Affiliations:** 1 Pediatric Hepatology and Liver Transplantation Unit, Bicêtre Hospital, Assistance Publique - Hôpitaux de Paris (AP-HP), Paris-Saclay University, le Kremlin-Bicêtre, France; 2 Université Paris-Saclay, Univ Evry, Inserm, Genethon, Integrare research unit UMR_S951, Evry, France; 3 Liver Unit, Meyer Children's Hospital IRCSS, Florence, Italy; 4 Pathology Unit, Bicêtre Hospital, Assistance Publique - Hôpitaux de Paris, Paris-Saclay University, Le Kremlin-Bicêtre, France; 5 Université Paris-Saclay, Inserm, Physiopathogénèse et traitement des maladies du foie, UMR_S 1193, FHU Hepatinov, Orsay, France; 6 Hepatology Unit, Croix Rousse Hospital, Lyon, France; 7 Hepatology and Gastroenterology Unit, Timone Hospital, Marseille, France; 8 Pediatric Hepatology Unit, Necker Hospital, Assistance Publique - Hôpiaux de Paris, Paris, France; 9 Hepatology Unit, Paul Brousse Hospital, Assistance Publique - Hôpitaux de Paris, Villejuif, France; 10 Pediatric Gastroenterology and Hepatology Unit, Timone Hospital, Marseille, France; 11 Pediatric Gastroenterology and Hepatology Unit, Bron Hospital, Bron, France; 12 Pediatric Hepatology Unit, Saint-Luc Hospital, Louvain University, Bruxelles, Belgium; 13 Hepatogastroenterology and Liver Transplant Unit, Bambino Gesù Children's Hospital, Rome, Italy

**Keywords:** BSEP deficiency, PFIC2, Alloimmunization, Liver transplantation

## Abstract

**Background & Aims:**

Patients with severe bile salt export pump (BSEP) deficiency may develop alloimmune anti-BSEP liver disease (AIBD) after liver transplantation (LT) as a result of anti-BSEP antibodies, but its frequency and risk factors are not precisely known.

**Methods:**

A multicenter cohort of 35 patients who underwent LT for severe BSEP deficiency was screened retrospectively or prospectively after 2010 for anti-BSEP antibodies. Risk factors, management and outcome of AIBD were analyzed.

**Results:**

Ten patients (29%) were screened positive for anti-BSEP antibodies and all developed AIBD, in median 5 years (range: 1.5–14 years) after LT. Clinical remission of AIBD was achieved in nine patients using various immunosuppressive therapies, with decreased or negative anti-BSEP antibody titers in six patients, all having received rituximab or plasmapheresis/immunoadsorption. Relapse occurred in six of nine patients, although maintenance therapy using rituximab and/or immunoglobulins enabled prolonged relapse-free survival. Because of AIBD, six patients (60%) required liver retransplantation either at initial AIBD episode or at relapse, and three patients (30%) died. Among the 25 patients without AIBD, one died (4%) and another one was retransplanted (4%). Although a severe *ABCB11* genotype (biallelic protein-truncating variants) was significantly associated with AIBD (80% in patients with AIBD *vs*. 28% in patients without AIBD, *p* = 0.008), negative BSEP immunostaining on native liver, post-LT CMV infection, presence of biliary anastomosis strictures or graft rejection were not.

**Conclusions:**

In patients with severe BSEP deficiency, AIBD is a frequent post-LT complication that should be screened for, especially in patients with severe *ABCB11* genotypes. Early diagnosis and the use of rituximab and plasmapheresis/immunoadsorption may improve patient outcome.

**Impact and implications:**

Alloimmune anti-BSEP liver disease is a frequent and severe complication after LT in patients with BSEP deficiency, affecting nearly one-third of recipients in this multicenter cohort study. Patients carrying biallelic protein-truncating *ABCB11* variants are at particularly high risk and should undergo systematic post-transplant screening for anti-BSEP antibodies. Early recognition of alloimmune anti-BSEP liver disease and timely treatment with B-cell-depleting and antibody-removal therapies, including rituximab and plasmapheresis/immunoadsorption, may improve outcomes and reduce graft loss. These findings support the implementation of structured surveillance strategies and risk-adapted management in this rare but life-threatening condition.

## Highlights

• Alloimmune anti-BSEP liver disease (AIBD) affects one-third of patients with BSEP deficiency after liver transplantation.
• The presence of severe biallelic *ABCB11* variants is a major risk factor of AIBD.
• AIBD significantly decreases long-term graft and patient survival.
• Treatment with rituximab and/or plasmapheresis/immunoadsorption enables AIBD remission in most patients.
• Maintenance therapy using rituximab and/or intravenous immunoglobulins reduces AIBD relapse.

## Graphical abstract

**Figure:**
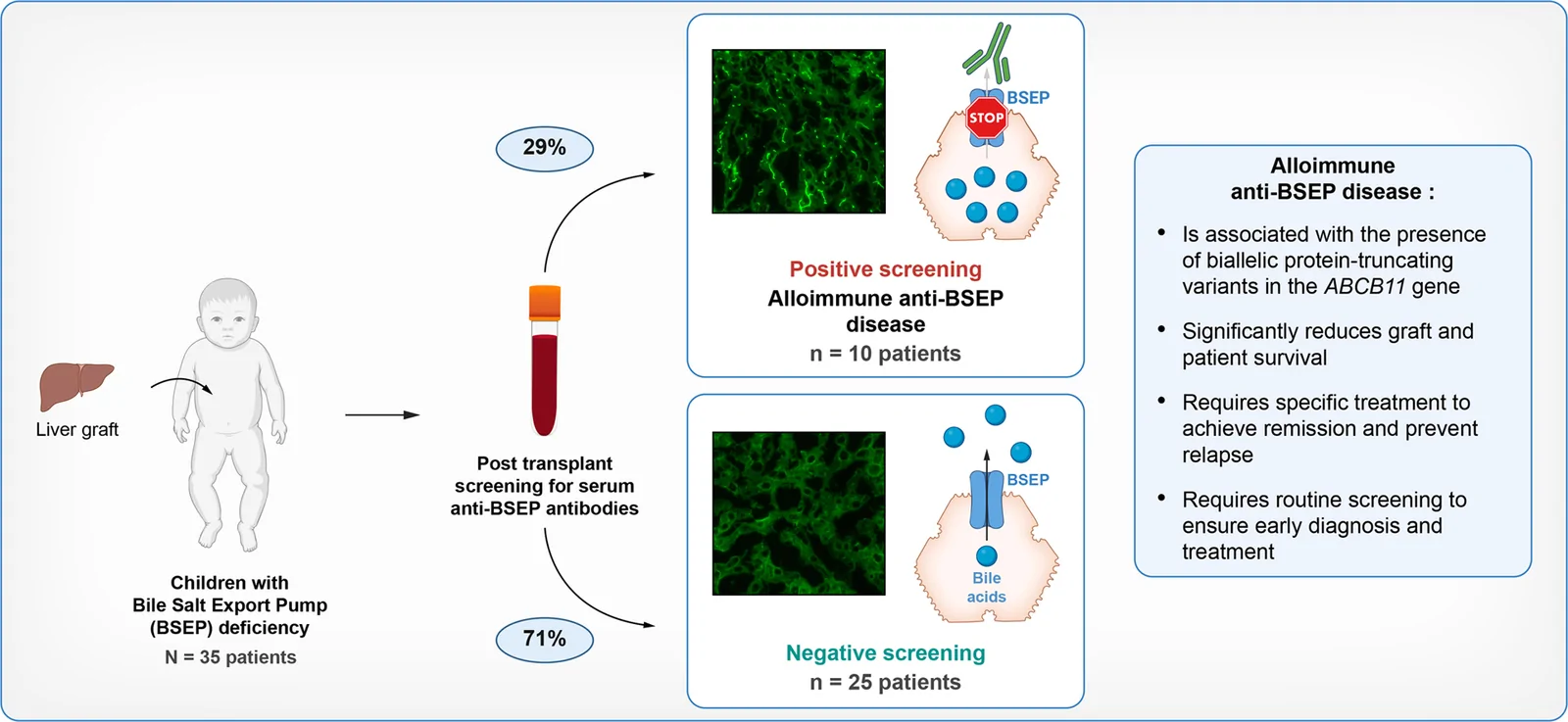

## Introduction

Severe bile salt export pump (BSEP) deficiency, also known as progressive familial intrahepatic cholestasis type 2 (PFIC2), is an autosomal recessive genetic disease caused by biallelic variants in the *ABCB11* gene, which encodes BSEP.^1^^,^^2^ BSEP is a transporter exclusively expressed at the canalicular membrane of hepatocytes and exports bile acids into the bile canaliculus. Severe BSEP deficiency causes a dramatic reduction in bile salt secretion into the bile canaliculus, resulting in cholestasis.^1^^,^^2^

Children with severe BSEP deficiency typically present with jaundice, pruritus and failure to thrive within the first months of life.^1^^,^^2^ Serum liver tests reveal elevated serum conjugated bilirubin, alanine aminotransferase (ALT) and bile acid (sBA) levels and normal gamma-glutamyl transpeptidase (γGT) level.[1], [2], [3] When measured, biliary bile acid concentration is dramatically low.^1^^,^[3], [4] Liver biopsy shows intense hepatocellular and canalicular cholestasis with giant cell transformation of hepatocytes.^1^^,^^3^ Immunohistochemical study of BSEP expression shows a decreased or negative canalicular expression in most patients. Patients carrying biallelic variants predicted to result in a complete absence of expression of functional BSEP at the canalicular membrane present with early-onset cholestasis rapidly progressing toward hepatic fibrosis, cirrhosis, and end-stage liver disease.^5^

Such children with severe *ABCB11* genotypes suffer from intractable pruritus, which is not responding to ursodeoxycholic acid therapy or biliary diversion, and are at high risk for hepatocellular carcinoma.^3^^,^[5], [6], [7], [8], [9] In patients with severe BSEP deficiency, liver transplantation (LT), indicated in case of end-stage liver disease, evidence of malignancy or severe pruritus not responding to medical therapy, was considered as a definitive curative treatment.^3^^,^^10^ A first report of an unexplained recurrence of a low-γGT PFIC phenotype after LT was described in the 1990s.^11^ After the genetic cause of PFIC2 was identified, the hypothesis of a post-LT anti-BSEP alloimmunization was proposed to account for this recurrence.^1^ In the same year, the presence of anti-BSEP antibodies able to inhibit bile secretion was reported in the serum of four patients with severe BSEP deficiency and phenotypic recurrence of PFIC2 after LT.^12^^,^^13^ Although a growing number of case reports and small series have provided evidence that alloimmune anti-BSEP liver disease (AIBD) is a substantial cause of graft loss and of mortality, no standardized screening and treatment protocols have been established.[14], [15], [16], [17], [18], [19], [20], [21], [22], [23], [24], [25], [26], [27], [28], [29], [30], [31]

In this retrospective multicenter study, we aimed to assess the prevalence of AIBD in a cohort of patients receiving LT for severe BSEP deficiency and describe their clinical, laboratory and histological features, as well as their outcome and response to treatments. In addition, we compared genotypes, clinical features, and histological characteristics of patients with and without AIBD.

## Patients and methods

### Patients

We retrospectively collected data from patients receiving LT for severe BSEP deficiency before the age of 18 years between 1988 and 2024 at Bicêtre Hospital (Le Kremlin-Bicêtre, France, n = 22), Femme-Mère Enfants Hospital (Bron, France, n = 6), La Timone Hospital (Marseille, France, n = 5), Necker Enfants Malades Hospital (Paris, France, n = 1), and Cliniques Universitaires Saint-Luc (Brussels, Belgium, n = 1). All patients had biallelic variants in the *ABCB11* gene and underwent at least one testing for anti-BSEP antibodies in serum after LT. Of note, three patients (patients #3, #6, and #7) were previously reported.^14^^,^^28^ Following its first description in 2009, AIBD screening was progressively implemented in France. Since 2010, all patients with severe BSEP deficiency receiving LT in French hospitals have been prospectively screened for AIBD, whereas sera sampled before 2010 were retrospectively analyzed for AIBD, when available. In Belgium, screening for anti-BSEP antibodies in patients with severe BSEP deficiency was performed in case of suspicion of AIBD. AIBD was defined as the presence of anti-BSEP antibodies in serum, regardless of their titer. In all patients, we retrospectively collected *ABCB11* genotype, type of LT, immunosuppressive (IS) regimen and compliance, LT complications including rejection, viral infections, biliary anatomosis stenosis, as well as liver histological data. Patient characteristics are described in Tables S1 and S2. BSEP and C4d immunostaining were performed on liver slides (antibody reference: HPA019035, Sigma (Darmstadt, Germany), 1:1,500 dilution for BSEP and DB-107 clone A24-T, DB Biotech (Kosice, Slovakia), 1:100 dilution for C4d). *ABCB11* genotypes were classified as severe (biallelic protein-truncating variants, *i.e*. nonsense, frameshift, and/or splicing variants), moderate (one severe and one missense variant), or mild (biallelic missense variants). One small in-frame deletion was considered as a missense variation. Before LT, all patients were treated with ursodeoxycholic acid (daily oral dose of 600 mg/m^2^ of body surface area), rifampicin (daily oral dose up to 20 mg/kg); four patients (patients #11, #12, #34, #35) were also treated with 4-phenylbutyrate with some efficacy in two of them (patients #12, #35). For patients developing AIBD, we collected the evolution of clinical and biological manifestations, management, and outcome. Immunological remission was defined as the loss of detectable serum anti-BSEP antibodies in patients with previously established AIBD. Clinical remission was defined as resolution of clinical manifestations and normalization of serum liver tests, including sBA, except when abnormalities could be explained by another complication of LT (biliary obstruction, chronic rejection). AIBD relapse was defined either as a recurrence of anti-BSEP antibodies in serum, or as an increase of anti-BSEP antibody titer in serum associated with recurrence of symptoms (pruritus, jaundice, low-γGT cholestasis).

Approval was obtained from the institutional review board of our institution (AP-HP, N°00011558). According to French legislation, ethics committee agreement is not required for the retrospective collection of data corresponding to current practice. The study conformed to the ethical guidelines of the 1975 Declaration of Helsinki. All patients were informed about the study and none of them refused to be included in the study.

### Anti-BSEP antibodies screening and confirmation of anti-BSEP antibodies specificity

All sera were tested in the Pathology Unit of Bicêtre Hospital using indirect immunofluorescence, as previously described.^14^ Briefly, cryosections of normal human liver were first incubated with serum samples and bound antibodies were detected using fluorescein isothiocyanate-tagged rabbit anti-human immunoglobulin G (polyclonal FITC-conjugate rabbit anti-human IgG antibody, Dako, [Copenhagen, Denmark] ref F0202). Positive canalicular staining, suggesting the presence of anti-BSEP IgG antibodies bound to the canalicular membrane, was used to diagnose AIBD. When possible, positive serum samples were retested after serial dilution (1/10, 1/50, 1/100, 1/200) to determine an antibody titer. Known positive and negative serum samples were used as controls (Fig. S1). To confirm the anti-BSEP antibodies specificity, indirect immunofluorescence analysis of polarized Can10 cells transduced with rat Bsep-GFP fusion protein incubated with the patient serum was performed, as previously described.^32^ Colocalization of the anti-BSEP antibodies (revealed using Alexa Fluor™ 568-conjugated anti-human IgG antibody, Thermofisher Scientific, ref A-21090, dilution 1/500^e^), and of GFP (revealed using anti-GFP monoclonal mouse antibody, Roche (Basel, Swiss) Ref 11814460001, dilution 1/80^e^; and secondary Alexa Fluor™ 488-conjugated anti-mouse IgG antibody, Thermofisher Scientific (Waltham, MA, USA), ref A-11001, dilution 1/500^e^) confirmed the anti-BSEP specificity. Antigenic specificity of anti-BSEP antibodies was studied by ELISA using three peptides designed from potentially immunogenic regions of BSEP: the N-terminal region (amino-acids 21-35; B0 peptide), the first extracellular loop (amino-acids 115-129; B1 peptide), and the C-terminal region of the protein (amino-acids 1297-1312; B2 peptide; described in detail in the Supplementary Methods).

### Statistical analysis

Results were summarized as medians and IQRs for continuous variables, and frequency (percentages) for nominal variables. Continuous variables were compared by means of the Mann–Whitney *U* test. The Fisher exact test was performed to evaluate differences between categorical variables. The value of *p* <0.05 was considered statistically significant. Survival curves were evaluated using Kaplan–Meier estimates, using R software (version 4.4.2, R Foundation for Statistical Computing, Vienna, Austria).

## Results

### Systematic screening for anti-BSEP antibodies identified ten patients with AIBD

**Fig. 1:**
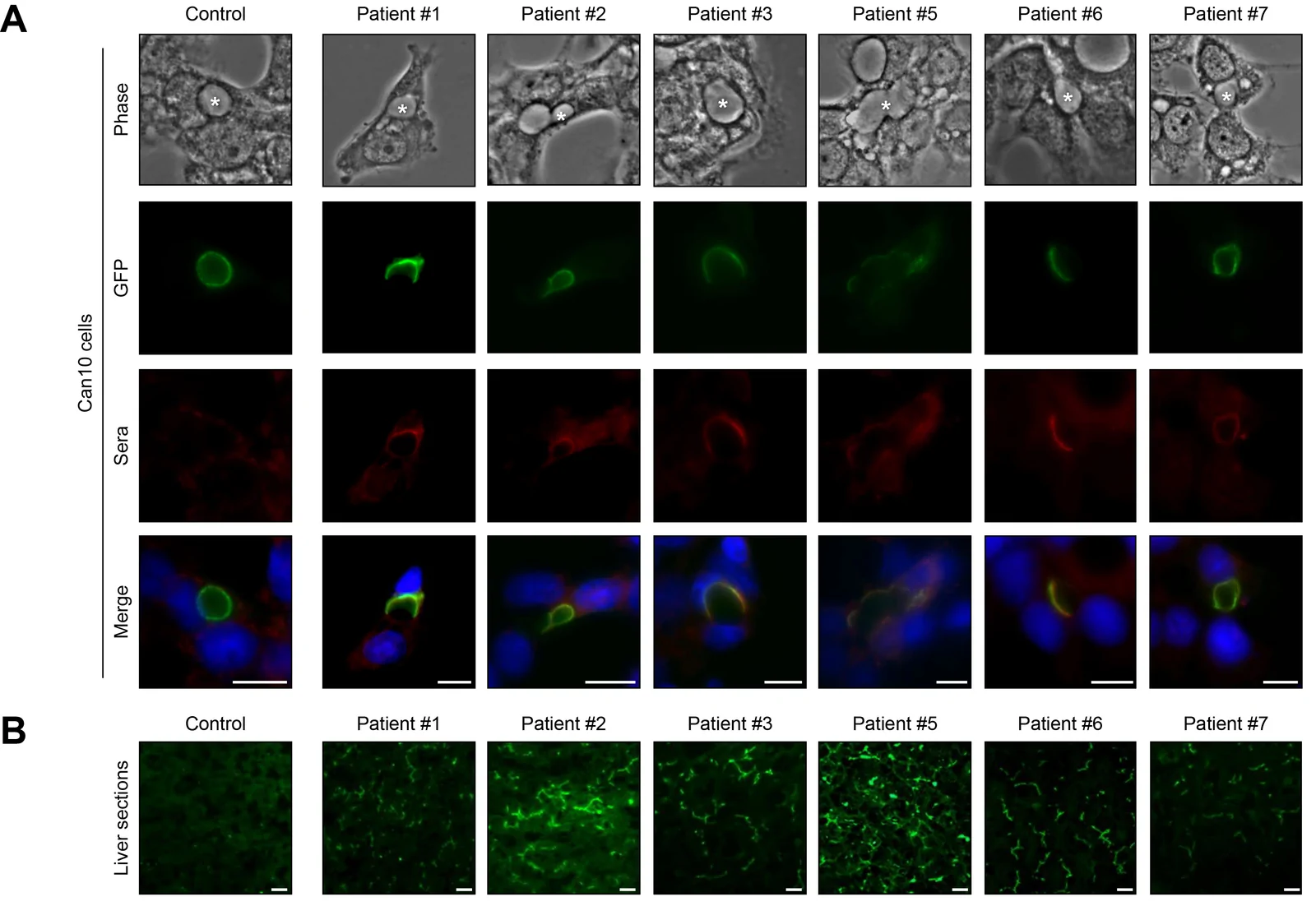
Indirect immunofluorescence analysis of serum samples of patients with AIBD. (A) Can10 cells transfected with rat Bsep-GFP fusion protein were incubated with patient sera. Putative anti-BSEP antibodies were revealed by the addition of a red fluorochrome-tagged anti-human IgG antibody. GFP was revealed using an anti-GFP monoclonal mouse antibody and a secondary green fluorochrome-tagged anti-mouse antibody. Colocalization of both red and green signals at the canalicular membrane confirmed the specificity of the antibodies against the BSEP protein. Asterisks indicate canaliculi. (B) Indirect immunofluorescence performed on cryosections of normal human liver incubated with patient sera. Use of a green fluorochrome-tagged-anti-human IgG secondary antibody revealed a canalicular membrane staining suggestive of the presence of anti-BSEP antibodies in sera. BSEP, bile salt export pump; GFP, green fluorescent protein.

In total, 35 children received LT for severe BSEP deficiency between 1988 and 2024 in France (n = 34, including 19 patients between 1988 and 2009 and 15 patients between 2010 and 2024) and in Belgium (n = 1). The systematic screening of serum anti-BSEP antibodies was implemented in France in 2010. Therefore, all patients transplanted after 2010 (n = 15) had systematic screening of sera from the time of LT. Regarding patients who underwent LT before 2010 (n = 19), all patients had systematic screening of anti-BSEP antibodies in sera obtained after 2010, whereas retrospective sera could be retrieved and analyzed in only 10 of these 19 patients. In the French cohort (n = 34), serum anti-BSEP antibodies were identified in nine of the 34 patients (26%), in either prospectively collected sera (n = 6) or retrospectively collected sera (n = 3). One additional patient from Belgium, presenting with recurrence of a cholestatic disease after LT, also had anti-BSEP antibodies in serum. Therefore, 10 of 35 patients (29%) were considered as having AIBD. The anti-BSEP specificity of the antibodies was demonstrated in all the six patients tested (Fig. 1). The sera of five patients were further studied to assess the specificity of anti-BSEP antibodies toward selected epitopes using ELISA (Fig. S2). In all cases, reactivity was demonstrated against the first extracellular loop (B1 peptide) and the C-terminus part of BSEP (B2 peptide), similar to previous observations,^19^ but not against the N-terminus region of BSEP.

### Patients with severe *ABCB11* genotype are more likely to present AIBD

Main characteristics of the 10 patients (three females) who developed AIBD and of the 25 patients (14 females) who did not develop AIBD are described in detail in Tables S1 and S2 respectively, and summarized in Table 1. Before LT, the severity of the manifestations of BSEP deficiency was similar in both groups, as indicated by similar median ages at first symptoms (3.5 *vs.* 3 months of age, *p* = 0.60) and at LT (4.7 *vs.* 7.0-year-old, *p* = 0.47) and a similar proportion of patients who underwent LT for a severe complication of BSEP deficiency such as cirrhosis, liver failure, and/or hepatocellular carcinoma (70% *vs.* 68%, *p* = 1.0). Severe *ABCB11* genotype appears to be the major risk factor of AIBD, with most of the patients with AIBD harboring a severe genotype (80%), compared with 28% of patients without AIBD (*p* = 0.01). In our cohort, eight of 15 patients (53%) with severe *ABCB11* genotypes developed AIBD, in contrast to two of 13 (15%) with moderate genotypes and none with mild genotypes. By contrast, BSEP immunostaining assessed in native liver did not predict AIBD, as it was negative in all patients with AIBD (n = 8/8) and in 90% of patients without AIBD (n = 19/21, *p* = 1.0). No difference in type of donor, IS treatment, or post-LT complications (T-cell-mediated rejection, biliary obstruction, presence of donor-specific antibodies) were identified, nor more severe ischemia–reperfusion injuries (data not shown), except a higher occurrence of cytomegalovirus (CMV) infection in the group of patients with AIBD which almost reached significance (20% *vs.* 0%, *p* = 0.08). Duration of follow-up was 14.2 and 12.6 years post-LT in median in both groups.

**Table 1:** Main characteristics of patients with and without AIBD following liver transplantation for severe BSEP deficiency.

	Patients with AIBD (n = 10)	Patients without AIBD (n = 25)	*p* values
Female sex	3/10 (30)	14/25 (56)	0.26
Age at initial symptoms (in months)	3.5 (2.3–7.3)	3.0 (1.0–5.1), n = 20	0.60
*ABCB11* genotype∗			
Severe	8/10 (80)	7/25 (28)	**0.01**
Moderate	2/10 (20)	11/25 (44)	
Mild	0/10	7/25 (28)	
Absent BSEP staining on native liver	8/8 (100)	19/21 (90)	1.0
Age at LT (years)	4.7 (3.0–8.1)	7.0 (3.5–9.4)	0.47
Indication for LT			
Severe pruritus	3/10 (30)	8/25 (32)	1.0
Complication of BSEP deficiency (cirrhosis, LF, HCC)	7/10 (70)	17/25 (68)	
Deceased donor	9/10 (90)	22/25 (88)	1.0
Immunosuppression after LT			
Tacrolimus-based	5/9 (56)	20/25 (80)	0.20
Cyclosporine-based	4/9 (44)	5/25 (20)	
Combination with Aza or MMF (within the first year after LT)	6/9 (67)	12/25 (48)	0.45
Complications after LT (besides AIBD)			
Acute T-cell-mediated rejection	7/10 (70)	16/25 (64)	1.0
BA stricture	5/10 (50)	8/25 (32)	0.44
Symptomatic CMV infection	2/10 (20)	0/25	0.08
Positive DSA†	2/7 (29)	6/17 (35)	1.0
Duration of follow-up after LT (years)	14.2 (10.2–24.5)	12.6 (6.2–15.6)	**0.03**
Retransplantation	6/10 (60)	1/25 (4)	**0.0008**
Death	3/10 (30)	1/25 (4)	0.06

Data are presented as n/N (%) or median (IQR). The *p* values <0.05 are indicated in bold. AIBD, alloimmune anti-BSEP liver disease; Aza, azathioprine; BA, biliary anastomosis; BSEP, bile salt export pump; CMV, cytomegalovirus; DSA, donor-specific antibodies; HCC, hepatocellular carcinoma; LF, liver failure; LT, liver transplantation; MMF, mycophenolate mofetil.

∗ Genotypes defined as severe (biallelic protein-truncating variants), moderate (one protein-truncated variant and one missense variant), and mild (biallelic missense variant).

† Positive DSA are HLA class II donor-specific antibodies with mean fluorescence intensity above 1,000. Statistical analysis was performed using Fisher exact test (qualitative variables, including 3 × 2 Fisher test for *ABCB11* genotype analysis) or Mann–Whitney *U* test (quantitative variables).

### AIBD initially manifests as intrahepatic cholestasis with pruritus

The initial clinical and laboratory characteristics of the 10 patients developing AIBD are detailed in Table 2. The median age at AIBD diagnosis and interval between LT and AIBD were 11.1 years (IQR: 8–16, range 4.2–20.7 years) and 5 years (IQR: 3.7–8.3, range: 1.5–12 years), respectively. Notably, for patient #2, AIBD was confirmed 1 year after a second LT, performed for graft loss following recurrence of a cholestatic disease compatible with AIBD. Before the first serum positivity, a decrease of IS exposure was identified in four cases, because of an adjustment of the IS regimen (patients #3 and #7) or because of non-adherence to treatment (patients #4 and #9). Nine of 10 patients had symptoms at the time of first detection of anti-BSEP antibodies in serum, including pruritus (n = 9), jaundice (n = 5) and hypocholic stools (n = 3). One patient (#10) experienced AIBD-related manifestations 2 years after detection of anti-BSEP antibodies. All patients tested had elevation of sBA at the time they experienced clinical manifestations of AIBD, with a median sBA concentration of 244 μmol/L (range: 44–587), contrasting with low values of serum γGT level (<40 U/L in eight of 10 patients) (Table 2). By contrast, the median sBA concentration was 10 μmol/L (range: 4–43 μmol/L) during follow-up in the 10 patients without AIBD in whom the data were available.

**Table 2:** Main clinical and laboratory features at diagnosis of AIBD.

Patient	*ABCB11* genotype	Interval from LT (years)	Events in the preceding 3 months	Symptoms (at diagnosis of AIBD)	Liver function tests (at diagnosis of AIBD)				Anti-BSEP antibodies (titer)
					ALT (U/L)	γGT (U/L)	Total/conjugated bilirubin (μmol/L)	sBA (μmol/L)	
#1	Severe	4.9	UDCA treatment interruption	Pruritus, jaundice, hypocholic stools	36	12	77/66	368	Positive (1/50)
#2	Moderate	8.7 (1 yr after reLT)	None	Pruritus, jaundice	739	36	349/NA	190	Positive (NA)
#3∗	Moderate	3.3	Decrease of IS	Pruritus	160	35	32/NA	107	Positive (NA)
#4	Severe	5.1	Non-compliance to IS regimen	Pruritus	727	66	23/20	60	Positive (1/200)
#5∗	Severe	3.2	Biliary obstruction, recurrent cholangitis episodes	Pruritus	58	22	11	44	Positive (1/1)
#6	Severe	12.5	None	Pruritus, jaundice	184	50	287/152	533	Positive (1/10)
#7∗	Severe	4.8	Decrease of IS	Pruritus, hypocholic stools	31	14	32/22	298	Positive (1/10)
#8	Severe	7	Jejunal intussusception after a viral infection, TCMR	Pruritus, jaundice	77	37	103/NA	NA	Positive (NA)
#9	Severe	1.5	Non-compliance to IS regimen	Pruritus, jaundice, hypocholic stools	28	13	186/176	587	Positive (1/200)
#10	Severe	12	None	No symptoms	36	8	21/8	6.2	Positive (1/10)
		14	None	Pruritus & jaundice	525	13	169/119	NA	Positive (1/200)

Upper limit of normal: <40 U/L (ALT), <40 U/L (γGT), <17 μmol/L (bilirubin), and <10 μmol/L (sBA). ∗Indicates patients diagnosed retrospectively. AIBD, alloimmune anti-BSEP liver disease; ALT, alanine aminotransferase level; BSEP: bile salt export pump; γGT, γ-glutamyl transferase level; IS, immunosuppression; LT, liver transplantation; NA, not available; reLT, liver retransplantation; sBA, serum bile acids level; TCMR, T-cell-mediated rejection; UDCA, ursodeoxycholic acid.

### AIBD mimics histological features of severe BSEP deficiency and is associated with positive canalicular C4d staining

**Fig. 2:**
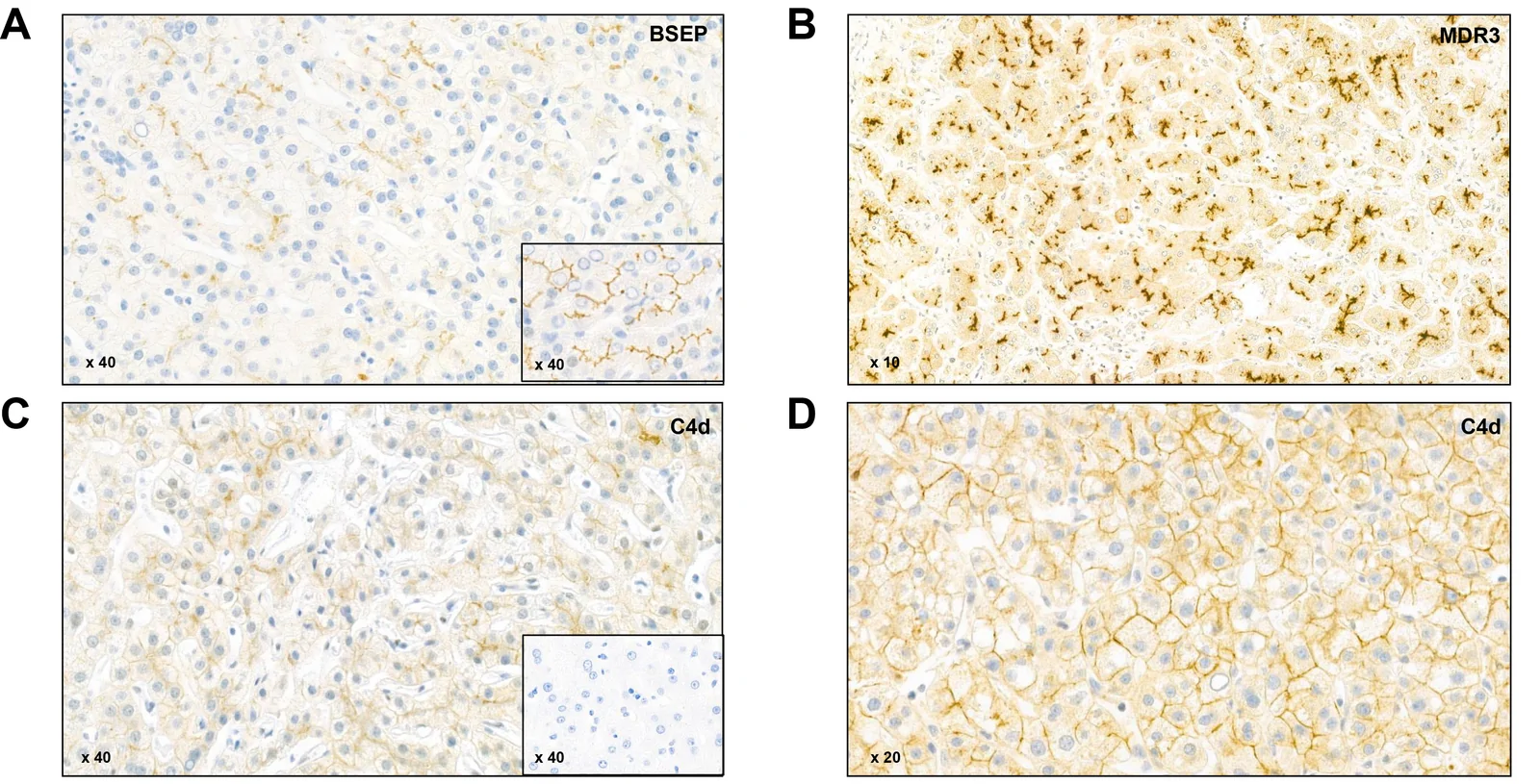
Representative images of reduced BSEP staining and of positive canalicular C4d staining in a patient with AIBD. (A,B) Reduced canalicular BSEP staining (A) on the biopsy of patient #1 at AIBD relapse, contrasting with a conserved canalicular MDR3 staining (B). In panel A, insert shows a normal positive BSEP staining as control. (C,D) C4d staining on the biopsy of patient #1 at relapse (C) and 1 month after relapse (D), showing focal canalicular C4d staining (C) and diffusely membranous C4d staining with canalicular enhancement (D). Insert shows a negative C4d staining as control (from a patient without AIBD). AIBD, alloimmune anti-BSEP liver disease; BSEP, bile salt export pump; C4d, complement component 4d.

Liver biopsies were analyzed in nine patients at AIBD onset (Table 3). Most patients exhibit canalicular cholestasis and giant transformation of hepatocytes (n = 7/9 for each, 78%), mimicking the phenotype of patients with severe BSEP deficiency (Fig. S3). Three patients had concomitant moderate T-cell-mediated rejection (TCMR). Fibrosis was moderate with liver allograft fibrosis score (LAFSc) between 2 and 5 in the six biopsies evaluated. Finally, BSEP immunostaining was reduced in four of seven patients tested, while MDR3 was not (Fig. 2A,B), and revealed normal canalicular BSEP expression in three patients. Anti-C4d immunostaining performed in five patients did not show signs of chronic antibody-mediated rejection (AMR), but revealed focal or diffuse canalicular staining in four patients (Fig. 2C,D). Analysis of follow-up liver biopsies and explanted livers is described in Table S3, further demonstrating that AIBD histological features mimic the one of severe BSEP deficiency and that canalicular positive C4d staining is frequently observed along the course of AIBD (five of six patients tested).

**Table 3:** Liver graft histological characteristics at diagnosis of AIBD.

Patient	Time after LT (years)	Histological features	Fibrosis (LAFSc)	TCMR (BANFF)	BSEP immunostaining	C4d immunostaining	AMR
#1	4.9	Severe canalicular cholestasis; rare giant hepatocytes; mild centrilobular and portal inflammation; biliary obstruction	4 (2 + 0 + 2)	No	**Reduced canalicular expression**	**Positive** (canalicular, lateral)	No
#2∗	9.0	**Explanted liver**: severe cholestasis, giant hepatocytes	7 (2 + 2 + 3)	No	Normal canalicular expression	NA	NA
#4	5.2	Lobular and portal hepatitis; rare giant hepatocytes	2 (1 + 1 + 0)	Yes (6: 3 + 1 + 2)	Normal canalicular expression	**Positive** (canalicular)	No
#5	4.8	Moderate portal and mild perivascular inflammation	2 (1 + 0 + 1)	No	Normal canalicular expression	NA	Yes
#6	13	Canalicular cholestasis; giant hepatocytes Moderate portal inflammation with ductular proliferation.	5 (3 + 2 + 0)	Yes (3: 2 + 0 + 1)	**Reduced canalicular expression**	Negative	No
#7∗	4.8	Canalicular cholestasis, giant hepatocytes Slight centrilobular fibrosis	NA	NA	NA	NA	NA
#8	7	Canalicular cholestasis, giant hepatocytes Portal inflammation	3 (1 + 2 + 0)	Yes (5: 2 + 2 + 1)	**Reduced canalicular expression**	**Positive** (canalicular)	No
#9∗	1.5	Cholestasis with ductopenia and bile duct dystrophy	NA	NA	NA	NA	NA
#10	14.3	Cholestasis; giant hepatocytes	NA	No	**Reduced canalicular expression**	**Positive** (canalicular)	No

Liver biopsies were performed at diagnosis. For patient #10, biopsy was performed 2 years after initial positivity of anti-BSEP antibodies, at the onset of symptoms. LAFSc is expressed as the sum of a 0–3 score referring to portal tract, sinusoids, and central vein fibrosis (total score: 0-9).^39^ TCMR was graded using BANFF score, expressed as the sum of a 0–3 score referring to portal tract inflammation, bile duct inflammatory lesions, and venous endothelial inflammation (max 0–9).^40^ All biopsies were reviewed by a single pathologist of Bicêtre Hospital, unless indicated by the symbol ‘∗’. AIBD, alloimmune anti-BSEP liver disease; AMR, antibody-mediated rejection; BSEP, bile salt export pump; C4d, complement component 4d; LAFSc, liver allograft fibrosis score; LT, liver transplantation; NA, not available; TCMR, acute T-cell-mediated rejection.

By contrast, study of the 17 biopsies performed after LT in 11 patients who did not develop AIBD (Table S4) did not show positive canalicular C4d staining, identified cholestasis in only two patients, giant transformation of hepatocytes in one patient (early after LT), and reduced BSEP staining in one patient. Therefore, the presence of giant hepatocytes, reduced BSEP immunostaining, and/or positive canalicular C4d staining may strengthen the suspicion of AIBD in patients receiving LT for severe BSEP deficiency.

### AIBD has a severe outcome and requires specific intensification of immunosuppressive therapy

**Fig. 3:**
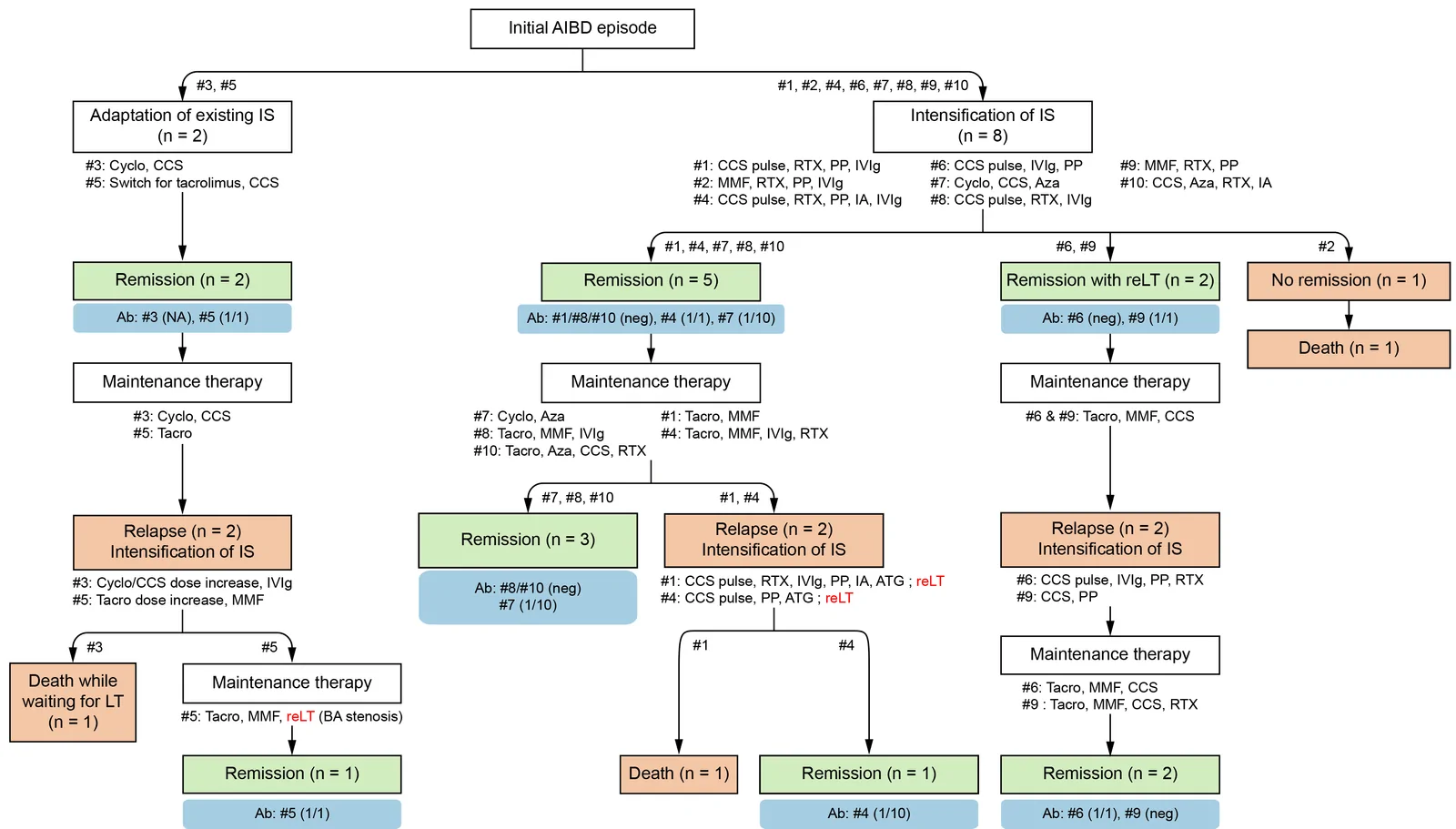
Treatment and outcome of the 10 patients with AIBD. Remission was defined as clinical remission (resolution of clinical manifestations and normalization of serum liver tests, including serum bile acid levels, except if abnormalities could be explained by another complication of liver transplantation such as biliary obstruction or chronic rejection). Ab, anti-BSEP antibody titers; AIBD, alloimmune anti-BSEP liver disease; ATG, anti-thymocyte globulins; Aza, azathioprine; BA, biliary anastomosis; CCS, corticosteroids; Cyclo, cyclosporine; IA, immunoadsorption; IS, immunosuppression; IVIg, intravenous immunoglobulins; LT, liver transplantation; MMF, mycophenolate mofetil; NA, not available; PP, plasmapheresis; reLT, liver retransplantation; RTX, rituximab; Tacro, tacrolimus.

Management of AIBD is detailed in Table S5 and summarized in Fig. 3. Of note, the three patients diagnosed with AIBD retrospectively were initially considered as having rejection episodes with adaptation (patients #3 and #5) or intensification (patient #7) of IS regimen. The seven other patients, whose serum positivity was detected prospectively, underwent intensification of IS regimen based on a combination of rituximab (n = 6), plasmapheresis (n = 5), intravenous immunoglobulins (IVIg) (n = 5), pulse steroids (n = 4), and/or immunoadsorption (IA) (n = 2). Two of these seven patients required retransplantation (reLT) for graft failure early after AIBD diagnosis (patients #6 and #9).

Clinical remission was achieved in nine of 10 patients, including one patient (patient #8) with persistent ALT and γGT increase attributable to chronic TCMR while displaying negative anti-BSEP antibodies. The single patient (patient #2) for whom remission was not obtained was diagnosed with AIBD 1 year after reLT for graft loss likely because of unrecognized AIBD. He did not respond to intensification of IS and died of recurrent graft failure as a result of AIBD. In patients who achieved clinical remission, anti-BSEP antibody titers were stable in two (retrospectively diagnosed patients #5 and #7), decreased in two (patients #4, #9), or were negative in four (patients #1, #6, #8, #10). Of note, decreased or negative anti-BSEP antibody titers were only observed in patients who had received either rituximab and/or plasmapheresis/IA.

Several IS regimens were used following the first episode of AIBD and were considered as ‘maintenance therapies’ (Fig. 3), including repeated IVIg (patients #4, #8) and/or rituximab administrations (patients #4 and #10) in three patients. Interestingly, only one of these three patients had a relapse of AIBD (patient #4), favored by a complete interruption of all IS therapy (including tacrolimus and steroids). This patient had very low anti-BSEP antibody titers (1/1) before IS interruption. In the two other patients (patients #8 and #10), prolonged remission with negative anti-BSEP antibody titers was achieved. By contrast, five of the six patients who did not receive IVIg or rituximab as a maintenance therapy exhibited a relapse of AIBD. Some correlation between B-cell depletion and anti-BSEP antibody titers could be observed, with recurrence of high anti-BSEP antibodies (titer >1/100) in patient #1 and #4 at relapse associated with B-cell counts ≥10%.

Overall, six patients exhibited a relapse of AIBD in median 6 years (range: 1.7–14 years) after the initial episode. An intensification of IS therapy was performed in all patients. It enabled clinical remission in four of them while the two other patients (patients #1 and #3), developed progressive liver failure and died because of AIBD, despite reLT in one (patient #1). Among the four patients who achieved clinical remission after relapse, only one (patient #9) achieved immunological remission, which was maintained with repeated rituximab administrations. Anti-BSEP antibody titers decreased in the three other patients. Two of them required a reLT for graft failure because of AIBD and IS interruption (patient #4) or for cirrhosis with concomitant biliary obstruction (patient #5). Detailed evolution of serum liver tests and serum anti-BSEP antibody titers of patients #1, #4, #8, #9, and #10 are provided in Fig. S4.

**Fig. 4:**
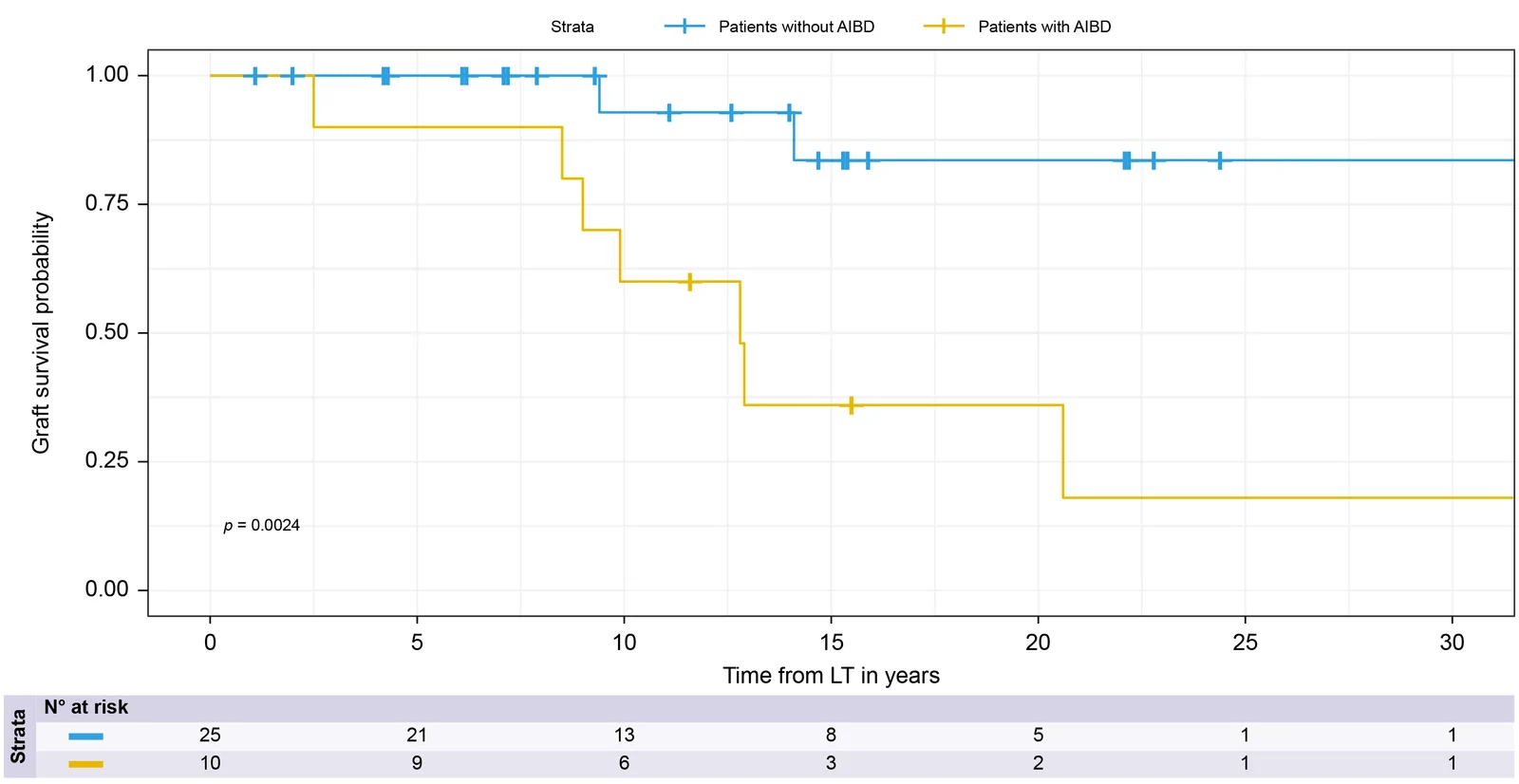
Graft survival curve of the 35 patients according to the presence of AIBD. Patients were censored at the time of retransplantation or death. Survival curve was made using Kaplan–Meier estimates, using the R software (version 4.4.2; R Foundation for Statistical Computing, Vienna, Austria). Log-rank test was used for comparison. AIBD, alloimmune anti-BSEP liver disease; LT, liver transplantation.

In total, six patients (60%) with AIBD required reLT, in contrast to one of 25 (4%) in the group of patients without AIBD (*p* = 0.0008). Three patients with AIBD (30%) and one patient without AIBD (4%) died (Table 1). Significantly poorer graft survival is observed in patients with AIBD, compared with patients without AIBD (*p* = 0.0024), as depicted in Fig. 4.

## Discussion

The present work, studying a cohort of patients with severe BSEP deficiency in whom serum anti-BSEP antibodies screening was systematically performed after LT, enables the estimation of the frequency of AIBD and the identification of its risk factors. In addition, this large cohort of patients with AIBD also provides insights into the outcome of AIBD and the impact of therapeutic interventions.

The frequency of AIBD observed in our cohort (29%) is in line with previous studies reporting a prevalence ranging between 8% and 33%.^15^^,^^31^^,^^33^ These data show that AIBD is, in fact, a frequent complication after LT in patients with severe BSEP deficiency, occurring in up to 50% of patients with severe *ABCB11* genotype. Therefore, patients with severe BSEP deficiency, and their family, should be informed before LT of the risk of AIBD. The mechanism responsible for anti-BSEP antibodies development after LT is unknown but likely involves multiple pathophysiological processes.^13^^,^^15^^,^^21^^,^^25^^,^^33^ It has been suggested that severe *ABCB11* genotypes, that is genotypes leading to the complete absence of BSEP protein expression, prevent the establishment of immune tolerance toward BSEP before LT, therefore favoring AIBD after LT.^15^^,^^25^^,^^33^ Our results, demonstrating that a severe *ABCB11* genotype is the major risk factors of AIBD, support this hypothesis. By contrast, a negative liver BSEP immunostaining did not predict the occurrence of AIBD. One hypothesis would be that a complete absence of BSEP expression or an intracellular retention of abnormal BSEP similarly lead to a negative BSEP immunostaining, but may result in different presentation of BSEP antigens to the immune system. Pre-LT expression of BSEP antigens, for example in patients with missense variants leading to intracellular retention of BSEP, would be required to achieve immune tolerance toward BSEP. In line with this, no patients with normal BSEP immunostaining on native liver have been described in the literature as having developed AIBD.

Other factors beyond genotypes are likely involved in the development of AIBD, as evidenced by the fact that several patients with and without AIBD shared common (patients #8/#10 and #19; #1 and #33) or equivalent genotypes (patients #3 and #15). The rather long time interval between LT and AIBD observed in our study, as in others,^33^ further suggests that a ‘second hit’ is required to trigger AIBD. Our data suggest that reduction of the IS regimen, as previously reported,^12^^,^^19^^,^^25^ and CMV infection could have contributed to the onset of AIBD. Our data do not provide evidence for a contribution of TCMR or severity of ischemia–reperfusion injuries to the onset of AIBD.

In line with literature data,^33^ most patients with AIBD present with a clinical phenotype mimicking the one of severe BSEP deficiency (*i.e*. a normal-γGT cholestasis associated with severe pruritus and high elevation of sBA) both at onset and at the time of relapse. The observation of such manifestations during the post-LT course raises suspicion of AIBD and should prompt the screening for anti-BSEP antibodies in the serum. In any case, considering the frequency and the severity of AIBD, systematic screening for anti-BSEP antibodies should be performed routinely even in the absence of AIBD manifestations, especially in patients with severe *ABCB11* genotypes.^34^ Furthermore, our data suggest that anti-BSEP antibodies may precede AIBD symptoms (patient #10).

Liver biopsy is a central tool in the management of liver transplanted patients in case of abnormal serum liver tests. In patients who received LT for severe BSEP deficiency, graft biopsy may provide evidences for AIBD showing canalicular cholestasis with giant cell transformation and positive canalicular C4d staining, while ruling out potential differential diagnosis. A positive canalicular C4d staining was rather sensitive and specific of AIBD in our study, in line with previous reports.^23^^,^^29^ This likely reflects the central pathophysiological role of anti-BSEP antibodies at the canalicular membrane in AIBD. By contrast, BSEP immunostaining on liver graft was already reported to be either absent,^14^ reduced,^14^^,^^23^^,^^26^^,^^29^ or conserved,^12^^,^^16^ depending on the studies. This variability might be due to a potential competition of alloimmune anti-BSEP antibodies, present in the patient sera, with the different commercial or in-house anti-BSEP antibody clones used for BSEP immunostaining. Some of these antibodies, such as the one designed by Stieger *et al.*,^35^ recognize the C-terminal domain of BSEP which is frequently targeted by alloimmune antibodies in AIBD^19^ , as observed in our study. Of note, the antibody used in our study recognizes the nucleotide-binding domain 1, which has not been reported to be targeted by anti-BSEP antibodies. In addition, higher titers of anti-BSEP antibodies might interfere with BSEP immunostaining, whereas low titers might not. In patient #1, the only patient for whom multiple histological data and anti-BSEP antibody titers were available during the same episode (Table S3), the decrease of BSEP immunostaining correlated with antibody titers, with a diffuse (normal) canalicular BSEP immunostaining once the titers fell below 1/10.

Treatments of AIBD used in our cohort combined steroids (oral or pulse), rituximab, plasmapheresis, IA and/or IVIg, similarly to those reported previously.^12^^,^^16^^,^^17^^,^[20], [21], [22], [23]^,^^25^^,^^26^ The aim of these strategies is to reduce the amount of circulating anti-BSEP antibodies and/or to deplete antibody-producing plasma cells. Given the central role of these antibodies in the pathogenicity of AIBD, achieving negative anti-BSEP antibodies in serum is likely to offer remission and prevent AIBD relapse. A recent meta-analysis showed that rituximab-based treatment improved AIBD in >80% of patients.^33^ Plasma cell depletion using bortezomib also proved efficient in recent case reports.^29^^,^^30^ Moreover, hematopoietic stem cell transplantation was able to suppress serum anti-BSEP antibody production in a patient with refractory AIBD.^24^ In our cohort, treatment with rituximab and/or plasmapheresis/IA was used as a first-line therapy in all seven patients with prospectively-identified AIBD. These treatments enabled a reduction or a negativation of serum anti-BSEP antibodies in all patients but one. Regarding relapse, most of the patients (five out of six patients) that did not receive a maintenance therapy based on rituximab or IVIg did experience a relapse of AIBD. By contrast, the use of such maintenance treatment in three patients enabled prolonged survival with negative or very low anti-BSEP antibody titers in all, although one patient relapsed at the time of complete interruption of the IS regimen. Similarly, one patient (patient #9) achieved initial AIBD remission with very low anti-BSEP antibody titer (1/1), but relapsed when receiving tacrolimus, oral steroids, and mycophenolate mofetil (MMF) maintenance therapy. After the second remission achieved using plasmapheresis, repeated rituximab administration enabled persistent remission with negative anti-BSEP antibodies in serum.

Our data confirm that immunological remission should be the main goal of the treatment, both to obtain AIBD remission and to prevent relapse. This likely requires specific maintenance therapies such as IVIg and/or rituximab. Our data suggest that B-cell counts <5% could be a therapeutic target to maintain low serum anti-BSEP antibody titers and prevent AIBD relapse. Therefore, B-cell counts, together with anti-BSEP antibodies, should be monitored closely in AIBD patients. A specific issue is the management of ‘refractory’ AIBD, as observed in patient #2 at diagnosis and in patient #1 at relapse, which did not respond to very intensive medical therapy and led to the death of the two patients. The use of new therapeutics developed for antibody-mediated rejection, such as the IgG-cleaving enzyme imlifidase,^36^ could enable a short antibody-free window, during which reLT could be performed with an expected better outcome. More efficient B-cell depletion using specific chimeric antigen receptor T cells, as recently reported in patients with IgG4-related disease, or new B cells depleting drugs such as inebilizumab, might also prove useful.^37^^,^^38^ Lastly, considering the high AIBD frequency (∼50%) observed in patients with severe *ABCB11* genotypes, another open question is the possibility to prevent its occurrence by preventive IS regimen. In two patients with either a mild (patient #11) or severe *ABCB11* genotype (patient #24), early MMF introduction and repeated post-LT IVIg administrations were used, without AIBD occurrence during a 4-year follow-up.

**Fig. 5:**
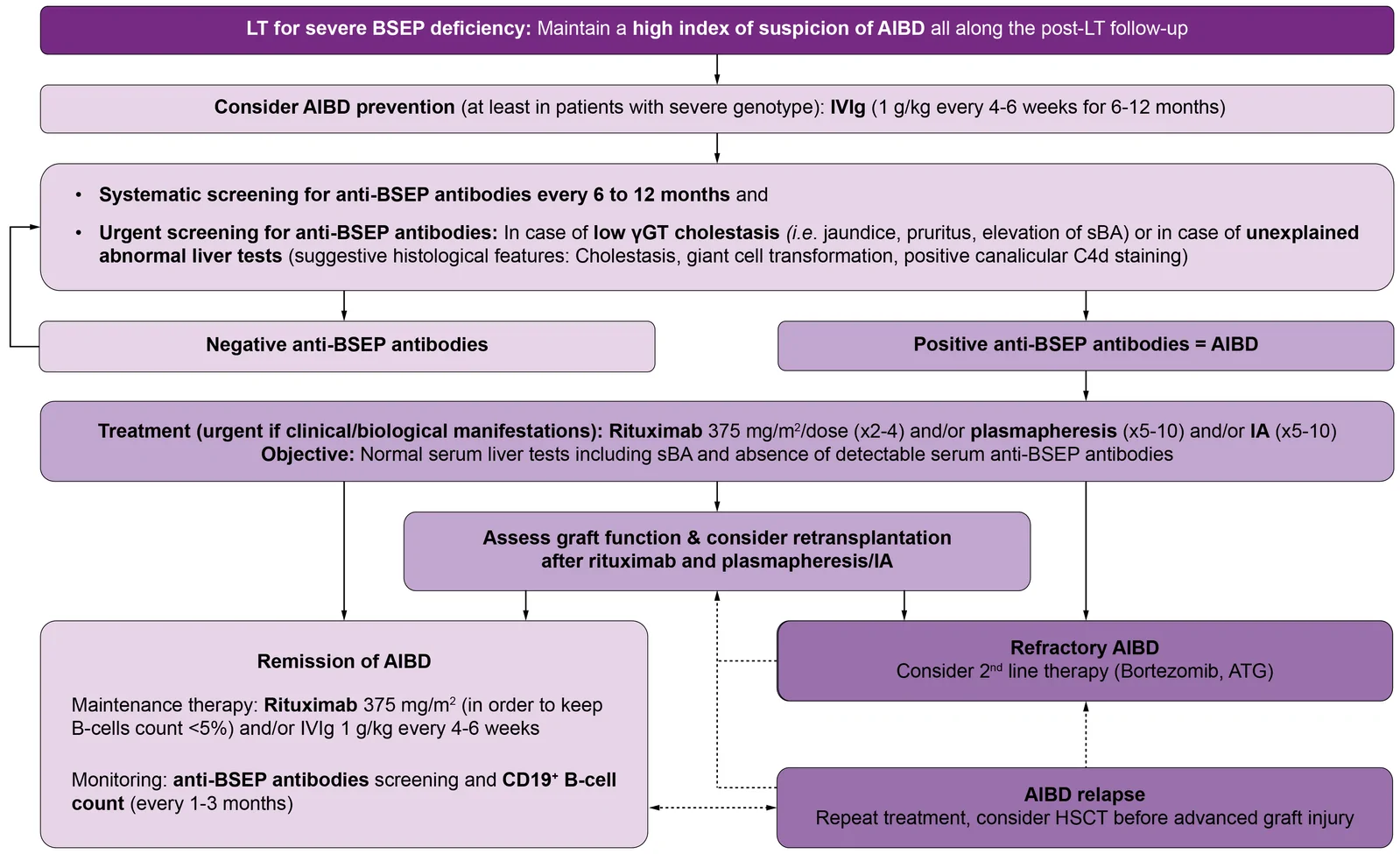
Proposed algorithm for the prevention, screening and management of AIBD, as performed in our department. This is based solely on expert opinion, considering our clinical practice and the results presented in this publication. AIBD, alloimmune anti-BSEP liver disease; ATG, anti-thymocyte globulins; BSEP, bile salt export pump; C4d, complement fragment 4d; HSCT, hematopoietic stem cell transplantation; IA, immunoadsorption; IVIg, intravenous immunoglobulins; LT, liver transplantation; sBA, serum bile acid levels.

Our study is the largest cohort reporting on risk factors, prevalence, management, and outcome of AIBD following LT in patients with severe BSEP deficiency to date. However, several limitations exist, mostly because of the retrospective nature of this work, such as missing data and heterogeneity in management over the years. Notably, the study’s duration of 25 years, with many patients receiving LT before awareness of AIBD, required the use of both prospectively and retrospectively collected sera. This long duration, together with the multicentric nature of the study, explained the wide heterogeneity in AIBD management and follow-up. These limitations highlight the need for AIBD diagnosis and management guidelines. Considering the rarity of severe BSEP deficiency, evidence to guide AIBD management are unlikely to be obtained through controlled clinical trials and will rather be based on lower-grade evidences. Standardization of AIBD diagnosis and treatment protocols is required to enable future international prospective studies. Based on our experience, we propose a specific prevention, screening, and management of AIBD in patients with severe BSEP deficiency (detailed in Fig. 5).

AIBD is a frequent complication of LT in patients with severe BSEP deficiency, especially in patients with severe *ABCB11* genotypes, this population of patients being the one typically requiring LT early in life. Screening for serum anti-BSEP antibodies should be routinely and regularly offered to these patients for early diagnosis and treatment of AIBD, given its frequency and severity. First-line therapy using rituximab and/or plasmapheresis/IA and maintenance therapy using rituximab and/or IVIg should be considered to obtain prolonged immunological remission and improve outcome.

## Abbreviations

Ab, anti-BSEP antibody titers; AIBD, alloimmune anti-BSEP liver disease; ALT, alanine aminotransferase level; AMR, antibody-mediated rejection; ATG, anti-thymocyte globulins; Aza, azathioprine; BA, biliary anastomosis; BSEP, bile salt export pump; C4d, complement component 4d; CCS, corticosteroids; CMV, cytomegalovirus; Cyclo, cyclosporine; DSA, donor-specific antibodies; HCC, hepatocellular carcinoma; HSCT, hematopoietic stem cell transplantation; IA, immunoadsorption; IS, immunosuppression; IVIg, intravenous immunoglobulins; LAFSc, liver allograft fibrosis score; LF, liver failure; LT, liver transplantation; MMF, mycophenolate mofetil; NA, not available; PFIC2, progressive familial intrahepatic cholestasis type 2; PP, plasmapheresis; reLT, liver retransplantation; RTX, rituximab; sBA, serum bile acids; Tacro, tacrolimus; TCMR, T-cell-mediated rejection, UDCA, ursodeoxycholic acid; γGT, gamma-glutamyl transferase.

## Authors’ contribution

Study design: AG, CR, GR, EG, EJ. Clinical data collection: AG, CR, GR, MA, TA, PB, DD, EDM, BH, BR, MR, XS, GM. Experimental data collection: ML, TF. Pathology data collection and review: AM, CM. Writing of original draft: AG, CR. Extensive review: EG, EJ. Review of the final manuscript and approval of the submitted version: all authors.

## Data availability

All data will be accessible upon request.

## Financial support

No financial support was received to produce this manuscript.

## Conflicts of interest

The authors declare no conflicts of interest that pertain to this work.

Please refer to the accompanying ICMJE disclosure forms for further details.
