# Supplementary material for: Post-transplant alloimmune liver disease in bile salt export pump deficiency: A multicenter study
Source: JHEP Rep. 2026 Jul 25;8(10):101969. doi: 10.1016/j.jhepr.2026.101969 (PMC13594885; doi:10.1016/j.jhepr.2026.101969)
Supplement: Supplementary file 1 [file mmc1.pdf]

Post-transplant alloimmune liver disease in bile salt export pump deficiency: A multicenter study

Antoine Gardin, Chiara Rubino, Alice Michaut, Géraldine Résin, Marion Almes, Teresa Antonini, Patrick Borentain, Dominique Debray, Eleonora De Martin, Bogdan Hermeziu, Bertrand Roquelaure, Mathias Ruiz, Xavier Stéphenne, Martine Lapalus, Giuseppe Maggiore, Thomas Falguières, Charlotte Mussini, Emmanuel Gonzales, Emmanuel Jacquemin

Table of contents

Supplementary Methods....3

Fig. S1....4

Fig. S2....5

Fig. S3....6

Fig. S4....7

Table S1....9

Table S2....11

Table S3....15

Table S4....18

Table S5....20

Supplementary Methods

Analysis of the epitope specificity of the anti-BSEP antibodies in patient serum.

In order to identify the antigenic specificity of anti-BSEP antibodies, an immunogenicity profile of human BSEP (NP_003733.2; 1321 amino-acids) was established by Genecust (Boynes, France). In silico analysis based on the secondary structure of BSEP and the hydrophobicity profile generated with HMMTop (http://www.enzim.hu/hmmtop/) highlighted three peptide sequences with potential interest in the N-terminal region (amino-acids 21-35), the first extracellular loop (amino-acids 115-129) and the C-terminal region of the protein (amino-acids 1297-1312). Thus, three soluble peptides overlapping these potentially immunogenic regions (shown in bold in the following sequences) were designed: (1) KKFGEENDGF**ESDKSYNNDKKSRLQ**DEKKGDGVRV (N-terminal or B0 peptide, amino-acids 11-45); (2) LIFGTMTDVFIDYDVELQEL QIPGKACVNNTIVW**TNSSLNQNMTNGTRC**GLLNIESEMIKFASYYAG (B1 peptide, amino-acids 81-147); (3) **VMAQGVVIEKGTHEEL MAQKGAYYK**LVTTGSPIS (C-terminal or B2 peptide, amino-acids 1288-1321). The three purified peptides were reconstituted at a concentration of 1 µM in pure water, aliquoted and stored at -80°C until further use for ELISA. BSEP soluble peptides (B0, B1 and B2) were diluted at 1 µg/mL in the isotonic working buffer (25 mM Tris-HCl pH 7.4, 137 mM NaCl, 5.2 mM KCl) and adsorbed in wells (100 ng per well) of high binding 96-well ELISA plates Microlon 600 (Greiner Bio-One). After overnight incubation at 4°C and washes with the same buffer, samples were saturated 2 hours at room temperature in Tris-buffered saline containing 0.1% Tween 20 and 0.5% bovine serum albumin (Sigma-Aldrich Chimie). Then serum samples were sequentially incubated with 1:200 to 1:6400 serial dilutions of AIBD or control (biliary atresia) patients' sera (45 min at room temperature) and alkaline phosphatase-coupled anti-human secondary antibodies (30 min at room temperature). Then, 50 µL of alkaline phosphatase yellow (p-nitrophenylphosphate) liquid substrate for ELISA (Sigma-Aldrich Chimie) was added in each well and the colorimetric reaction was stopped by the addition of 50 µL of 0.1 M EDTA. Absorbance at 405 nm in the linear range was read using a Wallac 1420 Victor2 microplate reader (Perkin Elmer).

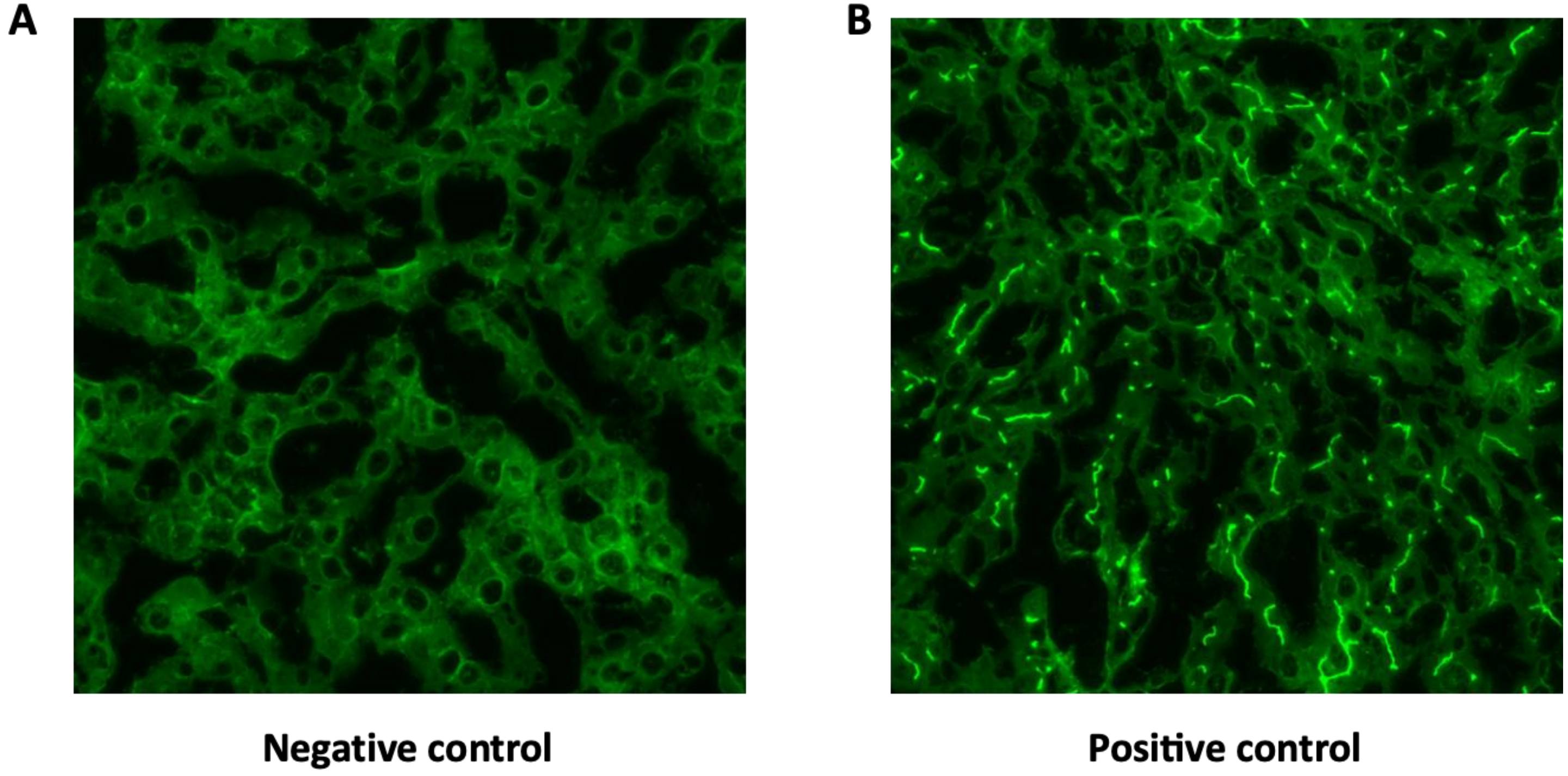

Fig. S1. Indirect immunofluorescence of cryosections of normal human liver incubated with patient serum samples to screen for anti-BSEP antibodies. A: negative control (magnification x400) showing the absence of immunoglobulin G deposit at the canalicular membrane of hepatocytes. B: positive control (magnification x400) showing immunoglobulin G deposits at the canalicular membrane of hepatocytes.

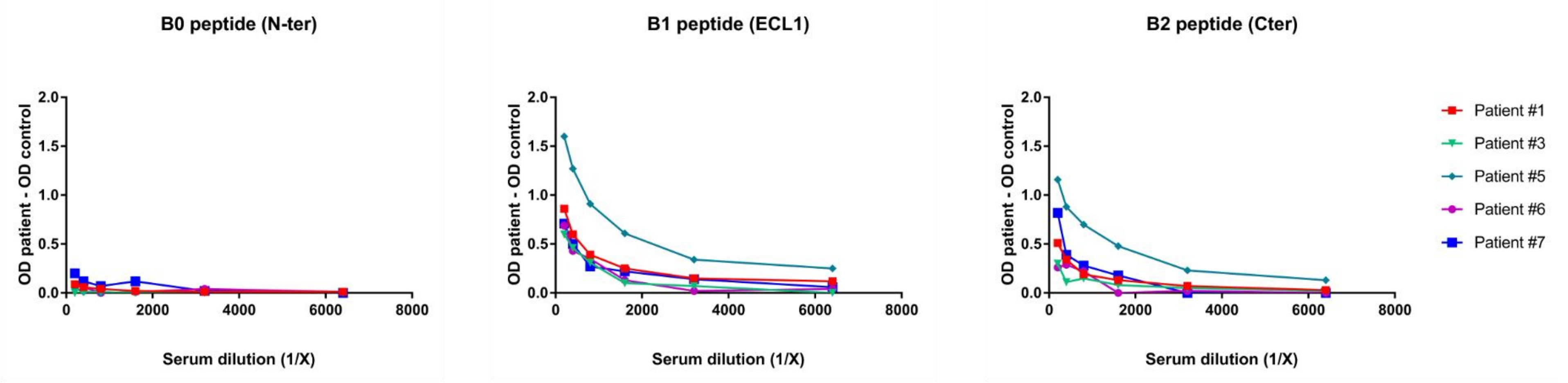

Fig. S2. **Analysis of the epitope specificity of the anti-BSEP antibody identified in serum of patients with AIBD**. ELISA performed using serum of patients with AIBD and three different peptides designed from the BSEP protein, expressed as the difference of optical density (OD) calculated between the tested (patient serum) and control (serum from biliary atresia patient) sera. The three peptides are named B0 (N-terminus), B1 (first extracellular loop/ECL1), B2 (C-terminus). Serial dilutions of the sera from 1:200 to 1:6400 were used.

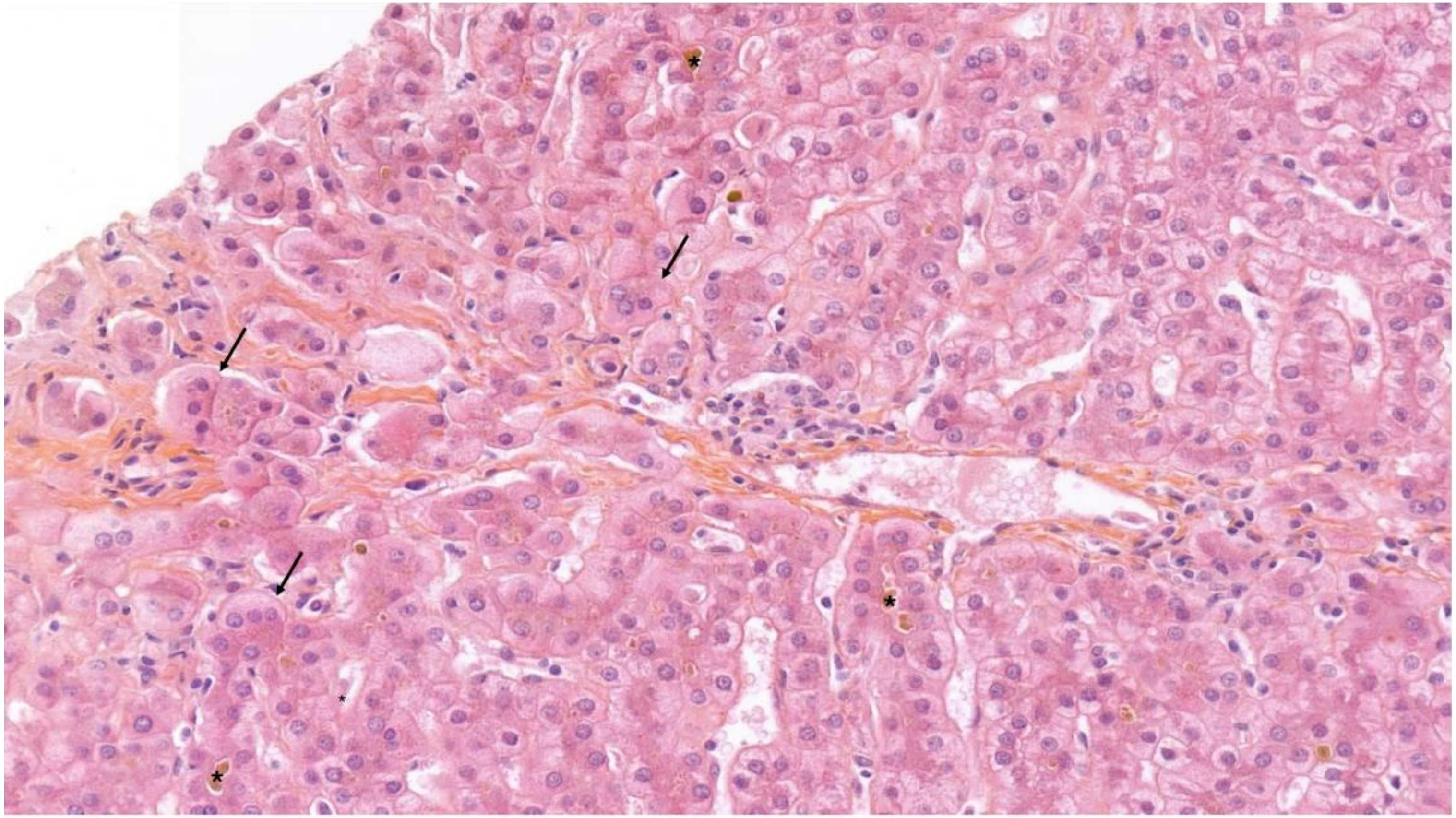

Fig. S3. Histological features of AIBD observed on liver biopsies. HES staining of biopsy of patient #1 (at relapse) recapitulating the cardinal features of AIBD, namely canalicular cholestasis (*) and giant transformation of hepatocytes (arrows). Magnification x 20.

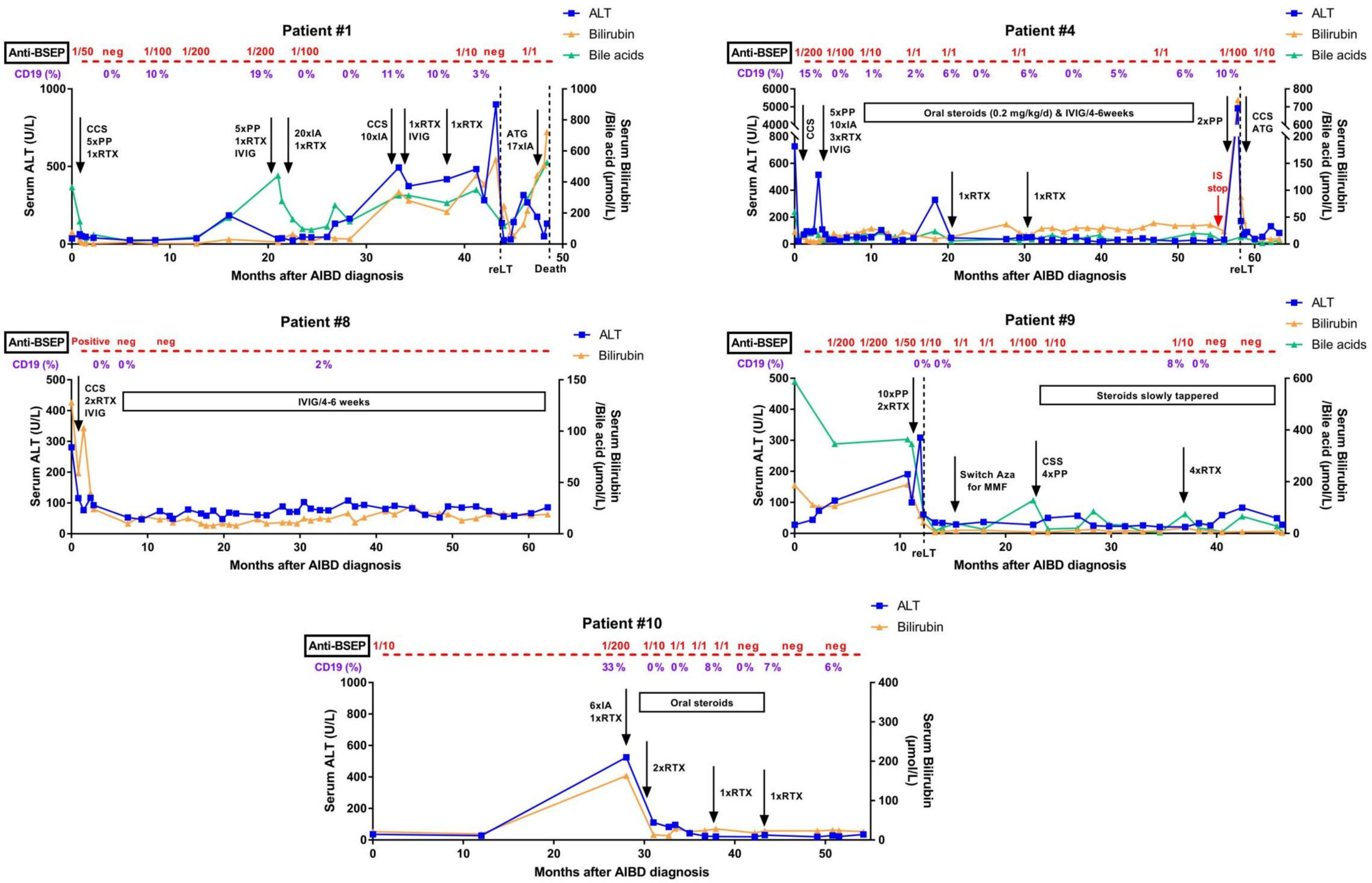

Fig. S4. Treatment and evolution of laboratory parameters and anti-BSEP antibody titers for patients #1, #4, #8, #9 and #10. Quantification of circulating CD19+ B-cells in plasma (% of total lymphocyte count) is shown. *ALT: alanine aminotransferase activity in serum; Anti-BSEP: anti-bile*

salt export pump antibody titers; ATG: anti-thymoglobulin; Aza: azathioprine; CCS: corticosteroids pulse; IA: immunoadsorption; IS: immunosuppressive regimen; IVIg: intravenous immunoglobulin; LT: liver transplantation; MMF: mycophenolate mofetil; neg: negative; PP: plasmapheresis; RTX: rituximab.

Table S1. Main characteristics of the 10 patients with severe BSEP deficiency who developed AIBD after liver transplantation.

Patient	Sex	*ABCB11* Variants	Age at LT in y (y of LT)	Indication for LT	Type of donor	BSEP staining on native liver	IS regimen	Complications after LT (interval after LT)	Treatment of complications
**#1**	M	c.3400 C>T (p.Gln1134*) homozygous	5 (2010)	Cirrhosis, LF	Deceased	Negative	Basiliximab, tacrolimus	TCMR (12d)	Steroids
								TCMR (0.5y)	Steroids, MMF
**#2**	M	c.1642_1644delTTT (p.Phe548del, in frame); c.3722del (p.Pro1091Leufs*6)	12 (2003)	Pruritus	Deceased	NA	NA	BA leakage (NA)	Surgery
								TCMR (NA)	Steroids
**#3[£]**	F	c.301delCA (p.Gln101Aspfs*8); c.2944G>A (p.Gly982Arg)	9 (1990)	Cholestasis and pruritus	Deceased	Negative	Cyclosporine, steroids	None	None
**#4**	F	c.1243C>T (p.Arg415*) homozygous	9.7 (2014)	Cirrhosis, Pruritus, dysplastic nodule	Deceased	Negative	Basiliximab, tacrolimus	BA stenosis (13d)	Surgery
								CMV infection (15d)	Ganciclovir
								TCMR (23d)	Steroids
								Plasma cell-rich rejection (1y)	Steroids, MMF
**#5[£]**	M	c.3268C>T (p.Arg1090*) homozygous	4.4 (1996)	Cirrhosis, LF	Living	Negative	Cyclosporine, azathioprine, steroids	TCMR (9d)	Steroids
								BA stenosis (14d)	Interventional radiology, Surgery
**#6**	M	Homozygous 17655bp deletion within *ABCB11*	3.9 (1998)	Cirrhosis, LF	Deceased	Negative	Cyclosporine, azathioprine, steroids	TCMR (7d)	Steroids
								Renal artery stenosis (0.3y)	Surgery
								TCMR (0.5y)	Steroids
								TCMR (2y)	Steroids
								Chronic rejection (3y)	Switch IS for tacrolimus and MMF

								Iterative rejection episodes (4-6y)	Tacrolimus dose increase; steroids; MMF interruption
								CMV hepatitis (5y)	Transient IS reduction
								BA stricture	UDCA
**#7£**	M	c.77-19T>A homozygous (p.Tyr26Ilsfs*7). Abnormal splicing.	2.8 (1989)	Cirrhosis, Pruritus	Deceased	Negative	Cyclosporine, steroids	TCMR (23d)	Steroids
								BA stenosis (30d)	Surgery
**#8**	M	c.1062T>A (p.Tyr354*) homozygous	2.3 (2012)	Cirrhosis, HCC	Deceased	Negative	Basiliximab, tacrolimus	TCMR (14d)	Steroids
								TCMR (30d)	Steroids, MMF
								BA stricture	Interventional radiology
**#9**	F	c.1101_1102delAG (p.Val368Argfs*27) ; del 2q24.3-q31.1 encompassing *ABCB11*	2.7 (2019)	Pruritus	Deceased	Negative	Basiliximab, tacrolimus	Plasma-cell rich rejection (0.3y)	Steroids, azathioprine
**#10**	M	c.1062T>A (p.Tyr354*) homozygous	5.7 (2008)	Cirrhosis, cholestasis	Deceased	NA	Basiliximab, tacrolimus, steroids	Infected ascites (30d)	Antibiotics, surgery

AIBD: alloimmune anti-BSEP liver disease; BA: biliary anastomosis; BSEP: bile salt export pump; CMV: cytomegalovirus; d: days; F: female; HCC: hepatocellular carcinoma; IS: immunosuppression; LF: liver failure; LT: liver transplantation; M: male; MMF: Mycophenolate mofetil; NA: not available; TCMR: T-cell mediated rejection; UDCA: ursodeoxycholic acid; y: year. [£]indicates retrospectively-diagnosed patients.

Table S2. Main characteristics of 25 patients with severe BSEP deficiency who did not develop AIBD.

Patient	Sex	*ABCB11* Variants	Age at LT in y (y of LT)	Indication for LT	Type of donor	BSEP staining on native liver	IS regimen	Complications (interval after LT)	Treatment of complications	Last follow-up (interval after LT)
**#11[#]**	M	c.1387A>C (p.Thr463Pro) homozygous	0.8 (2020)	LF, HCC	Deceased	Negative	Basiliximab, tacrolimus, MMF IVIg for 6 months post-LT	TCMR (10d)<br>BA stricture (30d)	Steroids<br>UDCA, Interventional radiology, surgery	Alive (4.2y)
**#12[#]**	F	c.3628A>C (p.Thr1210Pro) homozygous	16 (2016)	Pruritus	Deceased	Negative	Basiliximab, tacrolimus	TCMR & Plasma-cell rich rejection (4.5y)	Steroids MMF initiation	Alive (7.2y)
**#13**	F	c.2300C>G (p.Thr767Arg) homozygous	6.9 (2016)	Cirrhosis, HPS, Pruritus	Deceased	Positive	Basiliximab, tacrolimus, steroids, MMF	Bleeding (1d) & Infection (3-20d)<br>Ischemic cholangiopathy (30d)	Multiple surgery<br>UDCA	Alive (7.1y)
**#14**	F	c.1308+2T>A; c.1408C>T (p.Arg470*)	2.0 (2009)	Cirrhosis, LF, HCC	Deceased	Negative	Basiliximab, tacrolimus	TCMR (12d)<br>EBV-related tonsillar PTLD (0.3y)<br>HCC recurrence (13y)	Steroids<br>Rituximab, tonsillectomy<br>Surgery Chemotherapy	Death (14y) HCC recurrence
**#15**	M	c.1062T>A (p.Tyr354*) ; c.2944G>A (p.Gly982Arg)	4 (2008)	Cirrhosis, LF, HCC	Deceased	Negative	Basiliximab, tacrolimus	TCMR (19d)<br>Necrosis of segment IV<br>Inferior vena cava stenosis (45d)	Steroids<br>Surgery<br>Interventional radiology	Alive (14.7y)
**#16***	F	c.3181_3184delATCA (p.Ile1061ValfsTer35); c.1259T>C p.(Ile420Thr)	12.4 (2015)	Pruritus	Deceased	Negative	Basiliximab, tacrolimus	TCMR (13d)	Steroids	Alive (7.9y)
**#17***	M	c.3181_3184delATCA (p.Ile1061ValfsTer35); c.1259T>C (p.Ile420Thr)	0.4 (1993)	Cirrhosis, LF	Deceased	Negative	Cyclosporine, azathioprine, steroids	TCMR (6d)<br>TCMR (30d)<br>Chronic Hepatitis B infection (0.7y)<br>EBV-related PTLD	Steroids<br>Steroids<br>Anti-viral treatment 	Alive (24.4y)

**#18**	M	c.890A>G (p.Glu297Gly); c.1826_1827insCA (p.Ile610Glyfs*45)	7.7 (2002)	Pruritus	Deceased	Negative	Cyclosporine, azathioprine, steroids	TCMR (10y)	Switch from azathioprine to MMF	Alive (22.2y)
**#19**	F	c.1062 T>A (p.Tyr354*) homozygous	3.5 (2017)	Pruritus	Deceased	Negative	Basiliximab, tacrolimus, MMF, steroids	EBV-related PTLD	Rituximab	Alive (6.2y)
**#20**	F	c.3432C>A (p.Ser1144Arg); c.611+4A>G	8.5 (1990)	Cirrhosis, LF	Deceased	Negative	Cyclosporine, azathioprine, steroids	BA stenosis (5d)	UDCA, Interventional radiology	Alive (33.7y) Pregnancy
**#21**	M	c.3691 C>T (p.Arg1231Trp); c.1582delA (p.Ils528*)	1.7 (2007)	Cirrhosis	Deceased	NA	Basiliximab, tacrolimus	TCMR (60d)	Steroids	Alive (15.3y)
								EBV-related PTLD (2y)	Tonsillectomy, Rituximab	
								TCMR (12y)	Steroids	
								TCMR (15y)	Steroids, tacrolimus dose increase	
**#22**	F	c.890 A>G (p.Glu297Gly); c.1435-17_1450dup (p.His484Argfs*5)	9.4 (2002)	Cirrhosis; Pruritus	Deceased	Negative	Tacrolimus, steroids	BA stenosis	UDCA, Interventional radiology	Alive (22.1y) Pregnancy
								Hepatic artery stenosis	Surveillance	
**#23**	M	c.3213 +4 A>G; c.3457 C>T (p.Arg1153Cys)	12.4 (2008)	Cirrhosis; LF, HCC	Deceased	Negative	Basiliximab, tacrolimus	BA leakage (1d)	Surgery	Alive (15.4y)
								TCMR (10d)	Steroids	
**#24**	M	c.390-1G>A; c.3213+5G>A	8.2 (2020)	Pruritus	Living	Negative	Basiliximab, tacrolimus, MMF, IVI for 6 months post-LT	Mild Budd-Chiari syndrome	Heparin	Alive (4.3y)
**#25**	M	c.545T>A (p.Ile182Lys); c.3213+5G>A	6.3 (2000)	Cirrhosis	Deceased	Negative	Tacrolimus, steroids	TCMR (2d)	Steroids	Alive (14y)
								Digestive Burkitt lymphoma (8y)	Transient IS withdrawal Chemotherapy	
								BA stricture (11y)	UDCA, Surgery	
**#26**	M	c.3123 T>A (p.Tyr1041*); c.3008G>A (p.Gly1003Glu)	14.6 (2009)	Pruritus	Deceased	Negative	Basiliximab, tacrolimus	Hepatic artery stenosis	Spontaneous resolution	Alive (12.6y)

**#27**[$]	M	c.1826_1827dupCA (p.Ile610Glnfs*45) homozygous	12 (2016)	Cirrhosis, HPS, Pruritus	Deceased	NA	Basiliximab, tacrolimus, MMF, steroids	Bleeding & BA leak (2d)	Surgery	Alive (6.1y)
								TCMR (4y & 5y)	Steroids	
**#28**[$]	F	c.1826_1827dupCA (p.Ile610Glnfs*45) homozygous	6.0 (2016)	Cirrhosis	Deceased	Negative	Basiliximab, tacrolimus, MMF, steroids	TCMR (0.3y & 1y)	Steroids	Alive (6.1y)
								TCMR (4y)	MMF dose increase	
								TCMR (5y)	Tacrolimus dose increase	
								BA stricture	UDCA	
**#29**[£]	F	c.3382C>T (p.Arg1128Cys) homozygous	7.5 (2001)	Cirrhosis, LF	Deceased	Negative	Tacrolimus, steroids	TCMR (14d)	Steroids	Alive (22.8y) 2 pregnancies
								TCMR (60d)	Steroids, azathioprine	
**#30**[£]	F	c.3382C>T (p.Arg1128Cys) homozygous	8.0 (2008)	Cirrhosis, LF	Deceased	Negative	Basiliximab, tacrolimus	TCMR (0.3y)	Steroids	**reLT** (9.4y)<br><br>**Alive** (15.6y, 6.2y post reLT) Pregnancy
								TCMR (4y)	Steroids, MMF	
								BA strictures	UDCA, Interventional radiology	
								Chronic rejection (6y)	reLT	
**#31**	F	c.713G>T (p.Gly238Val) homozygous	2.6 (1994)	LF	Deceased	Negative	Cyclosporine, steroids, Azathioprine	TCMR (7d)	Steroids, azathioprine	**Listed for reLT** (persistent portal hypertension)<br><br>Alive (15.9y)
								Pneumocystis infection		
								Chronic rejection (10y)	Switch Azathioprine for MMF	
								Portal vein thrombosis	Meso-Rex bypass	
**#32**	F	c.1826_1827dupCA (p.Ile610Glnfs*45) homozygous	2.8 (2014)	Pruritus	Living	NA	Basiliximab, cyclosporine	None	None	Alive (9.3y)
**#33**	F	c.3400C>T (p.Gln1134*) homozygous	4.0 (2012)	Pruritus, HCC	Deceased	NA	Basiliximab, tacrolimus	Budd-Chiari (5y)	Interventional radiology	Alive (11.1y)
								Chronic rejection (10y)	Azathioprine	
**#34**[#]	M	c.409G>A (p.Glu137Lys); c.1418_1419ins18pb	7 (2021)	Pruritus	Living	Focally positive	Basiliximab, tacrolimus	BA stricture	UDCA	Alive (2y)
**#35**[#]	F	c.677C>T (p.Ser226Leu) homozygous	17.3 (2023)	Cirrhosis, LF	Deceased	Negative	Tacrolimus, MMF, Steroids	TCMR (4d)	Steroids	Alive (1.1y)
								BA leak (4d)	Surgery	

*AIBD: alloimmune anti-BSEP liver disease; BSEP: bile salt export pump; d: days; EBV: Epstein Barr Virus; F: female; IS: immunosuppression; HPS: hepatopulmonary syndrome; IVIg: intravenous immunoglobulin; LF: liver failure ; LT: liver transplantation; M: male; MMF: Mycophenolate mofetil; NA: not available; PTLD: post-transplant lymphoproliferative disorder; reLT: liver retransplantation; TCMR: T-cell mediated rejection; UDCA: ursodeoxycholic acid; y: year. *, $, £ denotes siblings. #All these four patients received 4-phenylbutyrate pre-LT as targeted pharmacotherapy.*

Table S3. Liver biopsy in the follow-up of patients with AIBD.

Patient	Time of biopsy (time after AIBD diagnosis)	Histology	Fibrosis LAFSc score	Acute TCMR (BANFF)	BSEP immunostaining	C4d immunostaining BANFF score	AMR
#1	**Initial episode**	Severe canalicular cholestasis; rare giant hepatocytes<br>Mild centrilobular and portal inflammation; biliary obstruction	4 (2+0+2)	No	**Reduced canalicular expression**	**Positive (canalicular, lateral membrane)**<br>BANFF: 0	No
	**Relapse** (1.7y)	Canalicular cholestasis; increase of portal inflammation, persistence of biliary obstruction; hepatocyte clarification	5 (2+1+2)	No	**Reduced canalicular expression**	**Positive (canalicular)**<br>BANFF: 0	No
	**Relapse** (1.9y)	Canalicular cholestasis; several giant hepatocytes; mild centrilobular and portal inflammation	3 (2+0+1)	No	**Reduced canalicular expression**	**Positive (canalicular)**<br>BANFF: 0	No
	**Relapse** (2.7y)	Cholestatic cirrhosis; several giant hepatocytes with focal ballooning; moderate centrilobular and portal inflammation; biliary obstructions	9 (3+3+3)	No	**Reduced canalicular expression**	**Positive (canalicular, lateral membrane)**<br>BANFF: 0	No
	**Explanted graft** (3.6y)	Cholestatic cirrhosis; several giant hepatocytes with focal ballooning; moderate centrilobular and portal inflammation; biliary obstruction	9 (3+3+3)	No	Normal canalicular expression	**Positive (canalicular)**<br>BANFF: 0	No
	**Relapse**<br>(0.1y after reLT)	Moderate canalicular cholestasis; rare giant hepatocytes; mild portal inflammation; biliary obstructions; rare ballooned hepatocytes	3 (1+1+1)	Yes<br>(4: 1+1+2)	**Reduced canalicular expression**	**Positive (canalicular)**<br>BANFF: 0	No
	**Relapse**<br>(0.2y after reLT)	Increase of portal fibrosis; neutrophil inflammation and intraductular bile plugs (sepsis with possible biliary obstruction)	3 (2+0+1)	Yes<br>(7: 2+3+2)	Normal canalicular expression	**Positive (canalicular)**<br>BANFF: 0	No
	**Relapse**<br>(0.3y after reLT)	Increase of fibrosis; canalicular cholestasis with several giant hepatocytes; lobular hepatitis	6 (2+2+2)	Yes<br>(4: 1+2+1)	Normal canalicular expression	**Positive (canalicular)**<br>BANFF: 0	No
#2	**Initial episode**<br>*Explanted liver*[§]	Severe cholestasis, giant hepatocytes, moderate portal inflammation	7 (2+2+3)	No	Normal canalicular expression	NA	NA
	**Initial episode**[§]<br>(0.5y after reLT)	Cholestasis; giant hepatocytes; ductopenia (chronic TCMR)	2 (0+1+1)	No	Normal canalicular expression	NA	No
	**Initial episode**<br>(0.7y after reLT)	Cirrhosis; cholestasis; giant hepatocytes; ductopenia	9 (3+3+3)	NA	Normal canalicular expression	**Positive (canalicular)**	NA

**#3**	**Relapse**§ (14y)	Lobular cholestasis; giant hepatocytes; no biliary obstruction	NA	No	NA	NA	NA
**#4**	**Initial episode**	Lobular and portal hepatitis; rare giant hepatocytes	2 (1+1+0)	Yes (6: 3+1+2)	Normal canalicular expression	**Positive (canalicular)** BANFF: 0	No
	**Initial episode** (0.4y)	No cholestasis, some giant hepatocytes, Reduction of portal inflammation; increase in centrilobular inflammation	5 (1+2+2)	Yes (4: 1+0+3)	Normal canalicular expression	Positive (portal vein) BANFF score: 1+	No
	**Relapse** (4.8y) *Explanted liver*	Bridging hemorrhagic necrosis, canalicular cholestasis, giant hepatocytes, moderate portal inflammation	NA	No	Normal canalicular expression	**Positive (canalicular)** BANFF: 0	No
**#5**	**Initial episode**	Moderate portal and mild perivascular inflammation	2 (1+0+1)	No	Normal canalicular expression	NA	Yes (chronic)
	**Relapse** (10y) *Explanted liver*§	Biliary cirrhosis, chronic cholangitis, hepatocellular cholestasis with giant hepatocytes	NA	No	NA	NA	No
	**Protocol biopsy**§ (15y, 5y after reLT)	Mild lobular hepatitis with centrilobular necrosis No cholestasis. No giant hepatocytes.	NA	No	NA	NA	No
**#6**	**Initial episode**	Canalicular cholestasis; giant hepatocytes Moderate portal inflammation with ductular proliferation.	5 (3+2+0)	Yes (3: 2+0+1)	**Reduced canalicular expression**	Negative	No
	**Initial episode** (0.4y)	Canalicular cholestasis, giant hepatocytes. Moderate portal inflammation with ductular reaction	5 (3+2+0)	Yes (4: 2+1+1)	**Reduced canalicular expression**	Negative	No
	**Initial episode** (0.5y) *Explanted liver*	Cholestatic cirrhosis; several giant hepatocytes with lobular/bridging hepatocellular necrosis; biliary obstruction	9 (3+3+3)	No	Normal canalicular expression	Negative	No
	**Initial episode** (0.8y, 0.4y after reLT)	Mild cholestasis; focal lobular and portal hepatitis	3 (1+1+1)	No	Normal canalicular expression	Negative	No
	**Relapse** (9.7y)	Canalicular cholestasis; several giant hepatocytes	NA	NA	NA	NA	
**#7**	**Initial episode**§	Canalicular cholestasis, giant hepatocytes Slight centrilobular fibrosis	NA	NA	NA	NA	NA
**#8**	**Initial episode**	Canalicular cholestasis, giant hepatocytes Portal inflammation	3 (1+2+0)	Yes (5: 2+2+1)	**Reduced canalicular expression**	**Positive (canalicular)** BANFF: 0	No

**#9**	**Initial episode**[§]	Cholestasis with ductopenia and bile duct dystrophy	NA	NA	NA	NA	NA
	**Initial episode**[§] (1.2y, 0.1y after reLT)	Lymphocytic and eosinophilic inflammation; canalicular cholestasis	NA	Yes (5: 2+2+1)	NA	NA	NA
	**Initial episode**[§] (1.3y, 0.2y after reLT)	Mild portal fibrosis	4 (3+0+1)	NA	Normal canalicular expression	NA	NA
	**Relapse**[§] (1.9y, 0.8y after reLT)	Mild portal and lobular hepatitis	2 (1+1+0)	NA	NA	NA	NA
**#10**	**Initial episode** (at symptom onset)	Cholestasis; giant hepatocytes	NA	No	**Reduced canalicular expression**	**Positive (canalicular)**	No

LAFSc is expressed as the sum of a 0-3 score referring to portal tract, sinusoids and central vein fibrosis (total score: 0-9)[35]. TCMR was graded using BANFF score, expressed as the sum of a 0-3 score referring to portal tract inflammation, bile duct inflammatory lesions and venous endothelial inflammation (max 0-9)[36]. C4d staining was also staged according to the BANFF scoring system[36]. All biopsies were reviewed by a single pathologist of Bicêtre Hospital, unless indicated by the symbol "§". *AIBD: alloimmune anti-BSEP liver disease; AMR: antibody-mediated rejection; BSEP: bile salt export pump; C4d: complement component 4d; LAFSc: Liver allograft fibrosis score; LT: liver transplantation; NA: not available; reLT: liver retransplantation; TCMR: T cell–mediated rejection; y: year.*

Table S4. Histology analysis of liver biopsies performed in 11 patients without AIBD.

Patient	Interval after LT *(years)*	Histology	LAFSc	TCMR	BSEP immunostaining	C4d immunostaining BANFF score	AMR
**#11**	0.9y	No cholestasis, no giant hepatocytes. Biliary obstruction.	2 (1+0+1)	No	Normal canalicular expression	Negative	No
	2y	No cholestasis, no giant hepatocytes. Biliary obstruction.	4 (2+1+1)	No	Normal canalicular expression	Negative	No
	3.8y	No cholestasis, no giant hepatocytes. Biliary obstruction.	5 (3+1+1)	No	Normal canalicular expression	Negative	No
**#14**	10y	No cholestasis, no giant hepatocytes Portal fibrosis, biliary obstruction	4 (2+1+1)	No	**Reduced canalicular expression**	Negative	No
**#15**	10y	No cholestasis, no giant hepatocytes Chronic hepatitis, acute AMR	5 (3+1+1)	Undetermined TCMR	Normal canalicular expression	Positive (sinusoidal) BANFF: 1 **No canalicular staining**	Yes (acute)
**#16**	0.1y	No cholestasis, no biliary obstruction Acute TCMR	1 (0+0+1)	Yes (5: 2+1+2)	Normal canalicular expression	Negative	No
**#17**	8y	No cholestasis, no giant hepatocytes Cirrhosis with chronic hepatitis B	8 (3+3+2)	No	Normal canalicular expression	Negative	No
**#18**	16y	No cholestasis, no giant hepatocytes Central vein fibrosis (chronic AMR)	5 (2+2+1)	Yes (3)	Normal canalicular expression	Negative	Yes (chronic)
	20y	No cholestasis, no giant hepatocytes Central vein fibrosis	4 (1+1+2)	No	Normal canalicular expression	Negative	No
**#20**	10y	Normal morphology	1 (1+0+0)	No	Normal canalicular expression	Negative	No
**#23**	10y	No cholestasis, no giant hepatocytes Biliary obstruction	6 (3+2+1)	No	Normal canalicular expression	Negative	No
**#24**	0.1y	Mild cholestasis, rare giant hepatocytes Lobular hepatitis	1 (0+0+1)	Yes (2: 1+0+1)	Normal canalicular expression	Negative	No
**#30**	6.8y	Mild central vein fibrosis No cholestasis or giant hepatocytes.	1 (0+0+1)	No	Normal canalicular expression	Negative	No
	9.5y	**Explanted liver**: chronic TCMR, cirrhosis, intense cholestasis	9 (3+3+3)	No	Normal canalicular expression	Negative	No
	11.3y (1.9y after reLT)	Chronic TCMR. No cholestasis, no giant hepatocytes	2 (1+0+0)	Yes (3: 1+1+1)	Normal canalicular expression	Positive (sinusoidal) BANFF: 0 **No canalicular staining**	No
	15y (5.5y after reLT)	Cirrhosis, chronic TCMR No cholestasis	NA	Yes (3: 1+1+1)	Normal canalicular expression	Negative	No
**#31**	10y	No cholestasis, no giant hepatocytes Central vein fibrosis	4 (2+1+1)	No	Normal canalicular expression	Negative	Undetermined acute AMR

LAFSc is expressed as the sum of a 0-3 score referring to portal tract, sinusoids and central vein fibrosis (total score: 0-9)[35]. TCMR was graded using BANFF score, expressed as the sum of a 0-3 score referring to portal tract inflammation, bile duct inflammatory lesions and venous endothelial inflammation (max 0-9)[36]. C4d staining was also staged according to the BANFF scoring system[36]. All biopsies were reviewed by a single pathologist of Bicêtre Hospital, unless indicated by the symbol "§". *AIBD: alloimmune anti-BSEP liver disease; AMR: antibody-mediated rejection; BSEP: bile salt export pump; C4d: complement component 4d; LAFSc: Liver allograft fibrosis score; LT: liver transplantation; NA: not available; reLT: liver retransplantation; TCMR: T cell–mediated rejection; y: year.*

Table S5. Detailed outcome of patients with AIBD

Patient	Initial episodes and relapses (time after AIBD diagnosis)	AIBD treatment		Outcome		Last follow-up (time after AIBD diagnosis)
		**Intensification therapy**	**Maintenance therapy**	**Clinical outcome**	**Immunological outcome**	
#1	**Initial episode**	Pulse steroids<br>Rituximab x 1<br>Plasmapheresis x 5<br>IVIg x 4	Tacrolimus - MMF	Clinical remission	Negative anti-BSEP Ab	**Death** (4y)<br>Recurrence of AIBD
	**Relapse (1.7y)**<br>*Pruritus*<br>*Anti-BSEP Ab 1/200*	Pulse steroids<br>Plasmapheresis x 5<br>Rituximab x 4<br>IA x 47<br>IVIg<br>Anti-thymocyte globulin	-	**Persistent cholestasis**<br>Liver failure with **reLT**<br>(3.6y after AIBD diagnosis)	Lowering of anti-BSEP Ab titer from 1/200 to 1/1	
#2	**Initial episode**<br>(diagnosis 1y after reLT)	MMF<br>Plasmapheresis<br>Rituximab<br>IVIg	-	**Persistent cholestasis**<br>Liver failure	Persistent anti-BSEP Ab<br>(no titer available)	**Death** (0.6y)<br>Recurrence of AIBD
#3*	**Initial episode**	Cyclosporine and steroid dose increase	Cyclosporine<br>Steroid	Clinical remission	NA	**Death** (17y) while waiting for reLT
	**Relapse (14y)**<br>*Jaundice*<br>*Positive anti-BSEP Ab*	Cyclosporine and steroid dose increase<br>IVIg	-	**Persistent cholestasis**<br>Evolution toward cirrhosis	Persistent anti-BSEP Ab<br>(no titer available)	
#4	**Initial episode**	Pulse steroids<br>IVIg x 10<br>Rituximab x 3<br>Plasmapheresis x 5<br>IA x 10	Tacrolimus – MMF<br>Steroids 0.2 mg/kg/d<br>IVIg/months<br>+/- Rituximab	Clinical remission	Lowering of anti-BSEP Ab titer from 1/200 to 1/1	**Alive** (5.2y)<br>Clinical remission<br>0.4y after relapse
	**Relapse (4.8y)**<br>*IS interruption*<br>*Jaundice, liver failure*<br>*Anti-BSEP Ab 1/100*	Pulse steroids<br>Plasmapheresis<br>Anti-thymocyte globulin	Tacrolimus – MMF<br>IVIg/months<br>+/- Rituximab	**reLT**<br>Clinical remission thereafter	Lowering of anti-BSEP Ab titer from 1/100 to 1/10	

#5*	**Initial episode**	Switch to tacrolimus<br>Steroid dose increase	Tacrolimus	Clinical remission	Positive anti-BSEP Ab at similar titer (1/1)	**Alive** (25.2y)<br>Clinical remission<br>15y after relapse
	**Relapse (7.1y)**<br>*Pruritus, elevated liver enzymes*<br>*Anti-BSEP Ab 1/50*	Tacrolimus dose increase<br>Addition of MMF	Tacrolimus - MMF	BA stenosis with **reLT**<br>(10y after AIBD diagnosis)<br>Clinical remission thereafter	Lowering of anti-BSEP Ab titer from 1/50 to 1/1	
#6	**Initial episode**	Pulse steroids<br>IVIg<br>Plasmapheresis x 12	Tacrolimus - MMF<br>Steroids	Liver failure with **reLT**<br>(0.5y after AIBD diagnosis)<br>Clinical remission thereafter	Negative anti-BSEP Ab	**Alive** (13y)<br>Clinical remission<br>3.5y after relapse
	**Relapse (9.7y)**<br>*Pruritus, jaundice*<br>*Anti-BSEP Ab 1/100*	Pulse steroids<br>IVIg x 2<br>Plasmapheresis x 6<br>Rituximab x 1	Tacrolimus<br>MMF<br>Steroids (0.2 mg/kg/d)	Clinical remission	Lowering of anti-BSEP titer from 1/100 to 1/1	
#7*	**Initial episode**	Cyclosporine and steroid dose increase<br>Azathioprine initiation	Cyclosporine<br>Azathioprine	Clinical remission	Positive anti-BSEP Ab at similar titer (1/10)	**Alive** (27y)<br>Clinical remission
#8	**Initial episode**	Pulse steroids<br>Rituximab x 2<br>IVIg x 1	Tacrolimus - MMF<br>IVIg/months	Clinical remission<br>(elevation of GGT and ALT due to chronic rejection)	Negative anti-BSEP Ab	**Alive** (5y)<br>Clinical remission (no pruritus/jaundice)
#9	**Initial episode**	Plasmapheresis x10<br>Rituximab x 2	Tacrolimus - MMF<br>Steroids	**reLT** for liver failure<br>(1.1y after AIBD diagnosis)<br>Clinical remission thereafter	Lowering of anti-BSEP Ab titer from 1/200 to 1/1	**Alive** (3.8y)<br>Clinical remission<br>1.9y after relapse<br>(Absence of pruritus /jaundice)
	**Relapse (1.9y)**<br>*Pruritus, elevated sBA*<br>*Anti-BSEP Ab 1/100*	Steroid pulse<br>Plasmapheresis x 4	Tacrolimus – MMF<br>Steroids<br>Rituximab	Clinical remission<br>(elevation of GGT and ALT due to biliary obstruction)	Negative anti-BSEP Ab	
#10	**Initial episode**	Steroids, Azathioprine<br>Rituximab x 3<br>IA x 6	Tacrolimus<br>Azathioprine<br>Steroids<br>Rituximab	Clinical remission	Negative anti-BSEP Ab	**Alive** (2.1y)<br>Clinical remission

Immunological remission was defined as negative anti-BSEP serum testing. Clinical remission was defined as resolution of clinical manifestations and normalization of serum liver tests, including sBA, except if abnormalities could be explained by another complication of LT (biliary obstruction, chronic rejection). *These patients were diagnosed retrospectively. *Ab: antibodies; AIBD: alloimmune anti-BSEP liver disease; ALT: alanine*

aminotransferase; BA: biliary anastomosis; BSEP: Bile salt export pump; GGT: gamma-glutamyltransferase; IA: immunoadsorption; IVIg: intravenous immunoglobulins; LT: liver transplantation; NA: not assessed; reLT: liver retransplantation; sBA: serum bile acids; y: year
