# Supplementary material for: Post-transplant alloimmune liver disease in bile salt export pump deficiency: A multicenter study
Source: JHEP Rep. 2026 Jul 25;8(10):101969. doi: 10.1016/j.jhepr.2026.101969 (PMC13594885; doi:10.1016/j.jhepr.2026.101969)
Supplement: Supplementary file 3 [file mmc4.pdf]

Post-transplant alloimmune liver disease in bile salt export pump deficiency: A multicenter study

Antoine Gardin[1,2,†], **Chiara Rubino**[3,†], Alice Michaut[4], Géraldine Résin[1], Marion Almes[1,5], Teresa Antonini[6], Patrick Borentain[7], Dominique Debray[8], Eleonora De Martin[5,9], Bogdan Hermeziu[1,5], Bertrand Roquelaure[10], Mathias Ruiz[11], Xavier Stéphenne[12], Martine Lapalus[5], Giuseppe Maggiore[13], Thomas Falguières[5], Charlotte Mussini[4], Emmanuel Gonzales[1,5], Emmanuel Jacquemin[1,5,*]

JHEP Reports **2026**. vol. 8 | 1–11

Check for updates

Abstract

Background & Aims: Patients with severe bile salt export pump (BSEP) deficiency may develop alloimmune anti-BSEP liver disease (AIBD) after liver transplantation (LT) as a result of anti-BSEP antibodies, but its frequency and risk factors are not precisely known.

Methods: A multicenter cohort of 35 patients who underwent LT for severe BSEP deficiency was screened retrospectively or prospectively after 2010 for anti-BSEP antibodies. Risk factors, management and outcome of AIBD were analyzed.

Results: Ten patients (29%) were screened positive for anti-BSEP antibodies and all developed AIBD, in median 5 years (range: 1.5–14 years) after LT. Clinical remission of AIBD was achieved in nine patients using various immunosuppressive therapies, with decreased or negative anti-BSEP antibody titers in six patients, all having received rituximab or plasmapheresis/immunoadsorption. Relapse occurred in six of nine patients, although maintenance therapy using rituximab and/or immunoglobulins enabled prolonged relapse-free survival. Because of AIBD, six patients (60%) required liver retransplantation either at initial AIBD episode or at relapse, and three patients (30%) died. Among the 25 patients without AIBD, one died (4%) and another one was retransplanted (4%). Although a severe *ABCB11* genotype (biallelic protein-truncating variants) was significantly associated with AIBD (80% in patients with AIBD *vs.* 28% in patients without AIBD, $p = 0.008$), negative BSEP immunostaining on native liver, post-LT CMV infection, presence of biliary anastomosis strictures or graft rejection were not.

Conclusions: In patients with severe BSEP deficiency, AIBD is a frequent post-LT complication that should be screened for, especially in patients with severe *ABCB11* genotypes. Early diagnosis and the use of rituximab and plasmapheresis/immunoadsorption may improve patient outcome.

© 2026 The Authors. Published by Elsevier B.V. on behalf of European Association for the Study of the Liver (EASL). This is an open access article under the CC BY license (http://creativecommons.org/licenses/by/4.0/).

Introduction

Severe bile salt export pump (BSEP) deficiency, also known as progressive familial intrahepatic cholestasis type 2 (PFIC2), is an autosomal recessive genetic disease caused by biallelic variants in the *ABCB11* gene, which encodes BSEP.[1,2] BSEP is a transporter exclusively expressed at the canalicular membrane of hepatocytes and exports bile acids into the bile canaliculus. Severe BSEP deficiency causes a dramatic reduction in bile salt secretion into the bile canaliculus, resulting in cholestasis.[1,2]

Children with severe BSEP deficiency typically present with jaundice, pruritus and failure to thrive within the first months of life.[1,2] Serum liver tests reveal elevated serum conjugated bilirubin, alanine aminotransferase (ALT) and bile acid (sBA) levels and normal gamma-glutamyl transpeptidase (γGT) level.[1–3] When measured, biliary bile acid concentration is dramatically low.[1,3,4] Liver biopsy shows intense hepatocellular and canalicular cholestasis with giant cell transformation of hepatocytes.[1,3] Immunohistochemical study of BSEP expression shows a decreased or negative canalicular expression in most patients. Patients carrying biallelic variants predicted to result in a complete absence of expression of functional BSEP at the canalicular membrane present with early-onset cholestasis rapidly progressing toward hepatic fibrosis, cirrhosis, and end-stage liver disease.[5]

Such children with severe *ABCB11* genotypes suffer from intractable pruritus, which is not responding to ursodeoxycholic acid therapy or biliary diversion, and are at high risk for hepatocellular carcinoma.[3,5–9] In patients with severe BSEP deficiency, liver transplantation (LT), indicated in case of end-stage liver disease, evidence of malignancy or severe pruritus not responding to medical therapy, was considered as a definitive curative treatment.[3,10] A first report of an unexplained

* Corresponding author. Address: Service d'Hépatologie et de Transplantation Hépatique Pédiatriques, Hôpital Bicêtre, 78, rue du Général Leclerc, Le Kremlin-Bicêtre, 94275 cedex, France. Tel.: +33 1 45 21 31 68; fax: +33 1 45 21 28 16.
E-mail address: emmanuel.jacquemin@aphp.fr (E. Jacquemin).
† Contributed equally.
https://doi.org/10.1016/j.jhepr.2026.101969

recurrence of a low-γGT PFIC phenotype after LT was described in the 1990s.[11] After the genetic cause of PFIC2 was identified, the hypothesis of a post-LT anti-BSEP alloimmunization was proposed to account for this recurrence.[1] In the same year, the presence of anti-BSEP antibodies able to inhibit bile secretion was reported in the serum of four patients with severe BSEP deficiency and phenotypic recurrence of PFIC2 after LT.[12,13] Although a growing number of case reports and small series have provided evidence that alloimmune anti-BSEP liver disease (AIBD) is a substantial cause of graft loss and of mortality, no standardized screening and treatment protocols have been established.[14–31]

In this retrospective multicenter study, we aimed to assess the prevalence of AIBD in a cohort of patients receiving LT for severe BSEP deficiency and describe their clinical, laboratory and histological features, as well as their outcome and response to treatments. In addition, we compared genotypes, clinical features, and histological characteristics of patients with and without AIBD.

Patients and methods

Patients

We retrospectively collected data from patients receiving LT for severe BSEP deficiency before the age of 18 years between 1988 and 2024 at Bicêtre Hospital (Le Kremlin-Bicêtre, France, n = 22), Femme-Mère Enfants Hospital (Bron, France, n = 6), La Timone Hospital (Marseille, France, n = 5), Necker Enfants Malades Hospital (Paris, France, n = 1), and Cliniques Universitaires Saint-Luc (Brussels, Belgium, n = 1). All patients had biallelic variants in the *ABCB11* gene and underwent at least one testing for anti-BSEP antibodies in serum after LT. Of note, three patients (patients #3, #6, and #7) were previously reported.[14,28] Following its first description in 2009, AIBD screening was progressively implemented in France. Since 2010, all patients with severe BSEP deficiency receiving LT in French hospitals have been prospectively screened for AIBD, whereas sera sampled before 2010 were retrospectively analyzed for AIBD, when available. In Belgium, screening for anti-BSEP antibodies in patients with severe BSEP deficiency was performed in case of suspicion of AIBD. AIBD was defined as the presence of anti-BSEP antibodies in serum, regardless of their titer. In all patients, we retrospectively collected *ABCB11* genotype, type of LT, immunosuppressive (IS) regimen and compliance, LT complications including rejection, viral infections, biliary anatomosis stenosis, as well as liver histological data. Patient characteristics are described in Tables S1 and S2. BSEP and C4d immunostaining were performed on liver slides (antibody reference: HPA019035, Sigma (Darmstadt, Germany), 1:1,500 dilution for BSEP and DB-107 clone A24-T, DB Biotech (Kosice, Slovakia), 1:100 dilution for C4d). *ABCB11* genotypes were classified as severe (biallelic protein-truncating variants, *i.e*. nonsense, frameshift, and/or splicing variants), moderate (one severe and one missense variant), or mild (biallelic missense variants). One small in-frame deletion was considered as a missense variation. Before LT, all patients were treated with ursodeoxycholic acid (daily oral dose of 600 mg/m^2 of body surface area), rifampicin (daily oral dose up to 20 mg/kg); four patients (patients #11, #12, #34, #35) were also treated with 4-phenylbutyrate with some efficacy in two of them (patients #12, #35). For patients developing AIBD, we collected the evolution of clinical and biological manifestations, management, and outcome. Immunological remission was defined as the loss of detectable serum anti-BSEP antibodies in patients with previously established AIBD. Clinical remission was defined as resolution of clinical manifestations and normalization of serum liver tests, including sBA, except when abnormalities could be explained by another complication of LT (biliary obstruction, chronic rejection). AIBD relapse was defined either as a recurrence of anti-BSEP antibodies in serum, or as an increase of anti-BSEP antibody titer in serum associated with recurrence of symptoms (pruritus, jaundice, low-γGT cholestasis).

Approval was obtained from the institutional review board of our institution (AP-HP, N°00011558). According to French legislation, ethics committee agreement is not required for the retrospective collection of data corresponding to current practice. The study conformed to the ethical guidelines of the 1975 Declaration of Helsinki. All patients were informed about the study and none of them refused to be included in the study.

Anti-BSEP antibodies screening and confirmation of anti-BSEP antibodies specificity

All sera were tested in the Pathology Unit of Bicêtre Hospital using indirect immunofluorescence, as previously described.[14] Briefly, cryosections of normal human liver were first incubated with serum samples and bound antibodies were detected using fluorescein isothiocyanate-tagged rabbit anti-human immunoglobulin G (polyclonal FITC-conjugate rabbit anti-human IgG antibody, Dako, [Copenhagen, Denmark] ref F0202). Positive canalicular staining, suggesting the presence of anti-BSEP IgG antibodies bound to the canalicular membrane, was used to diagnose AIBD. When possible, positive serum samples were retested after serial dilution (1/10, 1/50, 1/100, 1/200) to determine an antibody titer. Known positive and negative serum samples were used as controls (Fig. S1). To confirm the anti-BSEP antibodies specificity, indirect immunofluorescence analysis of polarized Can10 cells transduced with rat Bsep-GFP fusion protein incubated with the patient serum was performed, as previously described.[32] Colocalization of the anti-BSEP antibodies (revealed using Alexa Fluor™ 568-conjugated anti-human IgG antibody, Thermofisher Scientific, ref A-21090, dilution 1/500[e]), and of GFP (revealed using anti-GFP monoclonal mouse antibody, Roche (Basel, Swiss) Ref 11814460001, dilution 1/80[e]; and secondary Alexa Fluor™ 488-conjugated anti-mouse IgG antibody, Thermofisher Scientific (Waltham, MA, USA), ref A-11001, dilution 1/500[e]) confirmed the anti-BSEP specificity. Antigenic specificity of anti-BSEP antibodies was studied by ELISA using three peptides designed from potentially immunogenic regions of BSEP: the N-terminal region (amino-acids 21-35; B0 peptide), the first extracellular loop (amino-acids 115-129; B1 peptide), and the C-terminal region of the protein (amino-acids 1297-1312; B2 peptide; described in detail in the Supplementary Methods).

Statistical analysis

Results were summarized as medians and IQRs for continuous variables, and frequency (percentages) for nominal variables. Continuous variables were compared by means of the Mann–Whitney *U* test. The Fisher exact test was performed to

evaluate differences between categorical variables. The value of p <0.05 was considered statistically significant. Survival curves were evaluated using Kaplan–Meier estimates, using R software (version 4.4.2, R Foundation for Statistical Computing, Vienna, Austria).

Results

Systematic screening for anti-BSEP antibodies identified ten patients with AIBD

In total, 35 children received LT for severe BSEP deficiency between 1988 and 2024 in France (n = 34, including 19 patients between 1988 and 2009 and 15 patients between 2010 and 2024) and in Belgium (n = 1). The systematic screening of serum anti-BSEP antibodies was implemented in France in 2010. Therefore, all patients transplanted after 2010 (n = 15) had systematic screening of sera from the time of LT. Regarding patients who underwent LT before 2010 (n = 19), all patients had systematic screening of anti-BSEP antibodies in sera obtained after 2010, whereas retrospective sera could be retrieved and analyzed in only 10 of these 19 patients. In the French cohort (n = 34), serum anti-BSEP antibodies were identified in nine of the 34 patients (26%), in either prospectively collected sera (n = 6) or retrospectively collected sera (n = 3). One additional patient from Belgium, presenting with recurrence of a cholestatic disease after LT, also had anti-BSEP antibodies in serum. Therefore, 10 of 35 patients (29%) were considered as having AIBD. The anti-BSEP specificity of the antibodies was demonstrated in all the six patients tested (Fig. 1). The sera of five patients were further studied to assess the specificity of anti-BSEP antibodies toward selected epitopes using ELISA (Fig. S2). In all cases, reactivity was demonstrated against the first extracellular loop (B1 peptide) and the C-terminus part of BSEP (B2 peptide), similar to previous observations,[19] but not against the N-terminus region of BSEP.

Patients with severe *ABCB11* genotype are more likely to present AIBD

Main characteristics of the 10 patients (three females) who developed AIBD and of the 25 patients (14 females) who did not develop AIBD are described in detail in Tables S1 and S2 respectively, and summarized in Table 1. Before LT, the severity of the manifestations of BSEP deficiency was similar in both groups, as indicated by similar median ages at first

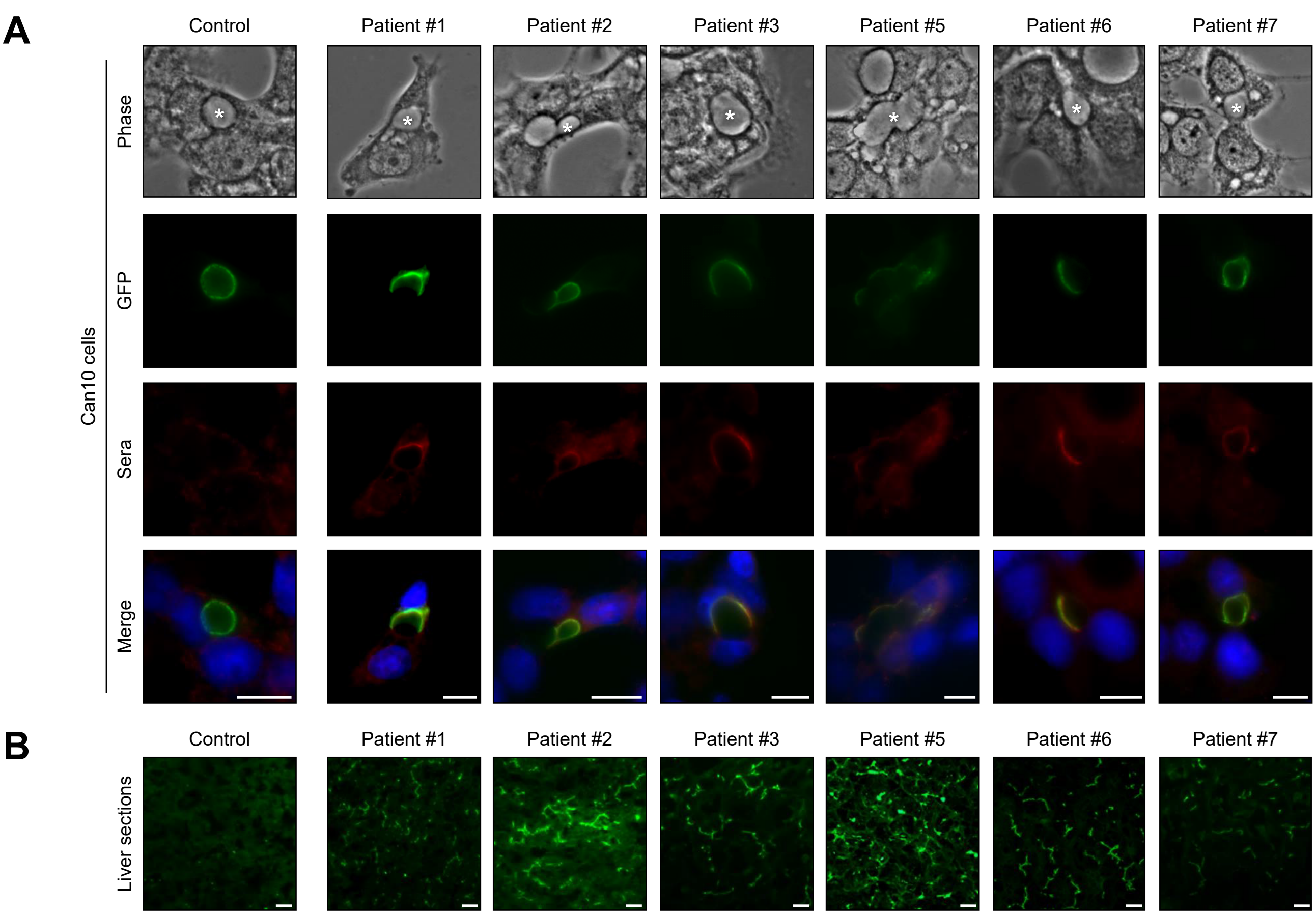

Fig. 1. Indirect immunofluorescence analysis of serum samples of patients with AIBD. (A) Can10 cells transfected with rat Bsep-GFP fusion protein were incubated with patient sera. Putative anti-BSEP antibodies were revealed by the addition of a red fluorochrome-tagged anti-human IgG antibody. GFP was revealed using an anti-GFP monoclonal mouse antibody and a secondary green fluorochrome-tagged anti-mouse antibody. Colocalization of both red and green signals at the canalicular membrane confirmed the specificity of the antibodies against the BSEP protein. Asterisks indicate canaliculi. (B) Indirect immunofluorescence performed on cryosections of normal human liver incubated with patient sera. Use of a green fluorochrome-tagged-anti-human IgG secondary antibody revealed a canalicular membrane staining suggestive of the presence of anti-BSEP antibodies in sera. BSEP, bile salt export pump; GFP, green fluorescent protein.

symptoms (3.5 *vs.* 3 months of age, p = 0.60) and at LT (4.7 *vs.* 7.0-year-old, p = 0.47) and a similar proportion of patients who underwent LT for a severe complication of BSEP deficiency such as cirrhosis, liver failure, and/or hepatocellular carcinoma (70% *vs.* 68%, p = 1.0). Severe *ABCB11* genotype appears to be the major risk factor of AIBD, with most of the patients with AIBD harboring a severe genotype (80%), compared with 28% of patients without AIBD (p = 0.01). In our cohort, eight of 15 patients (53%) with severe *ABCB11* genotypes developed AIBD, in contrast to two of 13 (15%) with moderate genotypes and none with mild genotypes. By contrast, BSEP immunostaining assessed in native liver did not predict AIBD, as it was negative in all patients with AIBD (n = 8/8) and in 90% of patients without AIBD (n = 19/21, p = 1.0). No difference in type of donor, IS treatment, or post-LT complications (T-cell-mediated rejection, biliary obstruction, presence of donor-specific antibodies) were identified, nor more severe ischemia–reperfusion injuries (data not shown), except a higher occurrence of cytomegalovirus (CMV) infection in the group of patients with AIBD which almost reached significance (20% *vs.* 0%, p = 0.08). Duration of follow-up was 14.2 and 12.6 years post-LT in median in both groups.

AIBD initially manifests as intrahepatic cholestasis with pruritus

The initial clinical and laboratory characteristics of the 10 patients developing AIBD are detailed in Table 2. The median age at AIBD diagnosis and interval between LT and AIBD were 11.1 years (IQR: 8–16, range 4.2–20.7 years) and 5 years (IQR: 3.7–8.3, range: 1.5–12 years), respectively. Notably, for patient #2, AIBD was confirmed 1 year after a second LT, performed for graft loss following recurrence of a cholestatic disease compatible with AIBD. Before the first serum positivity, a decrease of IS exposure was identified in four cases, because of an adjustment of the IS regimen (patients #3 and #7) or because of non-adherence to treatment (patients #4 and #9). Nine of 10 patients had symptoms at the time of first detection of anti-BSEP antibodies in serum, including pruritus (n = 9), jaundice (n = 5) and hypocholic stools (n = 3). One patient (#10) experienced AIBD-related manifestations 2 years after detection of anti-BSEP antibodies. All patients tested had elevation of sBA at the time they experienced clinical manifestations of AIBD, with a median sBA concentration of 244 µmol/L (range: 44–587), contrasting with low values of serum γGT level (<40 U/L in eight of 10 patients) (Table 2). By contrast, the median sBA concentration was 10 µmol/L (range: 4–43 µmol/L) during follow-up in the 10 patients without AIBD in whom the data were available.

AIBD mimics histological features of severe BSEP deficiency and is associated with positive canalicular C4d staining

Liver biopsies were analyzed in nine patients at AIBD onset (Table 3). Most patients exhibit canalicular cholestasis and giant transformation of hepatocytes (n = 7/9 for each, 78%), mimicking the phenotype of patients with severe BSEP deficiency (Fig. S3). Three patients had concomitant moderate T-cell-mediated rejection (TCMR). Fibrosis was moderate with liver allograft fibrosis score (LAFSc) between 2 and 5 in the six biopsies evaluated. Finally, BSEP immunostaining was reduced in four of seven patients tested, while MDR3 was not (Fig. 2A,B), and revealed normal canalicular BSEP expression in three patients. Anti-C4d immunostaining performed in five

Table 1. Main characteristics of patients with and without AIBD following liver transplantation for severe BSEP deficiency.

	Patients with AIBD (n = 10)	Patients without AIBD (n = 25)	*p* values
Female sex	3/10 (30)	14/25 (56)	0.26
Age at initial symptoms (in months)	3.5 (2.3–7.3)	3.0 (1.0–5.1), n = 20	0.60
ABCB11 genotype*			
Severe	8/10 (80)	7/25 (28)	**0.01**
Moderate	2/10 (20)	11/25 (44)	
Mild	0/10	7/25 (28)	
Absent BSEP staining on native liver	8/8 (100)	19/21 (90)	1.0
Age at LT (years)	4.7 (3.0–8.1)	7.0 (3.5–9.4)	0.47
Indication for LT			
Severe pruritus	3/10 (30)	8/25 (32)	1.0
Complication of BSEP deficiency (cirrhosis, LF, HCC)	7/10 (70)	17/25 (68)	
Deceased donor	9/10 (90)	22/25 (88)	1.0
Immunosuppression after LT			
Tacrolimus-based	5/9 (56)	20/25 (80)	0.20
Cyclosporine-based	4/9 (44)	5/25 (20)	
Combination with Aza or MMF (within the first year after LT)	6/9 (67)	12/25 (48)	0.45
Complications after LT (besides AIBD)			
Acute T-cell-mediated rejection	7/10 (70)	16/25 (64)	1.0
BA stricture	5/10 (50)	8/25 (32)	0.44
Symptomatic CMV infection	2/10 (20)	0/25	0.08
Positive DSA†	2/7 (29)	6/17 (35)	1.0
Duration of follow-up after LT (years)	14.2 (10.2–24.5)	12.6 (6.2–15.6)	**0.03**
Retransplantation	6/10 (60)	1/25 (4)	**0.0008**
Death	3/10 (30)	1/25 (4)	0.06

Data are presented as n/N (%) or median (IQR). The p values <0.05 are indicated in bold. AIBD, alloimmune anti-BSEP liver disease; Aza, azathioprine; BA, biliary anastomosis; BSEP, bile salt export pump; CMV, cytomegalovirus; DSA, donor-specific antibodies; HCC, hepatocellular carcinoma; LF, liver failure; LT, liver transplantation; MMF, mycophenolate mofetil.

*Genotypes defined as severe (biallelic protein-truncating variants), moderate (one protein-truncated variant and one missense variant), and mild (biallelic missense variant).

†Positive DSA are HLA class II donor-specific antibodies with mean fluorescence intensity above 1,000. Statistical analysis was performed using Fisher exact test (qualitative variables, including 3 × 2 Fisher test for *ABCB11* genotype analysis) or Mann–Whitney *U* test (quantitative variables).

Table 2. Main clinical and laboratory features at diagnosis of AIBD.

Patient	*ABCB11* genotype	Interval from LT (years)	Events in the preceding 3 months	Symptoms (at diagnosis of AIBD)	Liver function tests (at diagnosis of AIBD): ALT (U/L)	γGT (U/L)	Total/conjugated bilirubin (μmol/L)	sBA (μmol/L)	Anti-BSEP antibodies (titer)
#1	Severe	4.9	UDCA treatment interruption	Pruritus, jaundice, hypocholic stools	36	12	77/66	368	Positive (1/50)
#2	Moderate	8.7 (1 yr after reLT)	None	Pruritus, jaundice	739	36	349/NA	190	Positive (NA)
#3*	Moderate	3.3	Decrease of IS	Pruritus	160	35	32/NA	107	Positive (NA)
#4	Severe	5.1	Non-compliance to IS regimen	Pruritus	727	66	23/20	60	Positive (1/200)
#5*	Severe	3.2	Biliary obstruction, recurrent cholangitis episodes	Pruritus	58	22	11	44	Positive (1/1)
#6	Severe	12.5	None	Pruritus, jaundice	184	50	287/152	533	Positive (1/10)
#7*	Severe	4.8	Decrease of IS	Pruritus, hypocholic stools	31	14	32/22	298	Positive (1/10)
#8	Severe	7	Jejunal intussusception after a viral infection, TCMR	Pruritus, jaundice	77	37	103/NA	NA	Positive (NA)
#9	Severe	1.5	Non-compliance to IS regimen	Pruritus, jaundice, hypocholic stools	28	13	186/176	587	Positive (1/200)
#10	Severe	12	None	No symptoms	36	8	21/8	6.2	Positive (1/10)
		14	None	Pruritus & jaundice	525	13	169/119	NA	Positive (1/200)

Upper limit of normal: <40 U/L (ALT), <40 U/L (γGT), <17 μmol/L (bilirubin), and <10 μmol/L (sBA). *Indicates patients diagnosed retrospectively. AIBD, alloimmune anti-BSEP liver disease; ALT, alanine aminotransferase level; BSEP: bile salt export pump; γGT, γ-glutamyl transferase level; IS, immunosuppression; LT, liver transplantation; NA, not available; reLT, liver retransplantation; sBA, serum bile acids level; TCMR, T-cell-mediated rejection; UDCA, ursodeoxycholic acid.

Table 3. Liver graft histological characteristics at diagnosis of AIBD.

Patient	Time after LT (years)	Histological features	Fibrosis (LAFSc)	TCMR (BANFF)	BSEP immunostaining	C4d immunostaining	AMR
#1	4.9	Severe canalicular cholestasis; rare giant hepatocytes; mild centrilobular and portal inflammation; biliary obstruction	4 (2 + 0 + 2)	No	**Reduced canalicular expression**	**Positive** (canalicular, lateral)	No
#2*	9.0	**Explanted liver**: severe cholestasis, giant hepatocytes	7 (2 + 2 + 3)	No	Normal canalicular expression	NA	NA
#4	5.2	Lobular and portal hepatitis; rare giant hepatocytes	2 (1 + 1 + 0)	Yes (6: 3 + 1 + 2)	Normal canalicular expression	**Positive** (canalicular)	No
#5	4.8	Moderate portal and mild perivascular inflammation	2 (1 + 0 + 1)	No	Normal canalicular expression	NA	Yes
#6	13	Canalicular cholestasis; giant hepatocytes Moderate portal inflammation with ductular proliferation.	5 (3 + 2 + 0)	Yes (3: 2 + 0 + 1)	**Reduced canalicular expression**	Negative	No
#7*	4.8	Canalicular cholestasis, giant hepatocytes Slight centrilobular fibrosis	NA	NA	NA	NA	NA
#8	7	Canalicular cholestasis, giant hepatocytes Portal inflammation	3 (1 + 2 + 0)	Yes (5: 2 + 2 + 1)	**Reduced canalicular expression**	**Positive** (canalicular)	No
#9*	1.5	Cholestasis with ductopenia and bile duct dystrophy	NA	NA	NA	NA	NA
#10	14.3	Cholestasis; giant hepatocytes	NA	No	**Reduced canalicular expression**	**Positive** (canalicular)	No

Liver biopsies were performed at diagnosis. For patient #10, biopsy was performed 2 years after initial positivity of anti-BSEP antibodies, at the onset of symptoms. LAFSc is expressed as the sum of a 0–3 score referring to portal tract, sinusoids, and central vein fibrosis (total score: 0-9).[39] TCMR was graded using BANFF score, expressed as the sum of a 0–3 score referring to portal tract inflammation, bile duct inflammatory lesions, and venous endothelial inflammation (max 0–9).[40] All biopsies were reviewed by a single pathologist of Bicêtre Hospital, unless indicated by the symbol '*'. AIBD, alloimmune anti-BSEP liver disease; AMR, antibody-mediated rejection; BSEP, bile salt export pump; C4d, complement component 4d; LAFSc, liver allograft fibrosis score; LT, liver transplantation; NA, not available; TCMR, acute T-cell-mediated rejection.

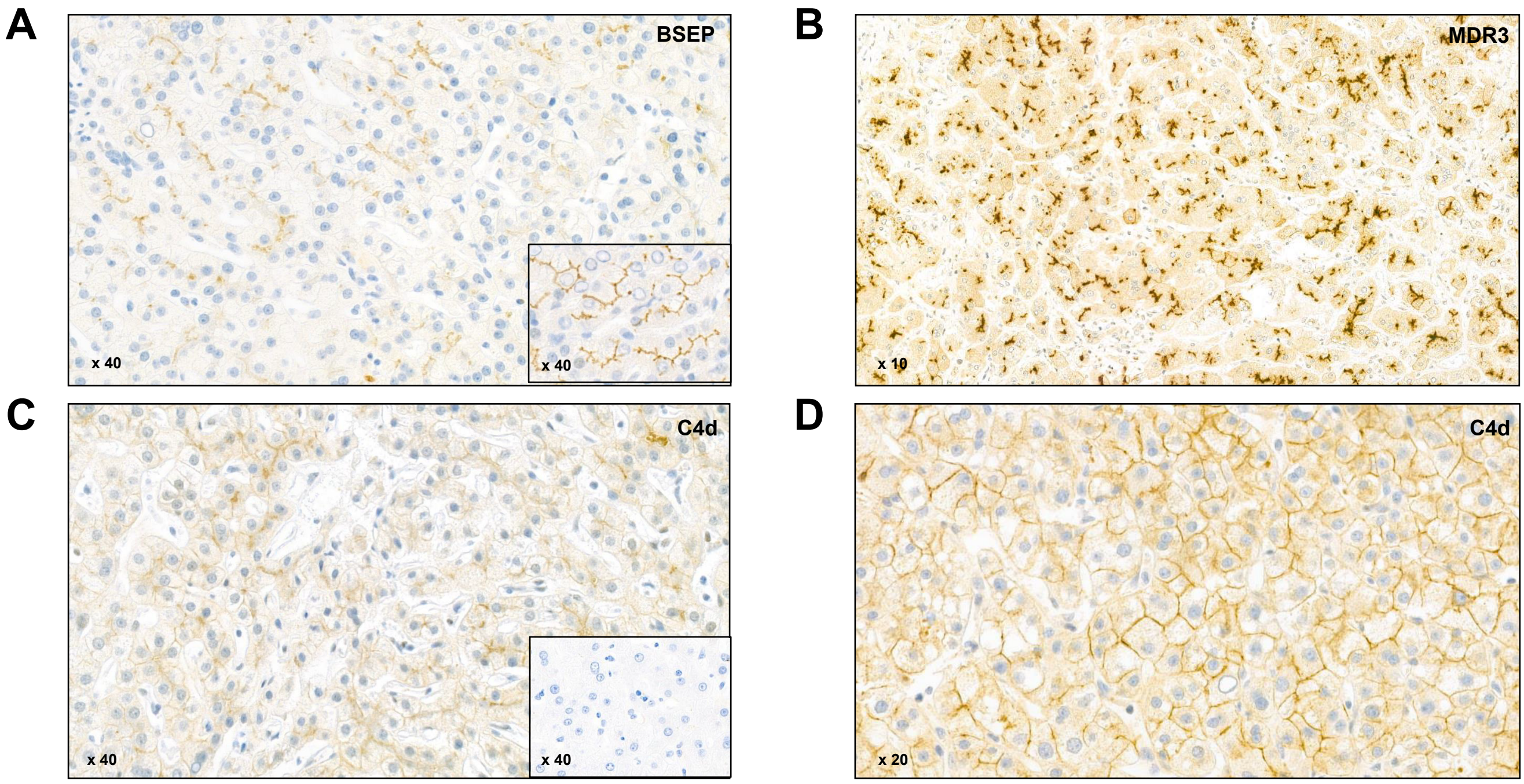

Fig. 2. Representative images of reduced BSEP staining and of positive canalicular C4d staining in a patient with AIBD. (A,B) Reduced canalicular BSEP staining (A) on the biopsy of patient #1 at AIBD relapse, contrasting with a conserved canalicular MDR3 staining (B). In panel A, insert shows a normal positive BSEP staining as control. (C,D) C4d staining on the biopsy of patient #1 at relapse (C) and 1 month after relapse (D), showing focal canalicular C4d staining (C) and diffusely membranous C4d staining with canalicular enhancement (D). Insert shows a negative C4d staining as control (from a patient without AIBD). AIBD, alloimmune anti-BSEP liver disease; BSEP, bile salt export pump; C4d, complement component 4d.

patients did not show signs of chronic antibody-mediated rejection (AMR), but revealed focal or diffuse canalicular staining in four patients (Fig. 2C,D). Analysis of follow-up liver biopsies and explanted livers is described in Table S3, further demonstrating that AIBD histological features mimic the one of severe BSEP deficiency and that canalicular positive C4d staining is frequently observed along the course of AIBD (five of six patients tested).

By contrast, study of the 17 biopsies performed after LT in 11 patients who did not develop AIBD (Table S4) did not show positive canalicular C4d staining, identified cholestasis in only two patients, giant transformation of hepatocytes in one patient (early after LT), and reduced BSEP staining in one patient. Therefore, the presence of giant hepatocytes, reduced BSEP immunostaining, and/or positive canalicular C4d staining may strengthen the suspicion of AIBD in patients receiving LT for severe BSEP deficiency.

AIBD has a severe outcome and requires specific intensification of immunosuppressive therapy

Management of AIBD is detailed in Table S5 and summarized in Fig. 3. Of note, the three patients diagnosed with AIBD retrospectively were initially considered as having rejection episodes with adaptation (patients #3 and #5) or intensification (patient #7) of IS regimen. The seven other patients, whose serum positivity was detected prospectively, underwent intensification of IS regimen based on a combination of rituximab (n = 6), plasmapheresis (n = 5), intravenous immunoglobulins (IVIg) (n = 5), pulse steroids (n = 4), and/or immunoadsorption (IA) (n = 2). Two of these seven patients required retransplantation (reLT) for graft failure early after AIBD diagnosis (patients #6 and #9).

Clinical remission was achieved in nine of 10 patients, including one patient (patient #8) with persistent ALT and γGT increase attributable to chronic TCMR while displaying negative anti-BSEP antibodies. The single patient (patient #2) for whom remission was not obtained was diagnosed with AIBD 1 year after reLT for graft loss likely because of unrecognized AIBD. He did not respond to intensification of IS and died of recurrent graft failure as a result of AIBD. In patients who achieved clinical remission, anti-BSEP antibody titers were stable in two (retrospectively diagnosed patients #5 and #7), decreased in two (patients #4, #9), or were negative in four (patients #1, #6, #8, #10). Of note, decreased or negative anti-BSEP antibody titers were only observed in patients who had received either rituximab and/or plasmapheresis/IA.

Several IS regimens were used following the first episode of AIBD and were considered as 'maintenance therapies' (Fig. 3), including repeated IVIg (patients #4, #8) and/or rituximab administrations (patients #4 and #10) in three patients. Interestingly, only one of these three patients had a relapse of AIBD (patient #4), favored by a complete interruption of all IS therapy (including tacrolimus and steroids). This patient had very low anti-BSEP antibody titers (1/1) before IS interruption. In the two other patients (patients #8 and #10), prolonged remission with negative anti-BSEP antibody titers was achieved. By contrast, five of the six patients who did not receive IVIg or rituximab as a maintenance therapy exhibited a relapse of AIBD. Some correlation between B-cell depletion and anti-BSEP antibody titers could be observed, with recurrence of high anti-BSEP

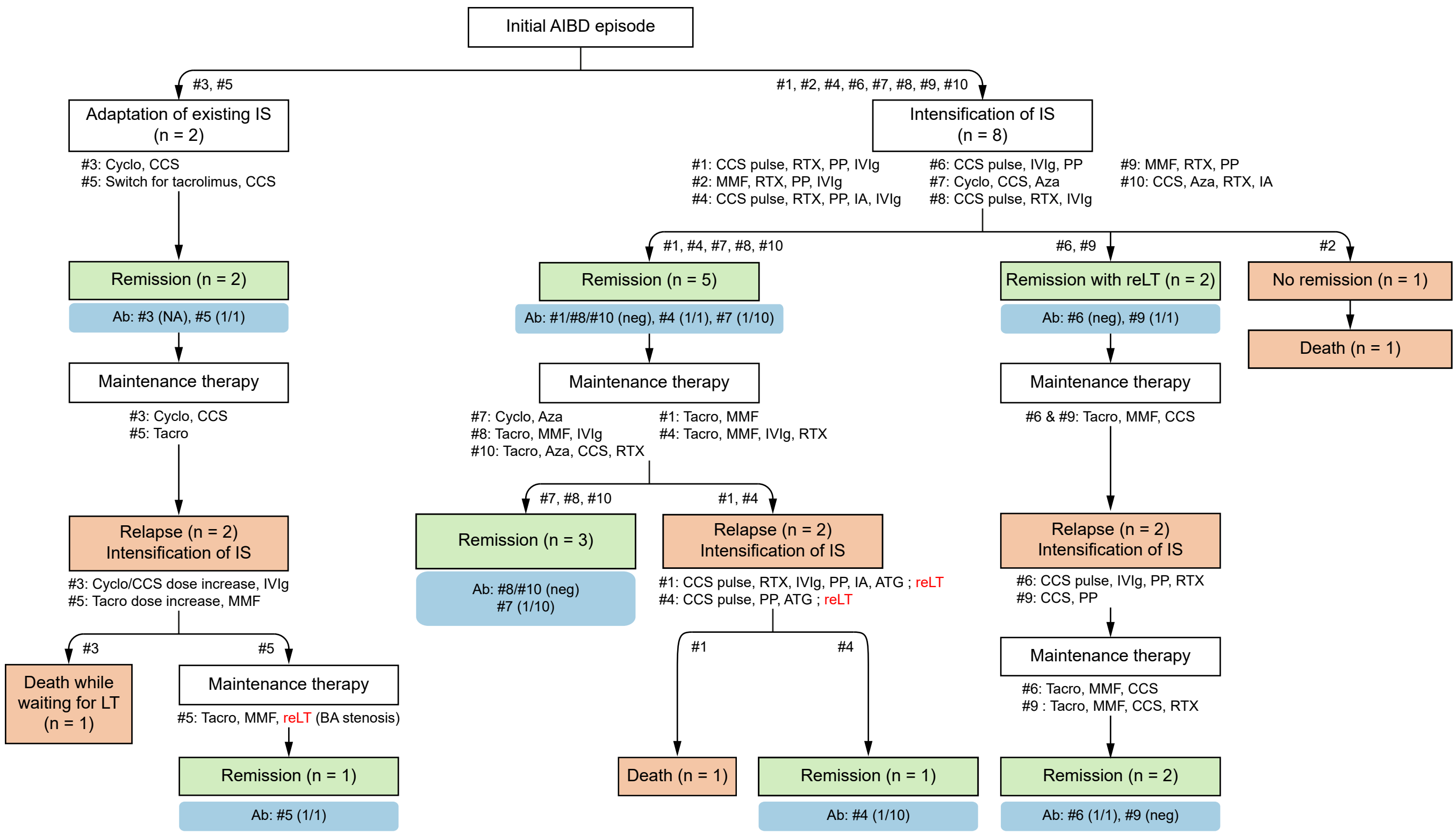

Fig. 3. Treatment and outcome of the 10 patients with AIBD. Remission was defined as clinical remission (resolution of clinical manifestations and normalization of serum liver tests, including serum bile acid levels, except if abnormalities could be explained by another complication of liver transplantation such as biliary obstruction or chronic rejection). Ab, anti-BSEP antibody titers; AIBD, alloimmune anti-BSEP liver disease; ATG, anti-thymocyte globulins; Aza, azathioprine; BA, biliary anastomosis; CCS, corticosteroids; Cyclo, cyclosporine; IA, immunoadsorption; IS, immunosuppression; IVIg, intravenous immunoglobulins; LT, liver transplantation; MMF, mycophenolate mofetil; NA, not available; PP, plasmapheresis; reLT, liver retransplantation; RTX, rituximab; Tacro, tacrolimus.

antibodies (titer >1/100) in patient #1 and #4 at relapse associated with B-cell counts ≥10%.

Overall, six patients exhibited a relapse of AIBD in median 6 years (range: 1.7–14 years) after the initial episode. An intensification of IS therapy was performed in all patients. It enabled clinical remission in four of them while the two other patients (patients #1 and #3), developed progressive liver failure and died because of AIBD, despite reLT in one (patient #1). Among the four patients who achieved clinical remission after relapse, only one (patient #9) achieved immunological remission, which was maintained with repeated rituximab administrations. Anti-BSEP antibody titers decreased in the three other patients. Two of them required a reLT for graft failure because of AIBD and IS interruption (patient #4) or for cirrhosis with concomitant biliary obstruction (patient #5). Detailed evolution of serum liver tests and serum anti-BSEP antibody titers of patients #1, #4, #8, #9, and #10 are provided in Fig. S4.

In total, six patients (60%) with AIBD required reLT, in contrast to one of 25 (4%) in the group of patients without AIBD (p = 0.0008). Three patients with AIBD (30%) and one patient without AIBD (4%) died (Table 1). Significantly poorer graft survival is observed in patients with AIBD, compared with patients without AIBD (p = 0.0024), as depicted in Fig. 4.

Discussion

The present work, studying a cohort of patients with severe BSEP deficiency in whom serum anti-BSEP antibodies screening was systematically performed after LT, enables the estimation of the frequency of AIBD and the identification of its risk factors. In addition, this large cohort of patients with AIBD also provides insights into the outcome of AIBD and the impact of therapeutic interventions.

The frequency of AIBD observed in our cohort (29%) is in line with previous studies reporting a prevalence ranging between 8% and 33%.[15,31,33] These data show that AIBD is, in fact, a frequent complication after LT in patients with severe BSEP deficiency, occurring in up to 50% of patients with severe *ABCB11* genotype. Therefore, patients with severe BSEP deficiency, and their family, should be informed before LT of the risk of AIBD. The mechanism responsible for anti-BSEP antibodies development after LT is unknown but likely involves multiple pathophysiological processes.[13,15,21,25,33] It has been suggested that severe *ABCB11* genotypes, that is genotypes leading to the complete absence of BSEP protein expression, prevent the establishment of immune tolerance toward BSEP before LT, therefore favoring AIBD after LT.[15,25,33] Our results, demonstrating that a severe *ABCB11* genotype is the major risk factors of AIBD, support this hypothesis. By contrast, a negative liver BSEP immunostaining did not predict the occurrence of AIBD. One hypothesis would be that a complete absence of BSEP expression or an intracellular retention of abnormal BSEP similarly lead to a negative BSEP immunostaining, but may result in different presentation of BSEP antigens to the immune system. Pre-LT expression of

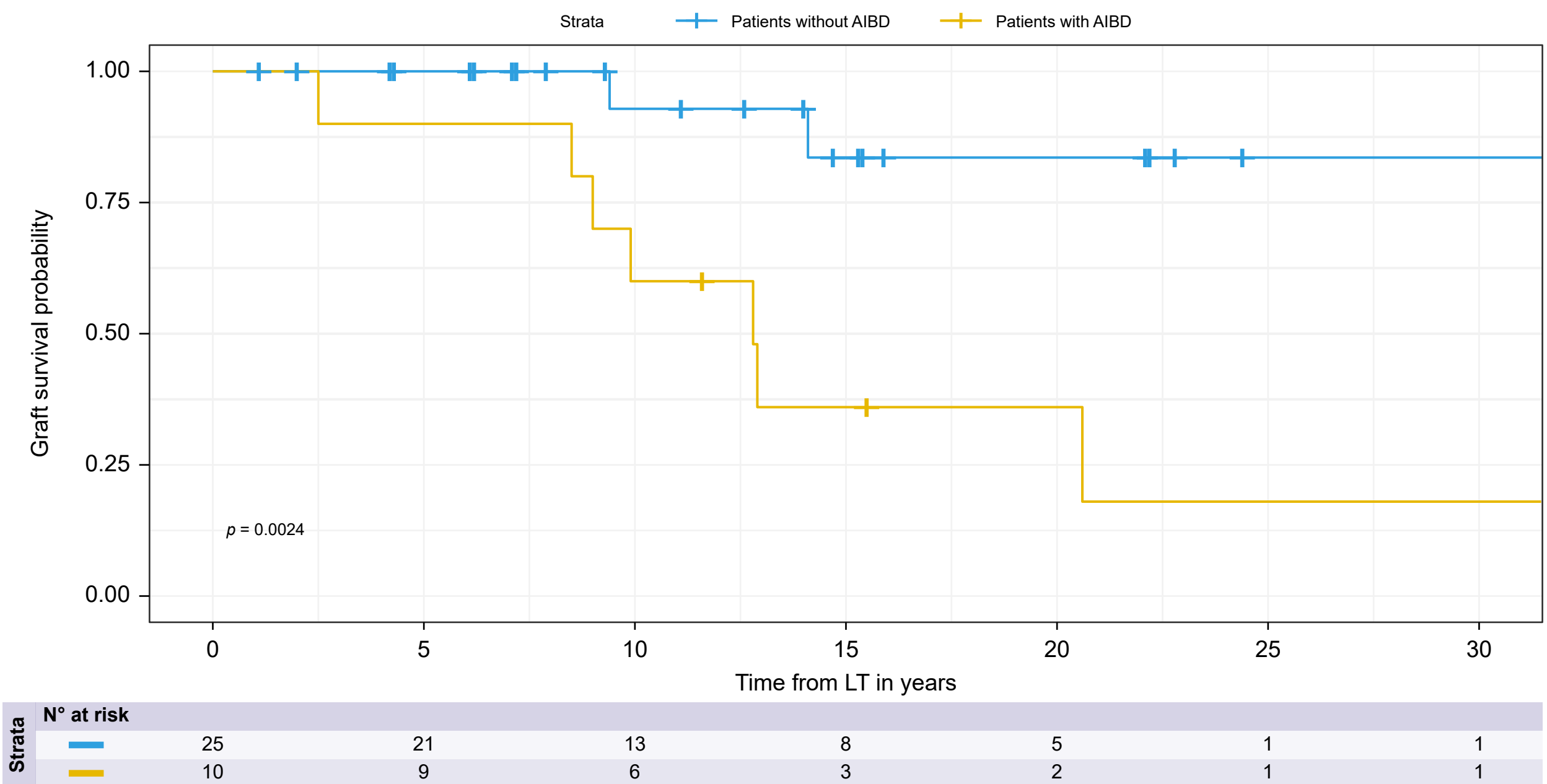

Fig. 4. Graft survival curve of the 35 patients according to the presence of AIBD. Patients were censored at the time of retransplantation or death. Survival curve was made using Kaplan–Meier estimates, using the R software (version 4.4.2; R Foundation for Statistical Computing, Vienna, Austria). Log-rank test was used for comparison. AIBD, alloimmune anti-BSEP liver disease; LT, liver transplantation.

BSEP antigens, for example in patients with missense variants leading to intracellular retention of BSEP, would be required to achieve immune tolerance toward BSEP. In line with this, no patients with normal BSEP immunostaining on native liver have been described in the literature as having developed AIBD.

Other factors beyond genotypes are likely involved in the development of AIBD, as evidenced by the fact that several patients with and without AIBD shared common (patients #8/#10 and #19; #1 and #33) or equivalent genotypes (patients #3 and #15). The rather long time interval between LT and AIBD observed in our study, as in others,[33] further suggests that a 'second hit' is required to trigger AIBD. Our data suggest that reduction of the IS regimen, as previously reported,[12,19,25] and CMV infection could have contributed to the onset of AIBD. Our data do not provide evidence for a contribution of TCMR or severity of ischemia–reperfusion injuries to the onset of AIBD.

In line with literature data,[33] most patients with AIBD present with a clinical phenotype mimicking the one of severe BSEP deficiency (*i.e.* a normal-γGT cholestasis associated with severe pruritus and high elevation of sBA) both at onset and at the time of relapse. The observation of such manifestations during the post-LT course raises suspicion of AIBD and should prompt the screening for anti-BSEP antibodies in the serum. In any case, considering the frequency and the severity of AIBD, systematic screening for anti-BSEP antibodies should be performed routinely even in the absence of AIBD manifestations, especially in patients with severe *ABCB11* genotypes.[34] Furthermore, our data suggest that anti-BSEP antibodies may precede AIBD symptoms (patient #10).

Liver biopsy is a central tool in the management of liver transplanted patients in case of abnormal serum liver tests. In patients who received LT for severe BSEP deficiency, graft biopsy may provide evidences for AIBD showing canalicular cholestasis with giant cell transformation and positive canalicular C4d staining, while ruling out potential differential diagnosis. A positive canalicular C4d staining was rather sensitive and specific of AIBD in our study, in line with previous reports.[23,29] This likely reflects the central pathophysiological role of anti-BSEP antibodies at the canalicular membrane in AIBD. By contrast, BSEP immunostaining on liver graft was already reported to be either absent,[14] reduced,[14,23,26,29] or conserved,[12,16] depending on the studies. This variability might be due to a potential competition of alloimmune anti-BSEP antibodies, present in the patient sera, with the different commercial or in-house anti-BSEP antibody clones used for BSEP immunostaining. Some of these antibodies, such as the one designed by Stieger *et al.*,[35] recognize the C-terminal domain of BSEP which is frequently targeted by alloimmune antibodies in AIBD[19] , as observed in our study. Of note, the antibody used in our study recognizes the nucleotide-binding domain 1, which has not been reported to be targeted by anti-BSEP antibodies. In addition, higher titers of anti-BSEP antibodies might interfere with BSEP immunostaining, whereas low titers might not. In patient #1, the only patient for whom multiple histological data and anti-BSEP antibody titers were available during the same episode (Table S3), the decrease of BSEP immunostaining correlated with antibody titers, with a diffuse (normal) canalicular BSEP immunostaining once the titers fell below 1/10.

Treatments of AIBD used in our cohort combined steroids (oral or pulse), rituximab, plasmapheresis, IA and/or IVIg, similarly to those reported previously.[12,16,17,20–23,25,26] The aim of these strategies is to reduce the amount of circulating anti-BSEP antibodies and/or to deplete antibody-producing plasma cells. Given the central role of these antibodies in the

pathogenicity of AIBD, achieving negative anti-BSEP antibodies in serum is likely to offer remission and prevent AIBD relapse. A recent meta-analysis showed that rituximab-based treatment improved AIBD in >80% of patients.[33] Plasma cell depletion using bortezomib also proved efficient in recent case reports.[29,30] Moreover, hematopoietic stem cell transplantation was able to suppress serum anti-BSEP antibody production in a patient with refractory AIBD.[24] In our cohort, treatment with rituximab and/or plasmapheresis/IA was used as a first-line therapy in all seven patients with prospectively-identified AIBD. These treatments enabled a reduction or a negativation of serum anti-BSEP antibodies in all patients but one. Regarding relapse, most of the patients (five out of six patients) that did not receive a maintenance therapy based on rituximab or IVIg did experience a relapse of AIBD. By contrast, the use of such maintenance treatment in three patients enabled prolonged survival with negative or very low anti-BSEP antibody titers in all, although one patient relapsed at the time of complete interruption of the IS regimen. Similarly, one patient (patient #9) achieved initial AIBD remission with very low anti-BSEP antibody titer (1/1), but relapsed when receiving tacrolimus, oral steroids, and mycophenolate mofetil (MMF) maintenance therapy. After the second remission achieved using plasmapheresis, repeated rituximab administration enabled persistent remission with negative anti-BSEP antibodies in serum.

Our data confirm that immunological remission should be the main goal of the treatment, both to obtain AIBD remission and to prevent relapse. This likely requires specific maintenance therapies such as IVIg and/or rituximab. Our data suggest that B-cell counts <5% could be a therapeutic target to maintain low serum anti-BSEP antibody titers and prevent AIBD relapse. Therefore, B-cell counts, together with anti-BSEP antibodies, should be monitored closely in AIBD patients. A specific issue is the management of 'refractory' AIBD, as observed in patient #2 at diagnosis and in patient #1 at relapse, which did not respond to very intensive medical therapy and led to the death of the two patients. The use of new therapeutics developed for antibody-mediated rejection, such as the IgG-cleaving enzyme imlifidase,[36] could enable a short antibody-free window, during which reLT could be performed with an expected better outcome. More efficient B-cell depletion using specific chimeric antigen receptor T cells, as recently reported in patients with IgG4-related disease, or new B cells depleting drugs such as inebilizumab, might also prove useful.[37,38] Lastly, considering the high AIBD frequency (~50%) observed in patients with severe *ABCB11* genotypes, another open question is the possibility to prevent its occurrence by preventive IS regimen. In two patients with either a mild (patient #11) or severe *ABCB11* genotype (patient #24), early MMF introduction and repeated post-LT IVIg administrations were used, without AIBD occurrence during a 4-year follow-up.

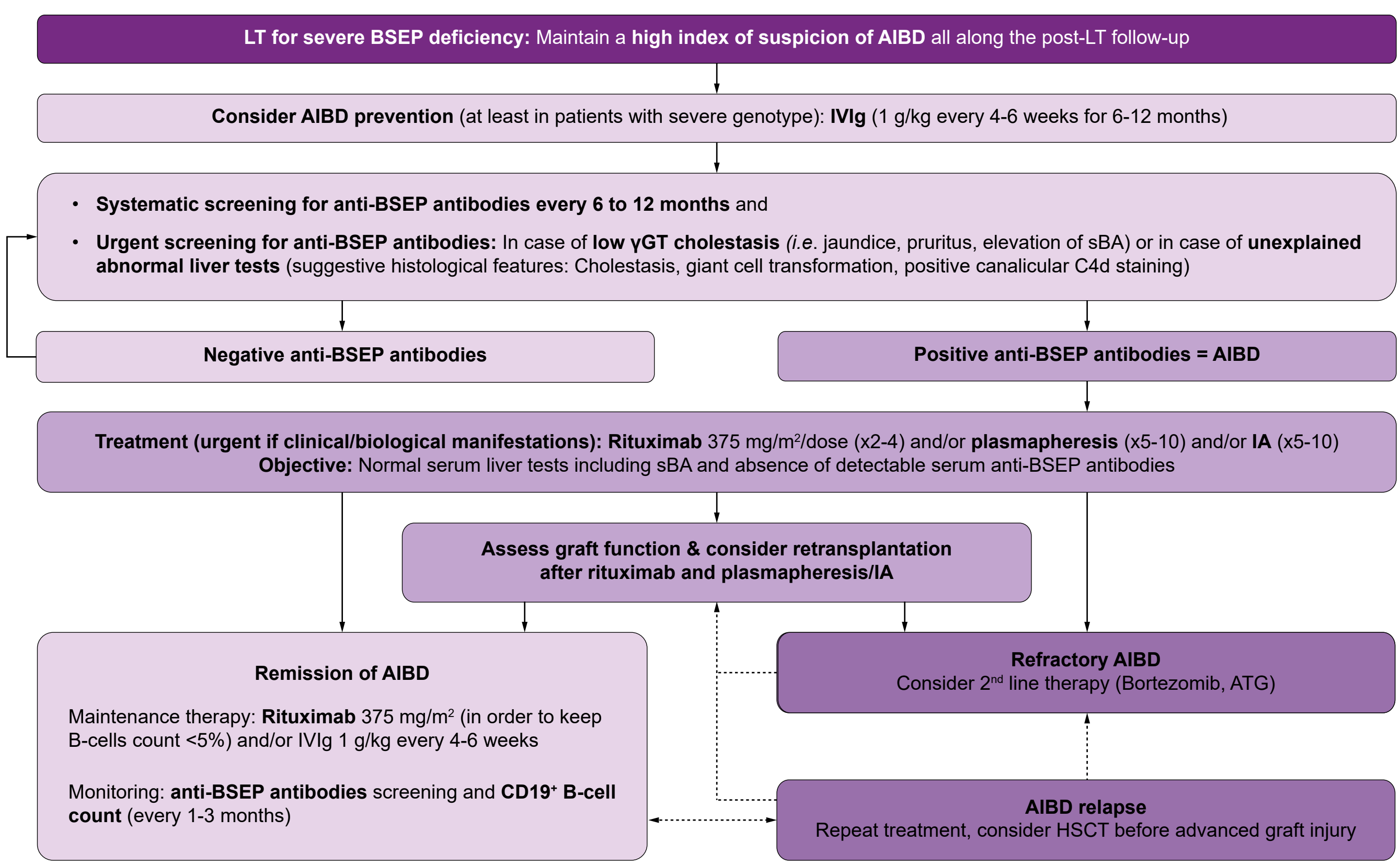

Fig. 5. Proposed algorithm for the prevention, screening and management of AIBD, as performed in our department. This is based solely on expert opinion, considering our clinical practice and the results presented in this publication. AIBD, alloimmune anti-BSEP liver disease; ATG, anti-thymocyte globulins; BSEP, bile salt export pump; C4d, complement fragment 4d; HSCT, hematopoietic stem cell transplantation; IA, immunoadsorption; IVIg, intravenous immunoglobulins; LT, liver transplantation; sBA, serum bile acid levels.

Our study is the largest cohort reporting on risk factors, prevalence, management, and outcome of AIBD following LT in patients with severe BSEP deficiency to date. However, several limitations exist, mostly because of the retrospective nature of this work, such as missing data and heterogeneity in management over the years. Notably, the study's duration of 25 years, with many patients receiving LT before awareness of AIBD, required the use of both prospectively and retrospectively collected sera. This long duration, together with the multicentric nature of the study, explained the wide heterogeneity in AIBD management and follow-up. These limitations highlight the need for AIBD diagnosis and management guidelines. Considering the rarity of severe BSEP deficiency, evidence to guide AIBD management are unlikely to be obtained through controlled clinical trials and will rather be based on lower-grade evidences. Standardization of AIBD diagnosis and treatment protocols is required to enable future international prospective studies. Based on our experience, we propose a specific prevention, screening, and management of AIBD in patients with severe BSEP deficiency (detailed in Fig. 5).

AIBD is a frequent complication of LT in patients with severe BSEP deficiency, especially in patients with severe *ABCB11* genotypes, this population of patients being the one typically requiring LT early in life. Screening for serum anti-BSEP antibodies should be routinely and regularly offered to these patients for early diagnosis and treatment of AIBD, given its frequency and severity. First-line therapy using rituximab and/or plasmapheresis/IA and maintenance therapy using rituximab and/or IVIg should be considered to obtain prolonged immunological remission and improve outcome.

Affiliations

[1]*Pediatric Hepatology and Liver Transplantation Unit, Bicêtre Hospital, Assistance Publique - Hôpitaux de Paris (AP-HP), Paris-Saclay University, le Kremlin-Bicêtre, France;* [2]*Université Paris-Saclay, Univ Evry, Inserm, Genethon, Integrare research unit UMR_S951, Evry, France;* [3]*Liver Unit, Meyer Children's Hospital IRCSS, Florence, Italy;* [4]*Pathology Unit, Bicêtre Hospital, Assistance Publique - Hôpitaux de Paris, Paris-Saclay University, Le Kremlin-Bicêtre, France;* [5]*Université Paris-Saclay, Inserm, Physiopathogénèse et traitement des maladies du foie, UMR_S 1193, FHU Hepatinov, Orsay, France;* [6]*Hepatology Unit, Croix Rousse Hospital, Lyon, France;* [7]*Hepatology and Gastroenterology Unit, Timone Hospital, Marseille, France;* [8]*Pediatric Hepatology Unit, Necker Hospital, Assistance Publique - Hôpiaux de Paris, Paris, France;* [9]*Hepatology Unit, Paul Brousse Hospital, Assistance Publique - Hôpitaux de Paris, Villejuif, France;* [10]*Pediatric Gastroenterology and Hepatology Unit, Timone Hospital, Marseille, France;* [11]*Pediatric Gastroenterology and Hepatology Unit, Bron Hospital, Bron, France;* [12]*Pediatric Hepatology Unit, Saint-Luc Hospital, Louvain University, Bruxelles, Belgium;* [13]*Hepatogastroenterology and Liver Transplant Unit, Bambino Gesù Children's Hospital, Rome, Italy*

Abbreviations

Ab, anti-BSEP antibody titers; AIBD, alloimmune anti-BSEP liver disease; ALT, alanine aminotransferase level; AMR, antibody-mediated rejection; ATG, anti-thymocyte globulins; Aza, azathioprine; BA, biliary anastomosis; BSEP, bile salt export pump; C4d, complement component 4d; CCS, corticosteroids; CMV, cytomegalovirus; Cyclo, cyclosporine; DSA, donor-specific antibodies; HCC, hepatocellular carcinoma; HSCT, hematopoietic stem cell transplantation; IA, immunoadsorption; IS, immunosuppression; IVIg, intravenous immunoglobulins; LAFSc, liver allograft fibrosis score; LF, liver failure; LT, liver transplantation; MMF, mycophenolate mofetil; NA, not available; PFIC2, progressive familial intrahepatic cholestasis type 2; PP, plasmapheresis; reLT, liver retransplantation; RTX, rituximab; sBA, serum bile acids; Tacro, tacrolimus; TCMR, T-cell-mediated rejection, UDCA, ursodeoxycholic acid; γGT, gamma-glutamyl transferase.

Financial support

No financial support was received to produce this manuscript.

Conflicts of interest

The authors declare no conflicts of interest that pertain to this work.

Please refer to the accompanying ICMJE disclosure forms for further details.

Authors' contribution

Study design: AG, CR, GR, EG, EJ. Clinical data collection: AG, CR, GR, MA, TA, PB, DD, EDM, BH, BR, MR, XS, GM. Experimental data collection: ML, TF. Pathology data collection and review: AM, CM. Writing of original draft: AG, CR. Extensive review: EG, EJ. Review of the final manuscript and approval of the submitted version: all authors.

Data availability

All data will be accessible upon request.

Acknowledgements

We thank the patients and their families, as well as all the clinicians involved in patient care, including all physicians of the Hepatology Department of Bicêtre Hospital (Dr Dalila Habes, Dr Oanez Ackermann, and Dr Alice Thebaut), Pr Stéphanie Franchi (Radiology, Bicêtre Hospital), Dr Philippe Durand (Intensive Care Unit, Bicêtre Hospital), Pr Branchereau and Pr Guérin (Surgery, Bicêtre Hospital), Dr Philippe Rouleau and Pr Catherine Baujard (Anesthesiology Department, Bicêtre Hospital), Pr. Catherine Guettier (Pathology Department, Bicêtre Hospital), Dr Teresa Kwon (Nephrology Department, Robert Debré Hospital), and Dr Anne Spraul (Biochemistry Department, Bicêtre Hospital). We also thank Mauricette Collado-Hilly and Brigitte Grosse (U1193 research unit) for their technical help. We are also grateful to AMFE (Association Maladie Foie Enfants, Malakof, France), Association 'Pour Louis 1000 Foie Merci' (Fournet-Luisans, France), and Fondation Rumsey-Cartier (Geneva, Switzerland) for their support. Graphical abstract was created with BioRender.

Supplementary data

Supplementary data to this article can be found online at https://doi.org/10.1016/j.jhepr.2026.101969.

References

Author names in bold designate shared co-first authorship

[1] Davit-Spraul A, Gonzales E, Baussan C, et al. Progressive familial intrahepatic cholestasis. Orphanet J Rare Dis 2009;4:1.

[2] Baker A, Kerkar N, Todorova L, et al. Systematic review of progressive familial intrahepatic cholestasis. Clin Res Hepatol Gastroenterol 2019;43:20–36.

[3] Davit-Spraul A, Fabre M, Branchereau S, et al. ATP8B1 and ABCB11 analysis in 62 children with normal gamma-glutamyl transferase progressive familial intrahepatic cholestasis (PFIC): phenotypic differences between PFIC1 and PFIC2 and natural history. Hepatology 2010;51:1645–1655.

[4] Jansen PLM, Strautnieks SS, Jacquemin E, et al. Hepatocanalicular bile salt export pump deficiency in patients with progressive familial intrahepatic cholestasis. Gastroenterology 1999;117:1370–1379.

[5] Van Wessel DBE, Thompson RJ, Gonzales E, et al. Genotype correlates with the natural history of severe bile salt export pump deficiency. J Hepatol 2020;73:84–93.

[6] Knisely AS, Strautnieks SS, Meier Y, et al. Hepatocellular carcinoma in ten children under five years of age with bile salt export pump deficiency. Hepatology 2006;44:478–486.

[7] Felzen A, Van Wessel DBE, Gonzales E, et al. Genotype-phenotype relationships of truncating mutations, p.E297G and p.D482G in bile salt export pump deficiency. JHEP Rep 2023;5:100626.

[8] Strautnieks SS, Byrne JA, Pawlikowska L, et al. Severe bile salt export pump deficiency: 82 different ABCB11 mutations in 109 families. Gastroenterology 2008;134:1203–1214.e8.

[9] Loomes KM, Squires RH, Kelly D, et al. Maralixibat for the treatment of PFIC: long-term, IBAT inhibition in an open-label, phase 2 study. Hepatol Commun 2022;6:2379–2390.
[10] Mehl A, Bohorquez H, Serrano M-S, et al. Liver transplantation and the management of progressive familial intrahepatic cholestasis in children. World J Transpl 2016;6:278.
[11] Sturm E, Latta A, Drews D, et al. Case report: recurrence of progressive familial intrahepatic cholestasis (pfic) in a living related liver transplant [Abstract]. Hepatol 1995;22:207.
[12] **Jara P**, **Hierro L**, **Martínez-Fernández P**, et al. Recurrence of bile salt export pump deficiency after liver transplantation. N Engl J Med 2009;361:1359–1367.
[13] Keitel V, Burdelski M, Vojnisek Z, et al. De novo bile salt transporter antibodies as a possible cause of recurrent graft failure after liver transplantation: a novel mechanism of cholestasis. Hepatology 2009;50:510–517.
[14] **Maggiore G**, **Gonzales E**, Sciveres M, et al. Relapsing features of bile salt export pump deficiency after liver transplantation in two patients with progressive familial intrahepatic cholestasis type 2. J Hepatol 2010;53:981–986.
[15] Siebold L, Dick AAS, Thompson R, et al. Recurrent low gamma-glutamyl Transpeptidase cholestasis following liver transplantation for bile salt export pump (BSEP) disease (posttransplant recurrent BSEP disease). Liver Transpl 2010;16:856–863.
[16] Lin HC, Alvarez L, Laroche G, et al. Rituximab as therapy for the recurrence of bile salt export pump deficiency after liver transplantation: rituximab for recurrence of BSEP deficiency after lt. Liver Transpl 2013;19:1403–1410.
[17] Grammatikopoulos T, Knisely AS, Dhawan A, et al. Anti-CD20 monoclonal antibody therapy in functional bile salt export pump deficiency after liver transplantation. J Pediatr Gastroenterol Nutr 2015;60:e50–e53.
[18] **Kubitz R**, **Dröge C**, Kluge S, et al. Autoimmune BSEP disease: disease recurrence after liver transplantation for progressive familial intrahepatic cholestasis. Clin Rev Allergy Immunol 2015;48:273–284.
[19] Stindt J, Kluge S, Dröge C, et al. Bile salt export pump-reactive antibodies form a polyclonal, multi-inhibitory response in antibody-induced bile salt export pump deficiency. Hepatology 2016;63:524–537.
[20] Kubitz R, Dröge C, Kluge S, et al. High affinity anti-BSEP antibodies after liver transplantation for PFIC-2 – successful treatment with immunoadsorption and B-cell depletion. Pediatr Transpl 2016;20:987–993.
[21] Masahata K, Uehara S, Ibuka S, et al. Recurrence of progressive familial intrahepatic cholestasis type 2 phenotype after living-donor liver transplantation: a case report. Transpl Proc 2016;48:3156–3162.
[22] Prusinskas B, Kathemann S, Pilic D, et al. Cholestasis after pediatric liver transplantation–recurrence of a progressive familial intrahepatic cholestasis phenotype as a rare differential diagnosis: a case report. Transpl Proc 2017;49:1628–1633.
[23] Patel KR, Harpavat S, Finegold M, et al. Post-transplant recurrent bile salt export pump disease: a form of antibody-mediated graft dysfunction and utilization of C4d. J Pediatr Gastroenterol Nutr 2017;65:364–369.
[24] Brinkert F, Pukite I, Krebs-Schmitt D, et al. Allogeneic haematopoietic stem cell transplantation eliminates alloreactive inhibitory antibodies after liver transplantation for bile salt export pump deficiency. J Hepatol 2018;69:961–965.
[25] **Krebs-Schmitt D**, **Briem-Richter A**, Brinkert F, et al. Alloimmunity and cholestasis after liver transplantation in children with progressive familial intrahepatic cholestasis. J Pediatr Gastroenterol Nutr 2019;68:169–174.
[26] Quintero J, Juamperez J, Gonzales E, et al. Successful treatment with rituximab and immunoadsorption for an auto-antibody induced bile salt export pump deficiency in a liver transplanted patient. Pediatr Gastroenterol Hepatol Nutr 2020;23:174.
[27] Romeres SGB, Trevizoli NDC, Oliveira CAMD, et al. Progressive familial intrahepatic cholestasis type 2 and recurrence after liver transplantation: a case report. Transpl Proc 2022;54:1370–1375.
[28] Wischlen E, Laverdure N, Erard D, et al. Iterative antibody-induced bile salt export pump deficiency after successive liver transplantations successfully treated with plasmapheresis and rituximab. Clin Res Hepatol Gastroenterol 2023;47:102139.
[29] Bak YG, Choi HJ, Kim YE, et al. Successful use of bortezomib for recurrent progressive familial intrahepatic cholestasis type II after liver transplantation: a pediatric case with a 9-year follow-up. Pediatr Gastroenterol Hepatol Nutr 2024;27:71.
[30] Grotra R, Das P, Yadav R, et al. Bortezomib therapy in autoimmune-BSEP disease after liver transplantation: case report with review of the literature. J Clin Exp Hepatol 2026;16:103418.
[31] Dröge C, Bonus M, Baumann U, et al. Sequencing of FIC1, BSEP and MDR3 in a large cohort of patients with cholestasis revealed a high number of different genetic variants. J Hepatol 2017;67:1253–1264.
[32] Gonzales E, Grosse B, Schuller B, et al. Targeted pharmacotherapy in progressive familial intrahepatic cholestasis type 2: evidence for improvement of cholestasis with 4-phenylbutyrate. Hepatology 2015;62:558–566.
[33] Kavallar AM, Mayerhofer C, Aldrian D, et al. Management and outcomes after liver transplantation for progressive familial intrahepatic cholestasis: a systematic review and meta-analysis. Hepatol Commun 2023;7:e0286.
[34] **Stindt J**, **Dröge C**, Lainka E, et al. Cell-based BSEP trans-inhibition: a novel, non-invasive test for diagnosis of antibody-induced BSEP deficiency. JHEP Rep 2023;5:100690.
[35] Noé J, Stieger B, Meier PJ. Functional expression of the canalicular bile salt export pump of human liver. Gastroenterology 2002;123:1659–1666.
[36] Huang E, Maldonado AQ, Kjellman C, et al. Imlifidase for the treatment of anti-HLA antibody-mediated processes in kidney transplantation. Am J Transpl 2022;22:691–697.
[37] Keitel V, Kreissl MC, Goetze T, et al. CAR T-cell therapy induces remission in multiorgan IgG4-related disease with hepatobiliary involvement. J Hepatol 2025:S0168827825027114.
[38] **Zhang Z**, **Xu Q**, Huang L. B cell depletion therapies in autoimmune diseases: monoclonal antibodies or chimeric antigen receptor-based therapy? Front Immunol 2023;14:1126421.
[39] Venturi C, Sempoux C, Bueno J, et al. Novel histologic scoring system for long-term allograft fibrosis after liver transplantation in children. Am J Transpl 2012;12:2986–2996.
[40] Demetris AJ, Bellamy C, Hübscher SG, et al. 2016 comprehensive update of the Banff working group on liver allograft Pathology: introduction of antibody-mediated rejection. Am J Transpl 2016;16:2816–2835.

Keywords: BSEP deficiency; PFIC2; Alloimmunization; Liver transplantation.
Received 16 March 2026; received in revised form 17 July 2026; accepted 21 July 2026; Available online 25 July 2026

Journal of Hepatology, Volume *8*

Supplemental information

Post-transplant alloimmune liver disease in bile salt export pump deficiency: A multicenter study

Antoine Gardin, Chiara Rubino, Alice Michaut, Géraldine Résin, Marion Almes, Teresa Antonini, Patrick Borentain, Dominique Debray, Eleonora De Martin, Bogdan Hermeziu, Bertrand Roquelaure, Mathias Ruiz, Xavier Stéphenne, Martine Lapalus, Giuseppe Maggiore, Thomas Falguières, Charlotte Mussini, Emmanuel Gonzales, and Emmanuel Jacquemin

Post-transplant alloimmune liver disease in bile salt export pump deficiency: A multicenter study

Antoine Gardin, Chiara Rubino, Alice Michaut, Géraldine Résin, Marion Almes, Teresa Antonini, Patrick Borentain, Dominique Debray, Eleonora De Martin, Bogdan Hermeziu, Bertrand Roquelaure, Mathias Ruiz, Xavier Stéphenne, Martine Lapalus, Giuseppe Maggiore, Thomas Falguières, Charlotte Mussini, Emmanuel Gonzales, Emmanuel Jacquemin

Table of contents

Supplementary Methods....................3
Fig. S1....................4
Fig. S2....................5
Fig. S3....................6
Fig. S4....................7
Table S1....................9
Table S2....................11
Table S3....................15
Table S4....................18
Table S5....................20

Supplementary Methods

Analysis of the epitope specificity of the anti-BSEP antibodies in patient serum.

In order to identify the antigenic specificity of anti-BSEP antibodies, an immunogenicity profile of human BSEP (NP_003733.2; 1321 amino-acids) was established by Genecust (Boynes, France). In silico analysis based on the secondary structure of BSEP and the hydrophobicity profile generated with HMMTop (http://www.enzim.hu/hmmtop/) highlighted three peptide sequences with potential interest in the N-terminal region (amino-acids 21-35), the first extracellular loop (amino-acids 115-129) and the C-terminal region of the protein (amino-acids 1297-1312). Thus, three soluble peptides overlapping these potentially immunogenic regions (shown in bold in the following sequences) were designed: (1) KKFGEENDGF**ESDKSYNNDKKSRLQ**DEKKGDGVRV (N-terminal or B0 peptide, amino-acids 11-45); (2) LIFGTMTDVFIDYDVELQEL QIPGKACVNNTIVW**TNSSLNQNMTNGTRC**GLLNIESEMIKFASYYAG (B1 peptide, amino-acids 81-147); (3) **VMAQGVVIEKGTHEEL MAQKGAYYK**LVTTGSPIS (C-terminal or B2 peptide, amino-acids 1288-1321). The three purified peptides were reconstituted at a concentration of 1 µM in pure water, aliquoted and stored at -80°C until further use for ELISA. BSEP soluble peptides (B0, B1 and B2) were diluted at 1 µg/mL in the isotonic working buffer (25 mM Tris-HCl pH 7.4, 137 mM NaCl, 5.2 mM KCl) and adsorbed in wells (100 ng per well) of high binding 96-well ELISA plates Microlon 600 (Greiner Bio-One). After overnight incubation at 4°C and washes with the same buffer, samples were saturated 2 hours at room temperature in Tris-buffered saline containing 0.1% Tween 20 and 0.5% bovine serum albumin (Sigma-Aldrich Chimie). Then serum samples were sequentially incubated with 1:200 to 1:6400 serial dilutions of AIBD or control (biliary atresia) patients' sera (45 min at room temperature) and alkaline phosphatase-coupled anti-human secondary antibodies (30 min at room temperature). Then, 50 µL of alkaline phosphatase yellow (p-nitrophenylphosphate) liquid substrate for ELISA (Sigma-Aldrich Chimie) was added in each well and the colorimetric reaction was stopped by the addition of 50 µL of 0.1 M EDTA. Absorbance at 405 nm in the linear range was read using a Wallac 1420 Victor2 microplate reader (Perkin Elmer).

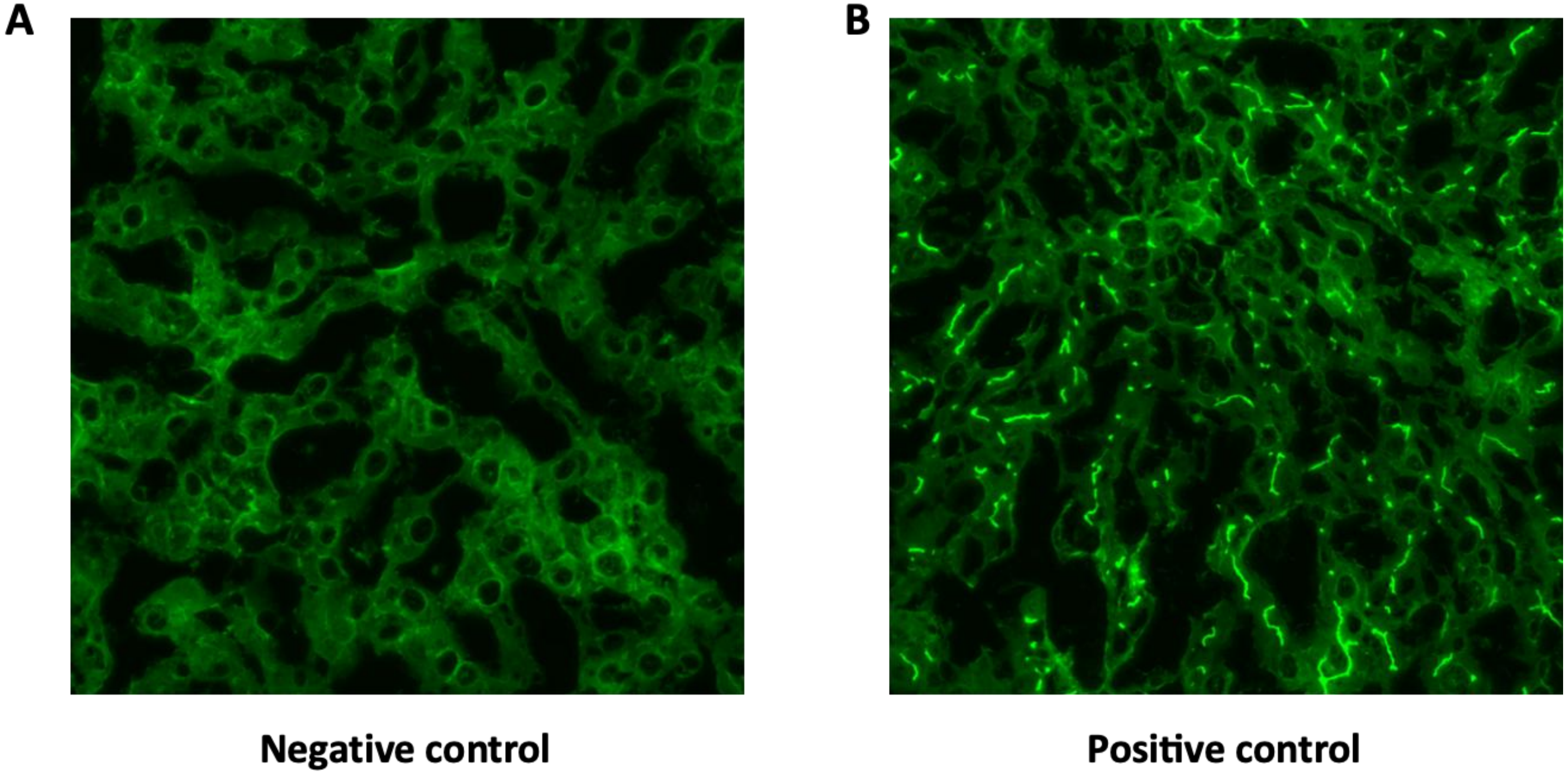

Fig. S1. Indirect immunofluorescence of cryosections of normal human liver incubated with patient serum samples to screen for anti-BSEP antibodies. A: negative control (magnification x400) showing the absence of immunoglobulin G deposit at the canalicular membrane of hepatocytes. B: positive control (magnification x400) showing immunoglobulin G deposits at the canalicular membrane of hepatocytes.

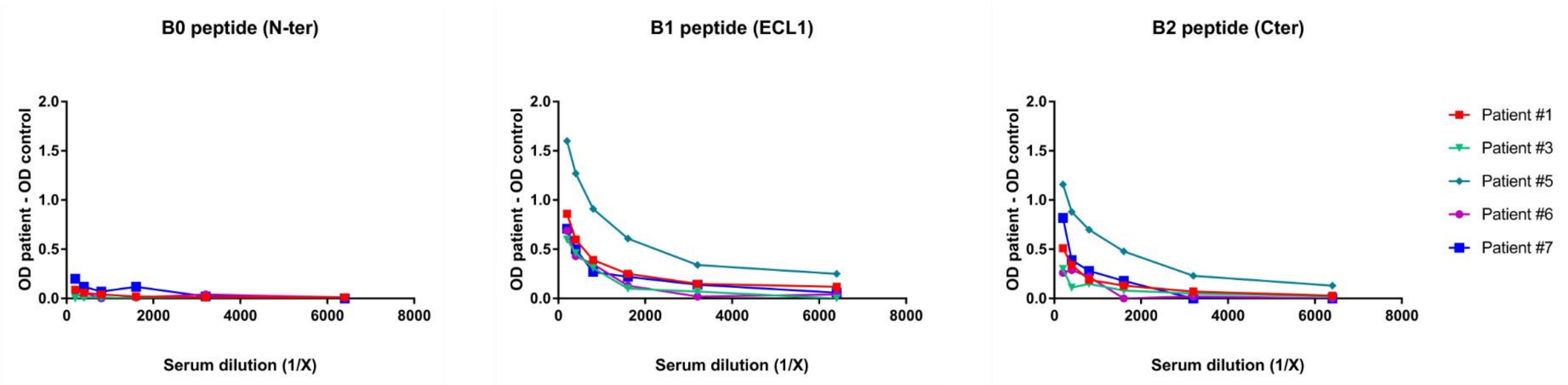

Fig. S2. **Analysis of the epitope specificity of the anti-BSEP antibody identified in serum of patients with AIBD**. ELISA performed using serum of patients with AIBD and three different peptides designed from the BSEP protein, expressed as the difference of optical density (OD) calculated between the tested (patient serum) and control (serum from biliary atresia patient) sera. The three peptides are named B0 (N-terminus), B1 (first extracellular loop/ECL1), B2 (C-terminus). Serial dilutions of the sera from 1:200 to 1:6400 were used.

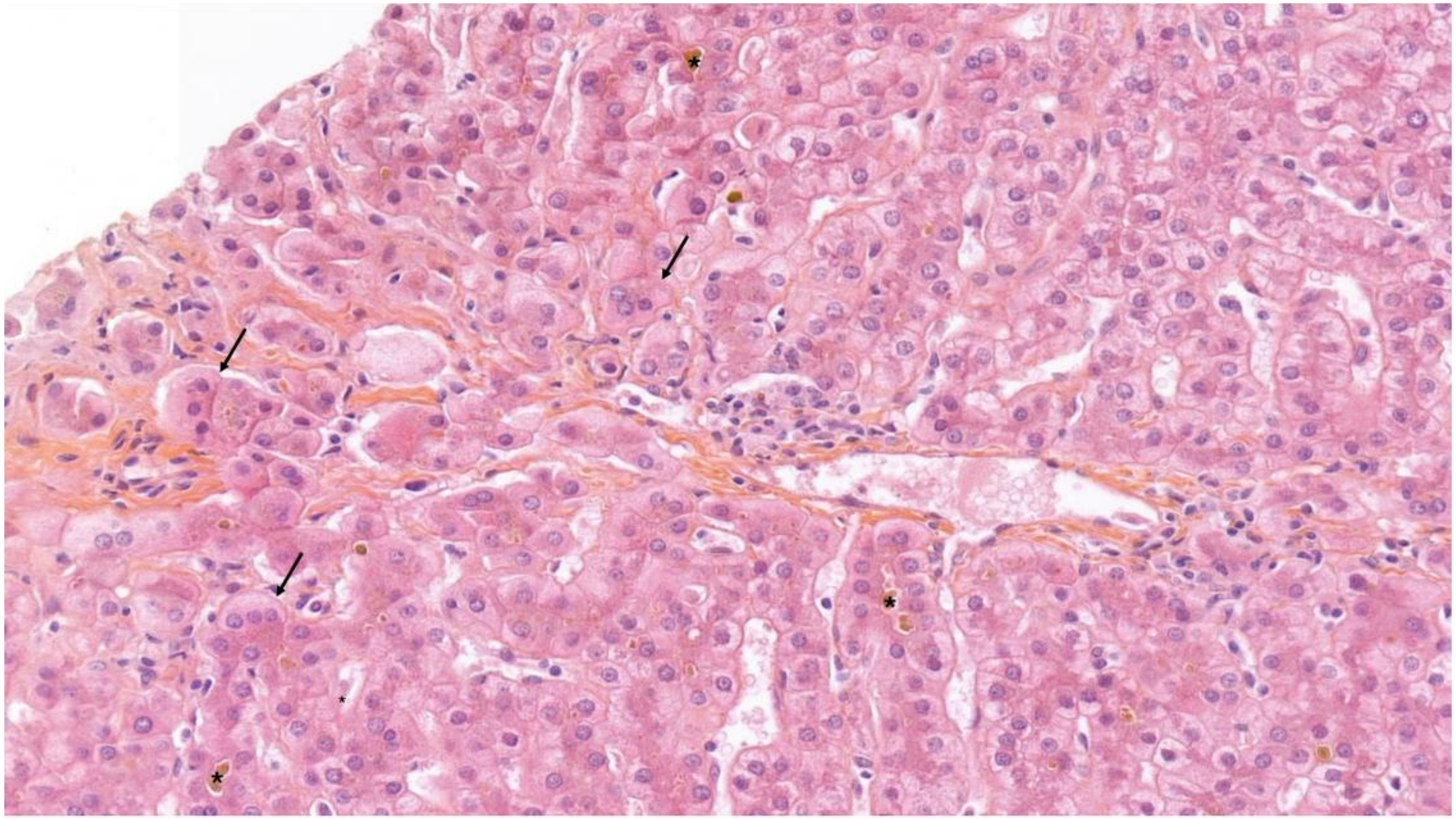

Fig. S3. Histological features of AIBD observed on liver biopsies. HES staining of biopsy of patient #1 (at relapse) recapitulating the cardinal features of AIBD, namely canalicular cholestasis (*) and giant transformation of hepatocytes (arrows). Magnification x 20.

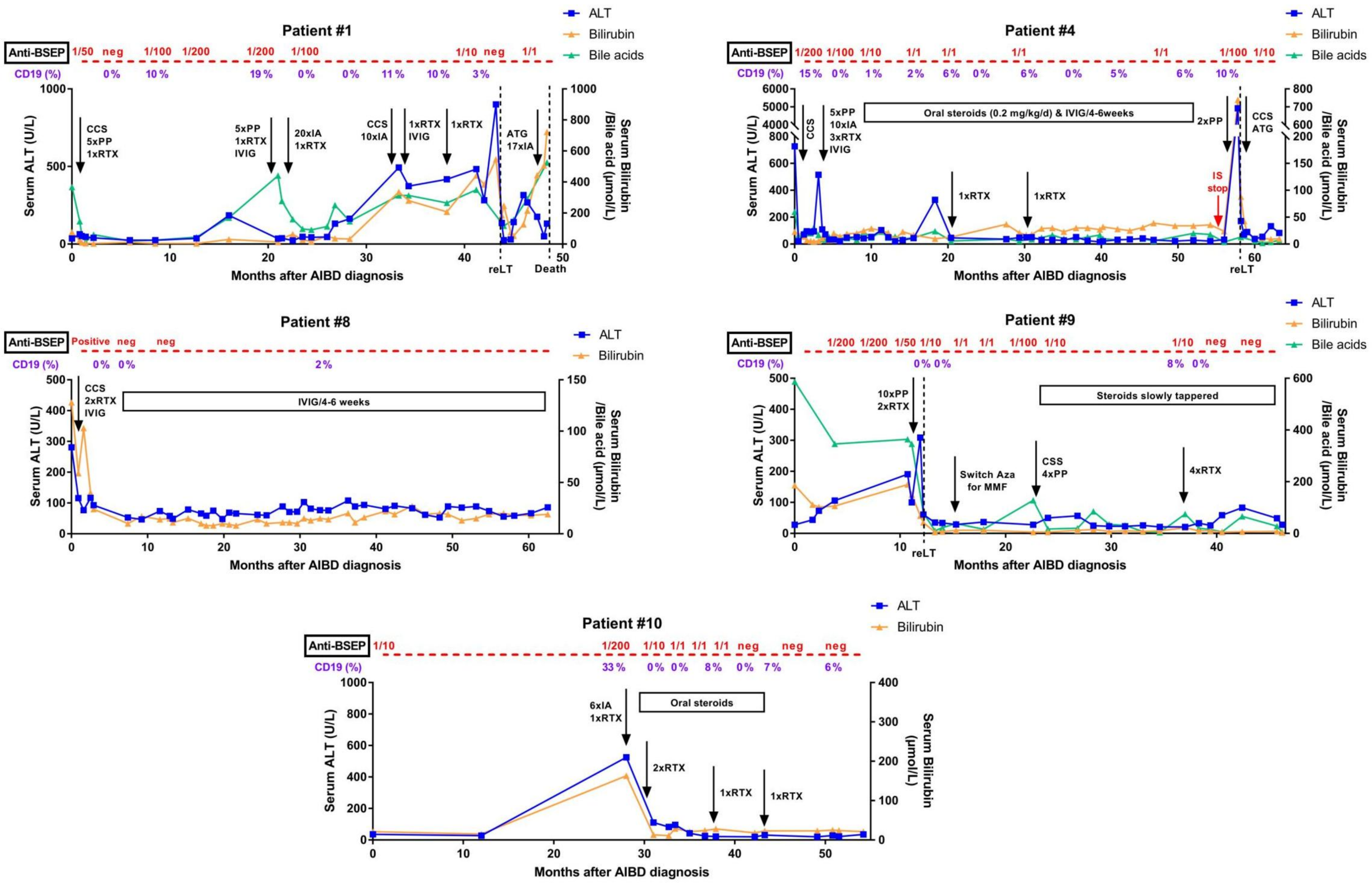

Fig. S4. Treatment and evolution of laboratory parameters and anti-BSEP antibody titers for patients #1, #4, #8, #9 and #10. Quantification of circulating CD19+ B-cells in plasma (% of total lymphocyte count) is shown. *ALT: alanine aminotransferase activity in serum; Anti-BSEP: anti-bile*

salt export pump antibody titers; ATG: anti-thymoglobulin; Aza: azathioprine; CCS: corticosteroids pulse; IA: immunoadsorption; IS: immunosuppressive regimen; IVIg: intravenous immunoglobulin; LT: liver transplantation; MMF: mycophenolate mofetil; neg: negative; PP: plasmapheresis; RTX: rituximab.

Table S1. Main characteristics of the 10 patients with severe BSEP deficiency who developed AIBD after liver transplantation.

Patient	Sex	*ABCB11* Variants	Age at LT in y (y of LT)	Indication for LT	Type of donor	BSEP staining on native liver	IS regimen	Complications after LT (interval after LT)	Treatment of complications
**#1**	M	c.3400 C>T (p.Gln1134*) homozygous	5 (2010)	Cirrhosis, LF	Deceased	Negative	Basiliximab, tacrolimus	TCMR (12d)	Steroids
								TCMR (0.5y)	Steroids, MMF
**#2**	M	c.1642_1644delTTT (p.Phe548del, in frame); c.3722del (p.Pro1091Leufs*6)	12 (2003)	Pruritus	Deceased	NA	NA	BA leakage (NA)	Surgery
								TCMR (NA)	Steroids
**#3[£]**	F	c.301delCA (p.Gln101Aspfs*8); c.2944G>A (p.Gly982Arg)	9 (1990)	Cholestasis and pruritus	Deceased	Negative	Cyclosporine, steroids	None	None
**#4**	F	c.1243C>T (p.Arg415*) homozygous	9.7 (2014)	Cirrhosis, Pruritus, dysplastic nodule	Deceased	Negative	Basiliximab, tacrolimus	BA stenosis (13d)	Surgery
								CMV infection (15d)	Ganciclovir
								TCMR (23d)	Steroids
								Plasma cell-rich rejection (1y)	Steroids, MMF
**#5[£]**	M	c.3268C>T (p.Arg1090*) homozygous	4.4 (1996)	Cirrhosis, LF	Living	Negative	Cyclosporine, azathioprine, steroids	TCMR (9d)	Steroids
								BA stenosis (14d)	Interventional radiology, Surgery
**#6**	M	Homozygous 17655bp deletion within *ABCB11*	3.9 (1998)	Cirrhosis, LF	Deceased	Negative	Cyclosporine, azathioprine, steroids	TCMR (7d)	Steroids
								Renal artery stenosis (0.3y)	Surgery
								TCMR (0.5y)	Steroids
								TCMR (2y)	Steroids
								Chronic rejection (3y)	Switch IS for tacrolimus and MMF

								Iterative rejection episodes (4-6y)	Tacrolimus dose increase; steroids; MMF interruption
								CMV hepatitis (5y)	Transient IS reduction
								BA stricture	UDCA
**#7[£]**	M	c.77-19T>A homozygous (p.Tyr26Ilsfs*7). Abnormal splicing.	2.8 (1989)	Cirrhosis, Pruritus	Deceased	Negative	Cyclosporine, steroids	TCMR (23d)	Steroids
								BA stenosis (30d)	Surgery
**#8**	M	c.1062T>A (p.Tyr354*) homozygous	2.3 (2012)	Cirrhosis, HCC	Deceased	Negative	Basiliximab, tacrolimus	TCMR (14d)	Steroids
								TCMR (30d)	Steroids, MMF
								BA stricture	Interventional radiology
**#9**	F	c.1101_1102delAG (p.Val368Argfs*27) ; del 2q24.3-q31.1 encompassing *ABCB11*	2.7 (2019)	Pruritus	Deceased	Negative	Basiliximab, tacrolimus	Plasma-cell rich rejection (0.3y)	Steroids, azathioprine
**#10**	M	c.1062T>A (p.Tyr354*) homozygous	5.7 (2008)	Cirrhosis, cholestasis	Deceased	NA	Basiliximab, tacrolimus, steroids	Infected ascites (30d)	Antibiotics, surgery

AIBD: alloimmune anti-BSEP liver disease; BA: biliary anastomosis; BSEP: bile salt export pump; CMV: cytomegalovirus; d: days; F: female; HCC: hepatocellular carcinoma; IS: immunosuppression; LF: liver failure; LT: liver transplantation; M: male; MMF: Mycophenolate mofetil; NA: not available; TCMR: T-cell mediated rejection; UDCA: ursodeoxycholic acid; y: year. [£]indicates retrospectively-diagnosed patients.

Table S2. Main characteristics of 25 patients with severe BSEP deficiency who did not develop AIBD.

Patient	Sex	*ABCB11* Variants	Age at LT in y (y of LT)	Indication for LT	Type of donor	BSEP staining on native liver	IS regimen	Complications (interval after LT)	Treatment of complications	Last follow-up (interval after LT)
**#11[#]**	M	c.1387A>C (p.Thr463Pro) homozygous	0.8 (2020)	LF, HCC	Deceased	Negative	Basiliximab, tacrolimus, MMF IVIg for 6 months post-LT	TCMR (10d)	Steroids	Alive (4.2y)
								BA stricture (30d)	UDCA, Interventional radiology, surgery	
**#12[#]**	F	c.3628A>C (p.Thr1210Pro) homozygous	16 (2016)	Pruritus	Deceased	Negative	Basiliximab, tacrolimus	TCMR & Plasma-cell rich rejection (4.5y)	Steroids MMF initiation	Alive (7.2y)
**#13**	F	c.2300C>G (p.Thr767Arg) homozygous	6.9 (2016)	Cirrhosis, HPS, Pruritus	Deceased	Positive	Basiliximab, tacrolimus, steroids, MMF	Bleeding (1d) & Infection (3-20d)	Multiple surgery	Alive (7.1y)
								Ischemic cholangiopathy (30d)	UDCA	
**#14**	F	c.1308+2T>A; c.1408C>T (p.Arg470*)	2.0 (2009)	Cirrhosis, LF, HCC	Deceased	Negative	Basiliximab, tacrolimus	TCMR (12d)	Steroids	Death (14y) HCC recurrence
								EBV-related tonsillar PTLD (0.3y)	Rituximab, tonsillectomy	
								HCC recurrence (13y)	Surgery Chemotherapy	
**#15**	M	c.1062T>A (p.Tyr354*) ; c.2944G>A (p.Gly982Arg)	4 (2008)	Cirrhosis, LF, HCC	Deceased	Negative	Basiliximab, tacrolimus	TCMR (19d)	Steroids	Alive (14.7y)
								Necrosis of segment IV	Surgery	
								Inferior vena cava stenosis (45d)	Interventional radiology	
**#16***	F	c.3181_3184delATCA (p.Ile1061ValfsTer35); c.1259T>C p.(Ile420Thr)	12.4 (2015)	Pruritus	Deceased	Negative	Basiliximab, tacrolimus	TCMR (13d)	Steroids	Alive (7.9y)
**#17***	M	c.3181_3184delATCA (p.Ile1061ValfsTer35); c.1259T>C (p.Ile420Thr)	0.4 (1993)	Cirrhosis, LF	Deceased	Negative	Cyclosporine, azathioprine, steroids	TCMR (6d)	Steroids	Alive (24.4y)
								TCMR (30d)	Steroids	
								Chronic Hepatitis B infection (0.7y)	Anti-viral treatment	
								EBV-related PTLD		

**#18**	M	c.890A>G (p.Glu297Gly); c.1826_1827insCA (p.Ile610Glyfs*45)	7.7 (2002)	Pruritus	Deceased	Negative	Cyclosporine, azathioprine, steroids	TCMR (10y)	Switch from azathioprine to MMF	Alive (22.2y)
**#19**	F	c.1062 T>A (p.Tyr354*) homozygous	3.5 (2017)	Pruritus	Deceased	Negative	Basiliximab, tacrolimus, MMF, steroids	EBV-related PTLD	Rituximab	Alive (6.2y)
**#20**	F	c.3432C>A (p.Ser1144Arg); c.611+4A>G	8.5 (1990)	Cirrhosis, LF	Deceased	Negative	Cyclosporine, azathioprine, steroids	BA stenosis (5d)	UDCA, Interventional radiology	Alive (33.7y) Pregnancy
**#21**	M	c.3691 C>T (p.Arg1231Trp); c.1582delA (p.Ils528*)	1.7 (2007)	Cirrhosis	Deceased	NA	Basiliximab, tacrolimus	TCMR (60d)	Steroids	Alive (15.3y)
								EBV-related PTLD (2y)	Tonsillectomy, Rituximab	
								TCMR (12y)	Steroids	
								TCMR (15y)	Steroids, tacrolimus dose increase	
**#22**	F	c.890 A>G (p.Glu297Gly); c.1435-17_1450dup (p.His484Argfs*5)	9.4 (2002)	Cirrhosis; Pruritus	Deceased	Negative	Tacrolimus, steroids	BA stenosis	UDCA, Interventional radiology	Alive (22.1y) Pregnancy
								Hepatic artery stenosis	Surveillance	
**#23**	M	c.3213 +4 A>G; c.3457 C>T (p.Arg1153Cys)	12.4 (2008)	Cirrhosis; LF, HCC	Deceased	Negative	Basiliximab, tacrolimus	BA leakage (1d)	Surgery	Alive (15.4y)
								TCMR (10d)	Steroids	
**#24**	M	c.390-1G>A; c.3213+5G>A	8.2 (2020)	Pruritus	Living	Negative	Basiliximab, tacrolimus, MMF, IVI for 6 months post-LT	Mild Budd-Chiari syndrome	Heparin	Alive (4.3y)
**#25**	M	c.545T>A (p.Ile182Lys); c.3213+5G>A	6.3 (2000)	Cirrhosis	Deceased	Negative	Tacrolimus, steroids	TCMR (2d)	Steroids	Alive (14y)
								Digestive Burkitt lymphoma (8y)	Transient IS withdrawal Chemotherapy	
								BA stricture (11y)	UDCA, Surgery	
**#26**	M	c.3123 T>A (p.Tyr1041*); c.3008G>A (p.Gly1003Glu)	14.6 (2009)	Pruritus	Deceased	Negative	Basiliximab, tacrolimus	Hepatic artery stenosis	Spontaneous resolution	Alive (12.6y)

**#27[$]**	M	c.1826_1827dupCA (p.Ile610Glnfs*45) homozygous	12 (2016)	Cirrhosis, HPS, Pruritus	Deceased	NA	Basiliximab, tacrolimus, MMF, steroids	Bleeding & BA leak (2d)	Surgery	Alive (6.1y)
								TCMR (4y & 5y)	Steroids	
**#28[$]**	F	c.1826_1827dupCA (p.Ile610Glnfs*45) homozygous	6.0 (2016)	Cirrhosis	Deceased	Negative	Basiliximab, tacrolimus, MMF, steroids	TCMR (0.3y & 1y)	Steroids	Alive (6.1y)
								TCMR (4y)	MMF dose increase	
								TCMR (5y)	Tacrolimus dose increase	
								BA stricture	UDCA	
**#29[£]**	F	c.3382C>T (p.Arg1128Cys) homozygous	7.5 (2001)	Cirrhosis, LF	Deceased	Negative	Tacrolimus, steroids	TCMR (14d)	Steroids	Alive (22.8y) 2 pregnancies
								TCMR (60d)	Steroids, azathioprine	
**#30[£]**	F	c.3382C>T (p.Arg1128Cys) homozygous	8.0 (2008)	Cirrhosis, LF	Deceased	Negative	Basiliximab, tacrolimus	TCMR (0.3y)	Steroids	**reLT** (9.4y) **Alive** (15.6y, 6.2y post reLT) Pregnancy
								TCMR (4y)	Steroids, MMF	
								BA strictures	UDCA, Interventional radiology	
								Chronic rejection (6y)	reLT	
**#31**	F	c.713G>T (p.Gly238Val) homozygous	2.6 (1994)	LF	Deceased	Negative	Cyclosporine, steroids, Azathioprine	TCMR (7d)	Steroids, azathioprine	**Listed for reLT** (persistent portal hypertension) Alive (15.9y)
								Pneumocystis infection		
								Chronic rejection (10y)	Switch Azathioprine for MMF	
								Portal vein thrombosis	Meso-Rex bypass	
**#32**	F	c.1826_1827dupCA (p.Ile610Glnfs*45) homozygous	2.8 (2014)	Pruritus	Living	NA	Basiliximab, cyclosporine	None	None	Alive (9.3y)
**#33**	F	c.3400C>T (p.Gln1134*) homozygous	4.0 (2012)	Pruritus, HCC	Deceased	NA	Basiliximab, tacrolimus	Budd-Chiari (5y)	Interventional radiology	Alive (11.1y)
								Chronic rejection (10y)	Azathioprine	
**#34[#]**	M	c.409G>A (p.Glu137Lys); c.1418_1419ins18pb	7 (2021)	Pruritus	Living	Focally positive	Basiliximab, tacrolimus	BA stricture	UDCA	Alive (2y)
**#35[#]**	F	c.677C>T (p.Ser226Leu) homozygous	17.3 (2023)	Cirrhosis, LF	Deceased	Negative	Tacrolimus, MMF, Steroids	TCMR (4d)	Steroids	Alive (1.1y)
								BA leak (4d)	Surgery	

*AIBD: alloimmune anti-BSEP liver disease; BSEP: bile salt export pump; d: days; EBV: Epstein Barr Virus; F: female; IS: immunosuppression; HPS: hepatopulmonary syndrome; IVIg: intravenous immunoglobulin; LF: liver failure ; LT: liver transplantation; M: male; MMF: Mycophenolate mofetil; NA: not available; PTLD: post-transplant lymphoproliferative disorder; reLT: liver retransplantation; TCMR: T-cell mediated rejection; UDCA: ursodeoxycholic acid; y: year. *, [$], [£] denotes siblings. [#]All these four patients received 4-phenylbutyrate pre-LT as targeted pharmacotherapy.*

Table S3. Liver biopsy in the follow-up of patients with AIBD.

Patient	Time of biopsy (time after AIBD diagnosis)	Histology	Fibrosis LAFSc score	Acute TCMR (BANFF)	BSEP immunostaining	C4d immunostaining BANFF score	AMR
#1	**Initial episode**	Severe canalicular cholestasis; rare giant hepatocytes<br>Mild centrilobular and portal inflammation; biliary obstruction	4 (2+0+2)	No	**Reduced canalicular expression**	**Positive (canalicular, lateral membrane)**<br>BANFF: 0	No
	**Relapse** (1.7y)	Canalicular cholestasis; increase of portal inflammation, persistence of biliary obstruction; hepatocyte clarification	5 (2+1+2)	No	**Reduced canalicular expression**	**Positive (canalicular)**<br>BANFF: 0	No
	**Relapse** (1.9y)	Canalicular cholestasis; several giant hepatocytes; mild centrilobular and portal inflammation	3 (2+0+1)	No	**Reduced canalicular expression**	**Positive (canalicular)**<br>BANFF: 0	No
	**Relapse** (2.7y)	Cholestatic cirrhosis; several giant hepatocytes with focal ballooning; moderate centrilobular and portal inflammation; biliary obstructions	9 (3+3+3)	No	**Reduced canalicular expression**	**Positive (canalicular, lateral membrane)**<br>BANFF: 0	No
	**Explanted graft** (3.6y)	Cholestatic cirrhosis; several giant hepatocytes with focal ballooning; moderate centrilobular and portal inflammation; biliary obstruction	9 (3+3+3)	No	Normal canalicular expression	**Positive (canalicular)**<br>BANFF: 0	No
	**Relapse** (0.1y after reLT)	Moderate canalicular cholestasis; rare giant hepatocytes; mild portal inflammation; biliary obstructions; rare ballooned hepatocytes	3 (1+1+1)	Yes (4: 1+1+2)	**Reduced canalicular expression**	**Positive (canalicular)**<br>BANFF: 0	No
	**Relapse** (0.2y after reLT)	Increase of portal fibrosis; neutrophil inflammation and intraductular bile plugs (sepsis with possible biliary obstruction)	3 (2+0+1)	Yes (7: 2+3+2)	Normal canalicular expression	**Positive (canalicular)**<br>BANFF: 0	No
	**Relapse** (0.3y after reLT)	Increase of fibrosis; canalicular cholestasis with several giant hepatocytes; lobular hepatitis	6 (2+2+2)	Yes (4: 1+2+1)	Normal canalicular expression	**Positive (canalicular)**<br>BANFF: 0	No
#2	**Initial episode**<br>*Explanted liver*[§]	Severe cholestasis, giant hepatocytes, moderate portal inflammation	7 (2+2+3)	No	Normal canalicular expression	NA	NA
	**Initial episode**[§] (0.5y after reLT)	Cholestasis; giant hepatocytes; ductopenia (chronic TCMR)	2 (0+1+1)	No	Normal canalicular expression	NA	No
	**Initial episode** (0.7y after reLT)	Cirrhosis; cholestasis; giant hepatocytes; ductopenia	9 (3+3+3)	NA	Normal canalicular expression	**Positive (canalicular)**	NA

**#3**	**Relapse**§ (14y)	Lobular cholestasis; giant hepatocytes; no biliary obstruction	NA	No	NA	NA	NA
**#4**	**Initial episode**	Lobular and portal hepatitis; rare giant hepatocytes	2 (1+1+0)	Yes (6: 3+1+2)	Normal canalicular expression	**Positive (canalicular)** BANFF: 0	No
	**Initial episode** (0.4y)	No cholestasis, some giant hepatocytes, Reduction of portal inflammation; increase in centrilobular inflammation	5 (1+2+2)	Yes (4: 1+0+3)	Normal canalicular expression	Positive (portal vein) BANFF score: 1+	No
	**Relapse** (4.8y) *Explanted liver*	Bridging hemorrhagic necrosis, canalicular cholestasis, giant hepatocytes, moderate portal inflammation	NA	No	Normal canalicular expression	**Positive (canalicular)** BANFF: 0	No
**#5**	**Initial episode**	Moderate portal and mild perivascular inflammation	2 (1+0+1)	No	Normal canalicular expression	NA	Yes (chronic)
	**Relapse** (10y) *Explanted liver*§	Biliary cirrhosis, chronic cholangitis, hepatocellular cholestasis with giant hepatocytes	NA	No	NA	NA	No
	**Protocol biopsy**§ (15y, 5y after reLT)	Mild lobular hepatitis with centrilobular necrosis No cholestasis. No giant hepatocytes.	NA	No	NA	NA	No
**#6**	**Initial episode**	Canalicular cholestasis; giant hepatocytes Moderate portal inflammation with ductular proliferation.	5 (3+2+0)	Yes (3: 2+0+1)	**Reduced canalicular expression**	Negative	No
	**Initial episode** (0.4y)	Canalicular cholestasis, giant hepatocytes. Moderate portal inflammation with ductular reaction	5 (3+2+0)	Yes (4: 2+1+1)	**Reduced canalicular expression**	Negative	No
	**Initial episode** (0.5y) *Explanted liver*	Cholestatic cirrhosis; several giant hepatocytes with lobular/bridging hepatocellular necrosis; biliary obstruction	9 (3+3+3)	No	Normal canalicular expression	Negative	No
	**Initial episode** (0.8y, 0.4y after reLT)	Mild cholestasis; focal lobular and portal hepatitis	3 (1+1+1)	No	Normal canalicular expression	Negative	No
	**Relapse** (9.7y)	Canalicular cholestasis; several giant hepatocytes	NA	NA	NA	NA	
**#7**	**Initial episode**§	Canalicular cholestasis, giant hepatocytes Slight centrilobular fibrosis	NA	NA	NA	NA	NA
**#8**	**Initial episode**	Canalicular cholestasis, giant hepatocytes Portal inflammation	3 (1+2+0)	Yes (5: 2+2+1)	**Reduced canalicular expression**	**Positive (canalicular)** BANFF: 0	No

**#9**	**Initial episode**[§]	Cholestasis with ductopenia and bile duct dystrophy	NA	NA	NA	NA	NA
	**Initial episode**[§] (1.2y, 0.1y after reLT)	Lymphocytic and eosinophilic inflammation; canalicular cholestasis	NA	Yes (5: 2+2+1)	NA	NA	NA
	**Initial episode**[§] (1.3y, 0.2y after reLT)	Mild portal fibrosis	4 (3+0+1)	NA	Normal canalicular expression	NA	NA
	**Relapse**[§] (1.9y, 0.8y after reLT)	Mild portal and lobular hepatitis	2 (1+1+0)	NA	NA	NA	NA
**#10**	**Initial episode** (at symptom onset)	Cholestasis; giant hepatocytes	NA	No	**Reduced canalicular expression**	**Positive (canalicular)**	No

LAFSc is expressed as the sum of a 0-3 score referring to portal tract, sinusoids and central vein fibrosis (total score: 0-9)[35]. TCMR was graded using BANFF score, expressed as the sum of a 0-3 score referring to portal tract inflammation, bile duct inflammatory lesions and venous endothelial inflammation (max 0-9)[36]. C4d staining was also staged according to the BANFF scoring system[36]. All biopsies were reviewed by a single pathologist of Bicêtre Hospital, unless indicated by the symbol "§". *AIBD: alloimmune anti-BSEP liver disease; AMR: antibody-mediated rejection; BSEP: bile salt export pump; C4d: complement component 4d; LAFSc: Liver allograft fibrosis score; LT: liver transplantation; NA: not available; reLT: liver retransplantation; TCMR: T cell–mediated rejection; y: year.*

Table S4. Histology analysis of liver biopsies performed in 11 patients without AIBD.

Patient	Interval after LT *(years)*	Histology	LAFSc	TCMR	BSEP immunostaining	C4d immunostaining BANFF score	AMR
**#11**	0.9y	No cholestasis, no giant hepatocytes. Biliary obstruction.	2 (1+0+1)	No	Normal canalicular expression	Negative	No
	2y	No cholestasis, no giant hepatocytes. Biliary obstruction.	4 (2+1+1)	No	Normal canalicular expression	Negative	No
	3.8y	No cholestasis, no giant hepatocytes. Biliary obstruction.	5 (3+1+1)	No	Normal canalicular expression	Negative	No
**#14**	10y	No cholestasis, no giant hepatocytes Portal fibrosis, biliary obstruction	4 (2+1+1)	No	**Reduced canalicular expression**	Negative	No
**#15**	10y	No cholestasis, no giant hepatocytes Chronic hepatitis, acute AMR	5 (3+1+1)	Undetermined TCMR	Normal canalicular expression	Positive (sinusoidal) BANFF: 1 **No canalicular staining**	Yes (acute)
**#16**	0.1y	No cholestasis, no biliary obstruction Acute TCMR	1 (0+0+1)	Yes (5: 2+1+2)	Normal canalicular expression	Negative	No
**#17**	8y	No cholestasis, no giant hepatocytes Cirrhosis with chronic hepatitis B	8 (3+3+2)	No	Normal canalicular expression	Negative	No
**#18**	16y	No cholestasis, no giant hepatocytes Central vein fibrosis (chronic AMR)	5 (2+2+1)	Yes (3)	Normal canalicular expression	Negative	Yes (chronic)
	20y	No cholestasis, no giant hepatocytes Central vein fibrosis	4 (1+1+2)	No	Normal canalicular expression	Negative	No
**#20**	10y	Normal morphology	1 (1+0+0)	No	Normal canalicular expression	Negative	No
**#23**	10y	No cholestasis, no giant hepatocytes Biliary obstruction	6 (3+2+1)	No	Normal canalicular expression	Negative	No
**#24**	0.1y	Mild cholestasis, rare giant hepatocytes Lobular hepatitis	1 (0+0+1)	Yes (2: 1+0+1)	Normal canalicular expression	Negative	No
**#30**	6.8y	Mild central vein fibrosis No cholestasis or giant hepatocytes.	1 (0+0+1)	No	Normal canalicular expression	Negative	No
	9.5y	**Explanted liver**: chronic TCMR, cirrhosis, intense cholestasis	9 (3+3+3)	No	Normal canalicular expression	Negative	No
	11.3y (1.9y after reLT)	Chronic TCMR. No cholestasis, no giant hepatocytes	2 (1+0+0)	Yes (3: 1+1+1)	Normal canalicular expression	Positive (sinusoidal) BANFF: 0 **No canalicular staining**	No
	15y (5.5y after reLT)	Cirrhosis, chronic TCMR No cholestasis	NA	Yes (3: 1+1+1)	Normal canalicular expression	Negative	No
**#31**	10y	No cholestasis, no giant hepatocytes Central vein fibrosis	4 (2+1+1)	No	Normal canalicular expression	Negative	Undetermined acute AMR

LAFSc is expressed as the sum of a 0-3 score referring to portal tract, sinusoids and central vein fibrosis (total score: 0-9)[35]. TCMR was graded using BANFF score, expressed as the sum of a 0-3 score referring to portal tract inflammation, bile duct inflammatory lesions and venous endothelial inflammation (max 0-9)[36]. C4d staining was also staged according to the BANFF scoring system[36]. All biopsies were reviewed by a single pathologist of Bicêtre Hospital, unless indicated by the symbol "§". *AIBD: alloimmune anti-BSEP liver disease; AMR: antibody-mediated rejection; BSEP: bile salt export pump; C4d: complement component 4d; LAFSc: Liver allograft fibrosis score; LT: liver transplantation; NA: not available; reLT: liver retransplantation; TCMR: T cell–mediated rejection; y: year.*

Table S5. Detailed outcome of patients with AIBD

Patient	Initial episodes and relapses (time after AIBD diagnosis)	AIBD treatment		Outcome		Last follow-up (time after AIBD diagnosis)
		**Intensification therapy**	**Maintenance therapy**	**Clinical outcome**	**Immunological outcome**	
#1	**Initial episode**	Pulse steroids<br>Rituximab x 1<br>Plasmapheresis x 5<br>IVIg x 4	Tacrolimus - MMF	Clinical remission	Negative anti-BSEP Ab	**Death** (4y)<br>Recurrence of AIBD
	**Relapse (1.7y)**<br>*Pruritus*<br>*Anti-BSEP Ab 1/200*	Pulse steroids<br>Plasmapheresis x 5<br>Rituximab x 4<br>IA x 47<br>IVIg<br>Anti-thymocyte globulin	-	**Persistent cholestasis**<br>Liver failure with **reLT**<br>(3.6y after AIBD diagnosis)	Lowering of anti-BSEP Ab titer from 1/200 to 1/1	
#2	**Initial episode**<br>(diagnosis 1y after reLT)	MMF<br>Plasmapheresis<br>Rituximab<br>IVIg	-	**Persistent cholestasis**<br>Liver failure	Persistent anti-BSEP Ab<br>(no titer available)	**Death** (0.6y)<br>Recurrence of AIBD
#3*	**Initial episode**	Cyclosporine and steroid dose increase	Cyclosporine<br>Steroid	Clinical remission	NA	**Death** (17y) while waiting for reLT
	**Relapse (14y)**<br>*Jaundice*<br>*Positive anti-BSEP Ab*	Cyclosporine and steroid dose increase<br>IVIg	-	**Persistent cholestasis**<br>Evolution toward cirrhosis	Persistent anti-BSEP Ab<br>(no titer available)	
#4	**Initial episode**	Pulse steroids<br>IVIg x 10<br>Rituximab x 3<br>Plasmapheresis x 5<br>IA x 10	Tacrolimus – MMF<br>Steroids 0.2 mg/kg/d<br>IVIg/months<br>+/- Rituximab	Clinical remission	Lowering of anti-BSEP Ab titer from 1/200 to 1/1	**Alive** (5.2y)<br>Clinical remission<br>0.4y after relapse
	**Relapse (4.8y)**<br>*IS interruption*<br>*Jaundice, liver failure*<br>*Anti-BSEP Ab 1/100*	Pulse steroids<br>Plasmapheresis<br>Anti-thymocyte globulin	Tacrolimus – MMF<br>IVIg/months<br>+/- Rituximab	**reLT**<br>Clinical remission thereafter	Lowering of anti-BSEP Ab titer from 1/100 to 1/10	

#5*	**Initial episode**	Switch to tacrolimus<br>Steroid dose increase	Tacrolimus	Clinical remission	Positive anti-BSEP Ab at similar titer (1/1)	**Alive** (25.2y)<br>Clinical remission<br>15y after relapse
	**Relapse (7.1y)**<br>*Pruritus, elevated liver enzymes*<br>*Anti-BSEP Ab 1/50*	Tacrolimus dose increase<br>Addition of MMF	Tacrolimus - MMF	BA stenosis with **reLT**<br>(10y after AIBD diagnosis)<br>Clinical remission thereafter	Lowering of anti-BSEP Ab titer from 1/50 to 1/1	
#6	**Initial episode**	Pulse steroids<br>IVIg<br>Plasmapheresis x 12	Tacrolimus - MMF<br>Steroids	Liver failure with **reLT**<br>(0.5y after AIBD diagnosis)<br>Clinical remission thereafter	Negative anti-BSEP Ab	**Alive** (13y)<br>Clinical remission<br>3.5y after relapse
	**Relapse (9.7y)**<br>*Pruritus, jaundice*<br>*Anti-BSEP Ab 1/100*	Pulse steroids<br>IVIg x 2<br>Plasmapheresis x 6<br>Rituximab x 1	Tacrolimus<br>MMF<br>Steroids (0.2 mg/kg/d)	Clinical remission	Lowering of anti-BSEP titer from 1/100 to 1/1	
#7*	**Initial episode**	Cyclosporine and steroid dose increase<br>Azathioprine initiation	Cyclosporine<br>Azathioprine	Clinical remission	Positive anti-BSEP Ab at similar titer (1/10)	**Alive** (27y)<br>Clinical remission
#8	**Initial episode**	Pulse steroids<br>Rituximab x 2<br>IVIg x 1	Tacrolimus - MMF<br>IVIg/months	Clinical remission<br>(elevation of GGT and ALT due to chronic rejection)	Negative anti-BSEP Ab	**Alive** (5y)<br>Clinical remission (no pruritus/jaundice)
#9	**Initial episode**	Plasmapheresis x10<br>Rituximab x 2	Tacrolimus - MMF<br>Steroids	**reLT** for liver failure<br>(1.1y after AIBD diagnosis)<br>Clinical remission thereafter	Lowering of anti-BSEP Ab titer from 1/200 to 1/1	**Alive** (3.8y)<br>Clinical remission<br>1.9y after relapse<br>(Absence of pruritus /jaundice)
	**Relapse (1.9y)**<br>*Pruritus, elevated sBA*<br>*Anti-BSEP Ab 1/100*	Steroid pulse<br>Plasmapheresis x 4	Tacrolimus – MMF<br>Steroids<br>Rituximab	Clinical remission<br>(elevation of GGT and ALT due to biliary obstruction)	Negative anti-BSEP Ab	
#10	**Initial episode**	Steroids, Azathioprine<br>Rituximab x 3<br>IA x 6	Tacrolimus<br>Azathioprine<br>Steroids<br>Rituximab	Clinical remission	Negative anti-BSEP Ab	**Alive** (2.1y)<br>Clinical remission

Immunological remission was defined as negative anti-BSEP serum testing. Clinical remission was defined as resolution of clinical manifestations and normalization of serum liver tests, including sBA, except if abnormalities could be explained by another complication of LT (biliary obstruction, chronic rejection). *These patients were diagnosed retrospectively. *Ab: antibodies; AIBD: alloimmune anti-BSEP liver disease; ALT: alanine*

aminotransferase; BA: biliary anastomosis; BSEP: Bile salt export pump; GGT: gamma-glutamyltransferase; IA: immunoadsorption; IVIg: intravenous immunoglobulins; LT: liver transplantation; NA: not assessed; reLT: liver retransplantation; sBA: serum bile acids; y: year
