# Supplementary material for: Post-transplant alloimmune liver disease in bile salt export pump deficiency: A multicenter study
Source: JHEP Rep. 2026 Jul 25;8(10):101969. doi: 10.1016/j.jhepr.2026.101969 (PMC13594885; doi:10.1016/j.jhepr.2026.101969)
Supplement: Supplementary file 4 [file mmc2.docx]

**JHEP Reports**

**CTAT methods**

Tables for a “Complete, Transparent, Accurate and Timely account” (CTAT) are now mandatory for all revised submissions. The aim is to enhance the reproducibility of methods.

- Only include the parts relevant to your study
- Refer to the CTAT in the main text as ‘Supplementary CTAT Table’
- Do not add subheadings
- Add as many rows as needed to include all information
- Only include one item per row

**If the CTAT form is not relevant to your study, please outline the reasons why:**

1.1 **Antibodies**

| **Name** | **Citation** | **Supplier** | **Cat no.** | **Clone no.** |
| --- | --- | --- | --- | --- |
| Anti-BSEP |  | Sigma | HPA019035 | Polyclonal  Produced in rabbit |
| Anti-C4d |  | DB Biotech | DB-107 | Monoclonal  (clone A24-T)  Produced in rabbit |
| FITC-conjugated anti-human IgG |  | Dako | F0202 | Polyclonal  Produced in rabbit |
| Anti-GFP |  | Roche | 11814460001 | Monoclonal  (clone 7.1 & 13.1)  Produced in mouse |
| Alexa Fluor™ 568 (Red)-conjugated anti-Human IgG (H+L) |  | Thermofisher Scientific | A-21090 | Polyclonal  Produced in goat |
| Alexa Fluor^TM^ 488 (Green)-conjugated anti-Mouse IgG (H+L) |  | ThermoFisher Scientific | A-11001 | Polyclonal  Produced in Goat |

1.2 **Cell lines**

| **Name** | **Citation** | **Supplier** | **Cat no.** | **Passage no.** | **Authentication test method** |
| --- | --- | --- | --- | --- | --- |
| **Can10** | Peng 2006 Cell Tissue Research (PMID 16231191) | Not applicable | Not applicable | 2 to 8 | Peng 2006 Cell Tissue Research (PMID 16231191) Fig. 7 “Can 10 polarity is functional,  and the implicated transporters are correctly localized” |

1.3 **Organisms**

| **Name** | **Citation** | **Supplier** | **Strain** | **Sex** | **Age** | **Overall n number** |
| --- | --- | --- | --- | --- | --- | --- |
|  |  |  |  |  |  |  |

1.4 **Sequence based reagents**

| **Name** | **Sequence** | **Supplier** |
| --- | --- | --- |
|  |  |  |

1.5 **Biological samples**

| **Description** | **Source** | **Identifier** |
| --- | --- | --- |
|  |  |  |

1.6 **Deposited data**

| **Name of repository** | **Identifier** | **Link** |
| --- | --- | --- |
|  |  |  |

1.7 **Software**

| **Software name** | **Manufacturer** | **Version** |
| --- | --- | --- |
|  |  |  |

1.8 **Other (*e.g*. drugs, proteins, vectors etc.)**

1.9 **Please provide the details of the corresponding methods author for the manuscript:**

Pr Emmanuel Jacquemin, MD, PhD, Service d’Hépatologie et de Transplantation Hépatique Pédiatriques, Hôpital Bicêtre, 78, rue du Général Leclerc, Le Kremlin-Bicêtre, 94275 cedex, France. Tel : +33 1 45 21 31 68 ; Fax : +33 1 45 21 28 16 ; email : [emmanuel.jacquemin@aphp.fr](mailto:emmanuel.jacquemin@aphp.fr)

**2.0	Please confirm for randomised controlled trials all versions of the clinical protocol are included in the submission. These will be published online as supplementary information.**
